# Lymph node metastasis in cancer progression: molecular mechanisms, clinical significance and therapeutic interventions

**DOI:** 10.1038/s41392-023-01576-4

**Published:** 2023-09-27

**Authors:** Haoran Ji, Chuang Hu, Xuhui Yang, Yuanhao Liu, Guangyu Ji, Shengfang Ge, Xiansong Wang, Mingsong Wang

**Affiliations:** 1 Department of Thoracic Surgery, Shanghai Key Laboratory of Tissue Engineering, Shanghai Ninth People’s Hospital, Shanghai Jiao Tong University School of Medicine, Shanghai, 200011 China; 2 Department of Ophthalmology, Shanghai Key Laboratory of Orbital Diseases and Ocular Oncology, Shanghai Ninth People’s Hospital, Shanghai Jiao Tong University School of Medicine, Shanghai, 200025 China

**Keywords:** Cancer imaging, Cancer therapy, Metastasis, Metastasis

## Abstract

Lymph nodes (LNs) are important hubs for metastatic cell arrest and growth, immune modulation, and secondary dissemination to distant sites through a series of mechanisms, and it has been proved that lymph node metastasis (LNM) is an essential prognostic indicator in many different types of cancer. Therefore, it is important for oncologists to understand the mechanisms of tumor cells to metastasize to LNs, as well as how LNM affects the prognosis and therapy of patients with cancer in order to provide patients with accurate disease assessment and effective treatment strategies. In recent years, with the updates in both basic and clinical studies on LNM and the application of advanced medical technologies, much progress has been made in the understanding of the mechanisms of LNM and the strategies for diagnosis and treatment of LNM. In this review, current knowledge of the anatomical and physiological characteristics of LNs, as well as the molecular mechanisms of LNM, are described. The clinical significance of LNM in different anatomical sites is summarized, including the roles of LNM playing in staging, prognostic prediction, and treatment selection for patients with various types of cancers. And the novel exploration and academic disputes of strategies for recognition, diagnosis, and therapeutic interventions of metastatic LNs are also discussed.

## Introduction

Lymph nodes (LNs) serve as essential components of the mammalian immune system, functioning as a barrier against systemic pathogen dissemination while facilitating the induction and maturation of specific immune responses and serving as central hubs that orchestrate interactions among immune cell populations.^1^ Malignant tumor cells, however, can hijack the lymphatic system to facilitate their metastatic dissemination throughout the body, just like thieves using the ventilation ducts to move to various rooms in a building, and LNs serve as major hubs for metastatic cell growth, secondary dissemination to other tissue compartments, and the modulation of antitumor immune responses.^2^ Lymph node metastasis (LNM) is thus a key consideration when evaluating cancer patients, as it has major implications for disease staging, clinical management, and prognostic outcomes. Recent advances in medical technologies and LNM-focused research have enabled the more effective detection and treatment of LNM. As such, this review was developed with the goal of providing a systematic overview of the physiological and anatomical characteristics of LNs, as well as the mechanistic basis for LNM and its clinical significance. These discussions are further supported by a survey of approaches to the detection, diagnosis, and therapeutic management of metastatic LNs, thereby providing a comprehensive foundation for researchers and clinicians focused on the role of the lymphatic system in cancer.

## Anatomy and physiology of lymph nodes

As central hubs for the induction of adaptive immunity, individual LNs process lymph containing local information from the tissues that drains via collecting lymphatic vessels from proximal tissues and organs.^3^ The human body contains an estimated 500 to 600 LNs that are surrounded by dense connective tissue and associated with particular nerves, lymphatic vessels, and blood vessels, with many of these LNs presenting in concentrated clusters found in specific anatomical locations.^4,5^

**Fig. 1:**
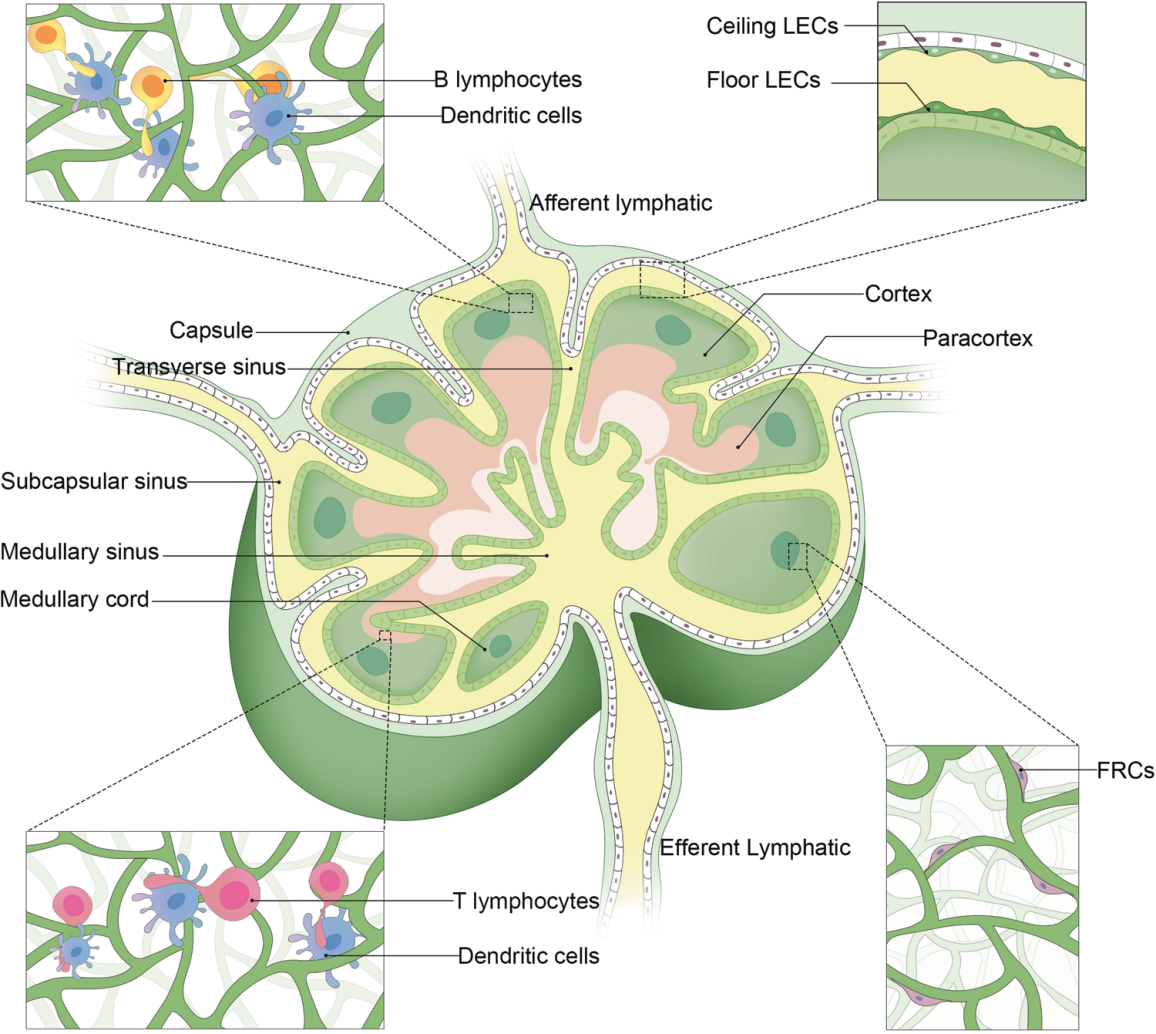
An anatomical overview of the structural characteristics of lymph nodes. LEC lymphatic endothelial cell, FRC fibroblastic reticular cell

Afferent lymphatic vessels deliver lymph to the LNs. Each LN is comprised of a complex series of lymphatic sinuses associated with organized parenchyma consisting of reticular fibers, fibroblastic reticular cells (FRCs), specialized vasculature, and a range of immune cell populations. A fibrous capsule surrounds the outer layer of each LN (Fig. 1), and connective tissue projections radiating from this capsule, known as trabeculae, extend into the node. The trabecular sinuses separate human LNs into multiple compartments, which are associated with the opening of each afferent lymphatic vessel, or each of its terminal branches, into the subcapsular sinus, though these same trabecular compartments are not evident in murine LNs.^6^ The entirety of the LN cortex and paracortex is overlaid by the subcapsular sinus, and lymphatic endothelial cells (LECs) line each sinus, forming a barrier between the lymph and the parenchymal compartment. The trabecular sinuses connect the medullary and subcapsular sinuses, with direct connections between the latter two sinuses also forming at the margins of each LN.^7^ After flowing through the medullary sinuses, lymph passes into efferent lymphatic vessels.^8^ The macrophage, B cell-, and antibody-producing plasma-cell-rich medullary cords, together with the medullary sinuses, comprise the medulla layer within LNs.^9^ In the cortex, germinal follicle-associated antigen-presenting follicular dendritic cells (DCs) can activate naïve B cells, while in the paracortical T cell zone, antigen-presenting DCs promote naïve T cell activation.^6^

Lymphocytes circulating in the blood enter LNs through specialized cuboid blood vessels known as high endothelial venules (HEVs), which exhibit a large surface area and consist of specialized blood endothelial cells (BECs).^10^ These HEVs are found in the extrafollicular cortical zone and extend into the peripheral paracortex before transitioning into standard venules upon entry into the medullary cords.^6^ HEV endothelial cells express a range of adhesion molecules that improve the rates of lymphocyte capture and entry into the associated LN, including CD34, glycosylation-dependent cell adhesion molecule 1 (GLYCAM1; only in mice), podocalyxin, endomucin, nepmucin, and 6-sulpho sialyl Lewis X.^11^

FRCs are lymphoid-specialized fibroblasts that form the structural framework for scaffolding that defines specific microenvironmental immune cell niches within LNs.^10^ These FRCs can secrete a range of extracellular matrix (ECM) proteins and form a three-dimensional conduit network system,^12^ which acts as a pipeline to monitor the status of fluid-draining peripheral tissues while exporting antibodies and other molecules produced within the local lymphoid compartment.^13^ FRCs can be further classified into functionally distinct subtypes localized to specific sites within LNs, including T cell zone FRCs (TRCs), follicular DCs (fDCs), marginal reticular cells (MRCs), and medullary FRCs (medRCs), all of which express a range of ligands, chemokines, and other cytokines important for the maintenance of LN homeostasis.^14^

## Molecular mechanisms of lymph node metastasis

**Fig. 2:**
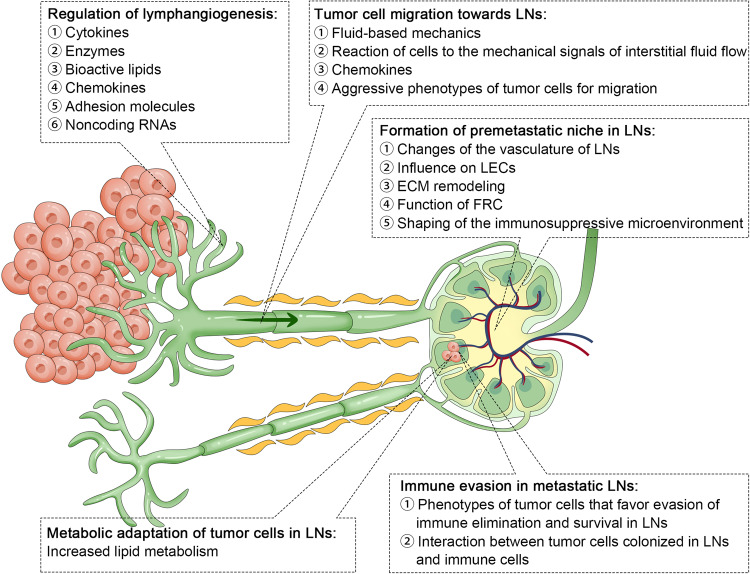
Molecular mechanisms of lymph node metastasis.^15^ LN lymph node, LEC lymphatic endothelial cell, ECM extracellular matrix, FRC fibroblastic reticular cell

Lymphatic dissemination has been documented for myriad cancer types, underscoring the need for research focused on clarifying how tumor cells migrate to and survive within LNs.^8^ Several mechanistic studies have provided detailed insight into the mechanistic basis for LNM.^15^ The ability of tumor cells to migrate to and invade LNs is often associated with the expression of particular receptor proteins and cytokines, eventually culminating in the evasion and/or suppression of normal immune function such that these malignant cells can thrive within the LN microenvironment.^3^ In this section, we provide a summary of the current understanding of the molecular basis for LNM with a particular focus on recent research progress (Fig. 2).

### Tumor cell migration toward lymph nodes

Fluid dynamics play an important role in the initiation of LNM. The blood vessels present are generally abnormally permeable and exhibit aberrant blood flow such that plasma persistently accumulates in extracellular sites and is not effectively drained owing to the compression of local lymphatic vessels by the growing tumor. This results in a pronounced increase in the intratumoral interstitial fluid pressure (IFP),^16^ forming an IFP gradient that favors the flow of interstitial fluid from tumors through the surrounding stroma and into areas of lower IFP, thereby enabling tumor cells and tumor-derived compounds to more readily access LNs.^17^ Through the establishment of a corresponding mathematical model, Jain et al.^18^ posited that this IFP gradient in the tumor margin, rather than high intratumoral IFP alone, is responsible for determining the rate of tumor cell entry into the surrounding lymphatic system and the associated induction of angiogenic activity. In further support of such a model, dynamic contrast-enhanced magnetic resonance imaging (MRI) studies have reaffirmed that higher IFP levels are detectable in the primary tumors of mice positive for metastatic progression as compared to mice without such metastases, as has further been confirmed in patients with cervical cancer positive for pelvic LNM.^19^ However, direct experimental data conclusively demonstrating the role of this IFP gradient as a driver of LNM incidence is lacking at present. In addition, as flow velocity and associated shear stress increase, cells may be more prone to growth arrest, fragmentation, and death. Even so, the lower levels of shear stress to which tumor cells are exposed in the lymphatic system, as compared to the higher flow velocities evident in blood vessels, are conducive to the survival of these malignant cells and their subsequent invasion of LNs.^16^

LNM can also be induced and regulated by the responses of tumor cells and other cells in the local microenvironment to interstitial fluid flow-related mechanical signals. Indeed, there is strong evidence for the ability of interstitial fluid flow to enhance the glioma cell invasion via the CXCR4/CXCL12 signaling axis.^20^ Interstitial fluid flow can also control breast cancer cell ameboid migration,^21^ and transcellular CCR7 ligand gradients can reportedly be shaped by this interstitial flow, thereby promoting the migratory activity of tumor cells and associated LNM.^22^ Interstitial flow can also promote macrophage M2 polarization and enable these cells to travel against this flow to access tumors, thus contributing to metastatic progression.^23^

Chemokines are also essential mediators of the ability of tumor cells to migrate toward LNs. The upregulation of CCL21 in LECs can, for example, enable tumor cells expressing CCR7 to more readily migrate toward the lymphatic vessels.^24^ Similarly, TNF, IL-1β, and LPS can induce the upregulation of CCL1, which is present in the lymphatic sinuses of LNs but absent in the peripheral lymphatic system, providing a gradient that allows tumor cells to migrate to LECs.^25^ LECs also express a variety of other chemokine ligands, including CXCL10, CXCL12, CXCL1, and CCL5, that respectively bind to CXCR3, CXCR4, CXCR2, and CCR5, thereby shaping tumor cell migration through lymphatic vessels and to LNs.^26^

Tumor cells can additionally adopt more aggressive phenotypic characteristics conducive to LN migration. The epithelial-mesenchymal transition (EMT) process entails the loss of epithelial-like characteristics such as polarization and a high degree of differentiation by tumor cells, which instead adapt invasive and migratory mesenchymal-like phenotypes, allowing these cells to more readily migrate and disseminate away from the primary tumor site.^27^ In hepatocellular carcinoma (HCC), for example, significantly elevated rates of EMT-associated features are evident in tumor samples from cases exhibiting LNM.^28^ Many different factors that can induce this EMT process have been identified and shown to be closely related to LNM. Heat shock factor 1 (HSF1), for instance, promotes the upregulation of lymphoid enhancer‐binding factor 1 (LEF1) to drive EMT induction and LNM.^29^ PRMT5 can similarly induce EMT and LNM via the modulation of Wnt4/β-catenin pathway signaling.^30^ NQO1/PKLR alters glycolytic reprogramming in tumor cells to favor EMT onset and migratory activity.^31^ Moreover, Zhao et al.^32^ have further demonstrated the upregulation of dynamin-related protein 1 (Drp1), which is associated with mitochondrial fission, in invasive breast carcinoma patients exhibiting LNM, revealing that this protein promotes the redistribution of mitochondrial to lamellipodial regions at the leading edge of cancer cells in a manner that supports migration.

### Regulation of lymphangiogenesis

More dense lymphatic vessels have been reported in peritumoral regions as compared to healthy tissues, and intratumoral lymphatic vessel growth has also been documented.^33^ The process of lymphangiogenesis is closely associated with the formation of new lymphatic vessels within sentinel lymph nodes (SLN), ultimately supporting metastatic tumor spread. The enhancement of lymphangiogenic activity is thus crucial to the effective dissemination of tumor cells into LNs, and many different lymphangiogenesis-related factors have been demonstrated to be important in the context of LNM.

Vascular endothelial growth factor (VEGF)-C and VEGF-D play key roles in the regulation of lymphangiogenesis. Cancer cell-derived VEGF-C/D activates its receptor, vascular endothelial growth factor receptor (VEGFR)-3 found on LECs to activate a protein kinase C/ERK signaling cascade which ultimately triggers the phosphorylation of AKT and the proliferation and migration of these LECs, thus promoting lymphangiogenesis.^34^ VEGFR-3 activation can also drive the activation of HOXD10, which is a homeobox family transcription factor that regulates cord-like structure formation and the migration of LECs via the control of VE-cadherin, claudin-5, and nicotinamide adenine dinucleotide phosphate oxidase 3 (NOS3) expression.^35^ The promotional effect of VEGF-C/D and VEGFR-3 on lymphangiogenesis and LNM has been documented in a range of cancer types.^36–41^

Additionally, there are many cytokines, enzymes, bioactive lipids, chemokines, adhesion molecules, and noncoding RNAs that participate in lymphangiogenesis by functioning in either a VEGF-C/D-dependent or -independent manner.

Several lymphangiogenesis- and LNM-related growth factors have been characterized to date, including fibroblast growth factor (FGF)-2, which can bind FGFR3 on the surface of LECs to promote the development of lymphatic vessels.^42^ Platelet-derived growth factor (PDGF)-BB can similarly promote vessel growth by binding to PDGF receptor α (PDGFRα) and PDGFRβ of LEC.^43^ In cholangiocarcinoma, fibroblasts have been shown to produce elevated VEGF-C levels and to promote lymphatic vessel expansion following PDGF-D stimulation.^44^ Signaling via the angiopoietin 2 (Ang2)/Tie/PI3K axis is essential for the expression of VEGFR-3 on the surface of cells, making this pathway critical in the context of lymphangiogenesis.^45^ Interactions between FGF-2 and VEGF-C are also capable of driving intratumoral lymphangiogenesis.^46^ In a model of colorectal cancer (CRC), lymphangiogenesis and metastatic growth were shown to be induced by insulin-like growth factor (IGF)-1.^47^ Epidermal growth factor (EGF) is associated with the induction of melanoma primary tumor lymphangiogenesis.^48^ There is also evidence for the ability of hepatocyte growth factor (HGF) to promote the proliferation of LECs and the development of lymphatic vessels via a VEGFR-3-independent pathway.^49^ TGF-β/Smad signaling is also central to the regulation of lymphangiogenesis, as TGF-β can promote VEGF-C upregulation via the Smad and Smad-independent AKT pathways, which has been documented in gastric cancer cells.^50^ However, TGF-β also downregulates the lymphangiogenic function of collagen and calcium-binding EGF domain-1 (CCBE1) in cancer‐associated fibroblasts (CAFs) and colorectal cancer cells by directly binding Smads to the CCBE1 gene locus.^51^ Furthermore, VEGF-D promoter activity and protein level expression can also be induced by TNF-α through ERK1/2/AP-1 pathway signaling, ultimately eliciting tube-forming activity in LECs.^52^

The interaction between interleukin (IL) and lymphangiogenesis reflects the influence of immune cells on lymphangiogenesis in LNM. For instance, IL-6 has been demonstrated to promote lymphangiogenesis in gastric cancer via the signal pathway of JAK-STAT3-VEGF-C.^53^ Similarly, IL-7 can promote the development of lymphatic vessels in lung and breast cancers by inducing VEGF-D upregulation.^54,55^ In non-small cell lung cancer (NSCLC), IL-17 has also been linked to poorer patient survival outcomes owing to its ability to drive VEGF-C secretion and lymphangiogenesis.^56^

Fatty acid synthase (FASN) is a central coordinator of lipid metabolism that is upregulated in many cancers.^57^ In melanoma cells, a link between FASN and VEGF-C/D expression has been noted, likely influencing lymphatic vessel permeability.^58^ FASN can also reportedly promote PDGF-AA and IGFBP3 secretion in cervical cancer, thus promoting lymphangiogenesis.^59^ The cyclooxygenase-2 (COX-2)/prostaglandin E2 (PGE2)/EP signaling axis is also important in this regulatory context, promoting tumor-associated lymphangiogenesis via inducing VEGF-C and VEGFR-3 upregulation in the tumor stroma in a manner that can be suppressed by COX-2 inhibitor celecoxib.^60^

Sphingosine 1-phosphate (S1P), which is generated by sphingosine kinase 1 (SphK1), has further been established as a mediator of lymphangiogenic activity in murine breast cancer metastasis model systems.^61^ By binding to S1PR1 expressed on the surface of tumor-associated macrophages (TAMs), S1P can induce lymphatic vessel development in a macrophage-dependent manner.^62^ Lysophosphatidic acid (LPA) is another lymphangiogenesis-related lipid, with both LPA_1_ and LPA_3_ reportedly inducing the upregulation of VEGF-C in prostate cancer through a calreticulin-dependent mechanism that induces lymphangiogenesis.^63^

Both chemokines and adhesion molecules are key mediators of lymphangiogenic activity. For example, integrin α4β1, which is expressed by proliferating LECs, is essential for lymphangiogenesis in the context of LNM.^64^ CCL21/CCR7 signaling can also promote enhanced VEGF-C secretion and consequent lymphatic vessel growth.^65^ Bieniasz-Krzywiec et al.^66^ determined that binding interactions between podoplanin on the surface of TAMs and galectin 8 (GAL8) expressed by LECs can promote pro-migratory integrin β1 activation, thereby enabling TAMS to migrate toward and adhere to LECs, facilitating TAM‐mediated lymphangiogenesis. The adhesion molecule CD146 is expressed by endothelial cells and many different tumor types, and functions as a receptor for VEGF-C that can regulate lymphangiogenesis.^67^

A growing body of research has also documented the importance of ncRNAs as regulators of lymphangiogenic and metastatic activity. For example, He et al.^68^ revealed a role for the long noncoding RNA (lncRNA) bladder cancer-associated transcript 2 (BLACAT2) as a regulator of VEGF-C expression through its ability to associate with the core H3K4 methyltransferase complex subunit WDR5, ultimately inducing bladder cancer-related lymphangiogenesis. Chen et al.^69^ further identified LNM-associated transcript 2 (LNMAT2) as a lncRNA packaged in exosomes that can stimulate LEC migration, tube formation, and bladder cancer-associated lymphangiogenesis and LNM. Zheng et al.^70^ investigated the biological effects of novel triple-negative breast cancer (TNBC) lymph node-associated lncRNA LINC00857, also known as lncRNA highly upregulated in metastatic TNBC (HUMT). They found that HUMT could recruit Y-box binding protein 1 (YBX1) to form a novel transcriptional complex capable of activating forkhead box k1 (FOXK1) to promote VEGF-C upregulation. Circular RNAs (circRNAs) can exert similar regulatory roles in this context, as in the case of circEHBP1, which reportedly favors bladder cancer-associated lymphangiogenesis through the miR-130a-3p/TGFβR1/VEGF-D signaling axis.^71^ Meanwhile, circNFIB1 (hsa_circ_0086375) can inhibit lymphangiogenesis and LNM via the miR-486-5p/PIK3R1/VEGF-C axis in pancreatic cancer.^72^

### Formation of premetastatic niche in lymph nodes

Secondary metastatic tumor development is thought to be enabled by the establishment of a premetastatic niche, which consists of a microenvironment shaped and seeded by a range of tumor-derived factors such that it is better suited to supporting the proliferation and survival of disseminated malignant cells.^8^ Premetastatic niche formation is controlled by the coordinated effects of cytokines, chemokines, and extracellular vesicles (EVs).

Changes in the LN vasculature are central to the process of premetastatic niche establishment within these LNs. Exosomes produced by melanoma cells can be home to SLNs, wherein they induce the production of VEGF-B, HIF-1α, and other angiogenic growth factors that induce local vascular proliferation.^73^ Particularly, HEV remodeling is a characteristic process in the formation of premetastatic niches in LNs, with SLNs reportedly exhibiting increased HEV density prior to tumor cell arrival.^74^ The tall endothelial cells within HEVs undergo morphological changes such that they exhibit flat endothelial cell phenotypes. These changes coincide with the remodeling of HEVs from thick-walled endothelial vessels with a small lumen to thin-walled vessels with a larger lumen, causing a functional shift away from lymphocyte recruitment in favor of greater blood flow that is conducive to metastatic tumor cell arrival.^75^ Bone morphogenetic protein-4 (BMP-4) loss has also been linked to this thin-walled HEV remodeling,^76^ as has CCL21 dysregulation in perivascular FRCs and associated CCL21-saturated lymphocyte accumulation.^77^

Premetastatic niche formation is also associated with changes in LEC characteristics. For example, The activation of integrin α4β1 on LECs through a VEGF-C/PI3Kα-associated pathway can promote LN remodeling via the expansion of the local lymphatic endothelium and the enhanced capture of metastatic cells expressing vascular cell adhesion molecule 1 (VCAM-1).^78^ CAFs expressing high periostin levels that are present within the metastatic LN-associated stroma can interfere with the integrity of the lymphatic endothelial barrier as a consequence of LEC-specific integrin‐FAK/Src‐VE‐cadherin pathway activation, thus promoting LNM.^79^ Integrin αIIb is also upregulated in LECs present within tumor-draining LNs (TDLNs), enabling these LECs to adhere to fibrinogen in a manner that may improve metastatic tumor cell adherence and survival.^80^

The remodeling of the ECM is also integral to the process of premetastatic niche formation in various organs.^81^ Exosomes produced by tumor cells can promote the upregulation of a range of ECM-associated factors that can ultimately better entrap migratory tumor cells within SLNs.^73^ FRCs are the primary cell type responsible for ECM production within LNs, and they may thus serve as particularly important mediators of ECM remodeling within LNs in the context of LNM.^82^ The production of laminin α4 by FRCs, for example, can enhance T cell migration while promoting the differentiation of Tregs and interfering with the development and activation of other T cell subsets, contributing to the formation of a tolerogenic LN niche.^83^ Evidence regarding the ability of FRCs to directly regulate the ECM in premetastatic LNs, however, is currently lacking, underscoring a need for further research.

FRCs can also shape the premetastatic niche through various other mechanisms. Riedel et al.^84^ showed that before metastatic colonization, tumor-derived lactic acid could drain to LNs, contributing to IL-7 downregulation and altered FRC mitochondrial function. IL-1 production by melanoma cells can suppress FRC contractility through JAK1/STAT3 pathway inhibition, with the consequent relaxation of the 3D FRC network, better-enabling melanoma cells to invade this niche.^85^ Transcriptional analyses of FRCs in TDLNs have provided evidence of microenvironmental reprogramming, including the expansion and structural reorganization of stromal compartments and the suppression of CCL21 and IL-7 production by FRCs, enabling greater tumor cell immune evasion and impaired immune cell homing.^86^

The ability of tumor cells to seed LNs is strongly dependent on the establishment of an immunosuppressive microenvironment. Strikingly, single-cell studies focused on human prostate cancer progression have revealed that immunological changes precede metastatic progression.^87^ Otto et al.^88^ collected tumor regional and distant lymph nodes from patients with esophageal cancer and found that premetastatic LNs associated with more advanced tumors exhibited characteristics consistent with a greater degree of immunosuppression. In patients with breast carcinoma, lower levels of Th1 response induction and DC maturation have also been reported in SLNs before LNM.^89^ Comparative analyses of breast cancer patient SLNs have further revealed that increased Treg and myeloid-derived suppressor cell (MDSC) activity occurs before nodal involvement, together with the general anergy of T cells within these LNs as a consequence of impaired LN-resident DC activation.^90^ TAM accumulation in gastric cancer-associated premetastatic LNs can also reportedly facilitate tumor progression by promoting the production of VEGF and MMP while also suppressing antitumor immune responses by releasing cytokines, including IL-10.^91^ In a Lewis lung carcinoma (LLC) metastasis model system, DCs present in LN subcapsular regions were found to induce the recruitment of Tregs during LNM through the COX-2/EP3-dependent production of stromal cell-derived factor 1 (SDF-1).^92^ In a mouse mammary tumor model system, high levels of immunosuppressive Treg accumulation were also noted during primary tumor growth in compartments, including TDLNs, wherein these cells were able to suppress NK cell activation and support more robust LNM.^93^ In the context of gastric cancer LNM, IL-8 production by CAFs can induce CD8 + T cells to upregulate PD-1 within the premetastatic niche, thereby hamstringing the induction of antitumor immunity. The intratumoral upregulation of S1PR1/STAT3 can also spur the production of S1PR1/STAT3-activating factors by various cells within LNs and other premetastatic sites, thereby better enabling myeloid cell colonization and consequent metastasis.^94^ B cells can also reportedly shape the process of premetastatic niche development. Substantial B cell recruitment and proliferation in TDLNs can be induced by primary tumor cells, potentially resulting in the production of pathogenic antibodies targeting HSPA4/ITGB5 that can activate Src/NF-κB signaling within tumor cells, ultimately supporting metastasis via the CXCR4/SDF-1α axis.^95^ Neutrophils similarly serve as regulators of premetastatic niche formation, with IL-17 produced by γδ T cells serving to promote the systemic expansion and polarization of neutrophils in a granulocyte colony-stimulating factor (G-CSF)-dependent manner, ultimately suppressing CD8 + T cells activity and promoting LNM.^96^

### Metabolic adaptation of tumor cells in lymph nodes

After reaching the LNs, tumor cells undergo a series of metabolic changes to adapt to the microenvironment. LNs are rich in lipids, and it has been confirmed that tumor cells present in metastatic LNs reportedly exhibit increased reliance on lipid metabolism, stimulating signaling via the fatty acid oxidation (FAO) and peroxisome proliferator–activated receptor (PPAR)-αpathways, allowing these cells to accumulate larger volumes of FAs as compared to the primary tumor cells.^97^ Fatty acid-binding protein 5 (FABP5) can reportedly reprogram FA metabolism in cervical cancer in a manner that favors FA synthesis and lipolysis, thus supporting LNM.^98^ Shang et al.^99^ confirmed that the lncRNA LNMICC is capable of recruiting the nuclear factor NPM1 to FABP5, which could be directly targeted and suppressed by miR-190, thus promoting LNM. The overexpression of CD36, a receptor at the top of the signaling cascade that takes up lipids from the extracellular environment, greatly promotes LNM in cell lines or patient-derived cells with low metastatic potential oral carcinomas, with penetrance increasing from less than 20% to 75–80%.^100^ Furthermore, bile acids can additionally trigger yes-associated protein (YAP)-dependent metabolic changes in tumor cells that ultimately favor their metabolic shift towards increased FAO activity.^97^ Jia et al.^101^ found that RPRD1B, a transcriptional coactivator, facilitates FA metabolism and promotes LNM via the c-Jun/c-Fos sterol regulatory element-binding protein 1 (SREBP1) axis, which is enhanced by lncRNA nuclear enriched abundant transcript 1 (NEAT1).

### Immune evasion in metastatic lymph nodes

Tumor cells that successfully colonize LNs generally exhibit phenotypes conducive to the evasion of immune-mediated detection. Major histocompatibility complex (MHC) expression is crucial for the presentation of tumor cell-derived antigens to local immune cells, and many tumor cells exhibit decreased MHC expression such that they can avoid T cell-mediated elimination.^102,103^ Yoshii et al. observed MHC downregulation in metastatic LNs compared with its expression in primary lesions in clinical samples of gastric carcinoma.^104^ The loss of MHC-I expression has also been documented in the TDLNs of many breast cancer patients.^105^ Consistently, the expression of higher MHC-II levels in breast cancer has been linked to a lower risk of lymphovascular invasion and better prognostic outcomes in patients with LNM.^106^ In a murine melanoma LNM model system, however, strong upregulation of MHC-I-encoding genes was observed, thereby enabling tumor cells to evade NK cell-mediated cytotoxicity normally induced by the loss of MHC-I.^107^ Therefore, the precise role that MHC proteins play in shaping the process of LNM thus warrants further research. Moreover, PD-L1 upregulation has been noted in the TDLNs for various tumor types, contributing to the suppression of T-cell responses and enhanced LNM.^107,108^

The ability of tumor cells within LNs to interact with immune cells shapes the consequent induction of immune tolerance, thereby enabling tumor cells to evade immune-mediated killing such that distant metastases can continue developing. LN metastases have been demonstrated to suppress NK cell-mediated cytotoxicity in cases of early-stage head and neck cancer.^109^ These metastatic cells in LNs can resist the cytotoxic effects of CD8^+^ T cells while promoting the differentiation of antigen-specific naïve CD4^+^ T cells into Tregss.^107^ In breast cancer TDLNs, higher frequencies of Tregs have been noted in the context of nodal invasion. These Tregs also express elevated co-inhibitory/stimulatory receptor protein levels relative to effector cells and function as mediators of immunosuppressive activity within the LNs.^110^ Tregs also secrete TGF-β1, which promotes the Smad2/3/4-mediated upregulation of the oncogenic receptor protein IL-17rb on cancer cells within the TDLNs, facilitating sustained oncogenic progression.^111^

## Clinical significance of lymph node metastasis

**Fig. 3:**
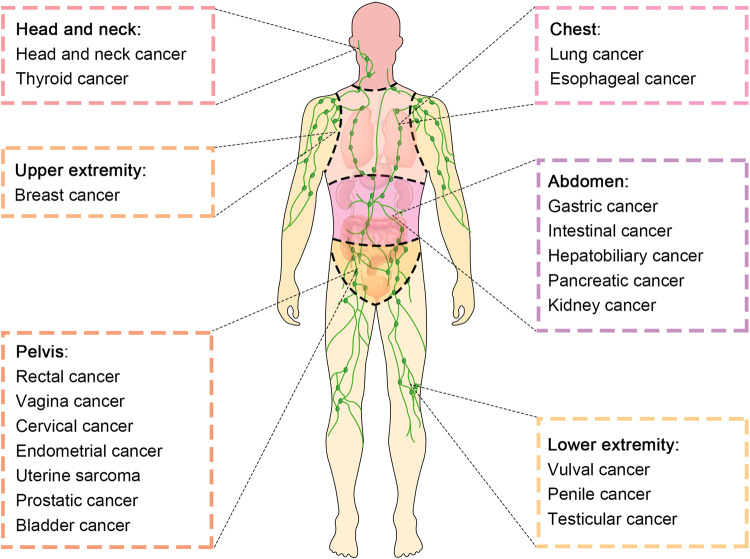
Representative tumors types involving lymph node metastasis in different anatomical regions

LNM is a key parameter that is taken into consideration when evaluating cancer patients, with the ability of LNM to strongly predict cancer patient survival being a subject of intensive scrutiny and debate.^112^ The presence of cancer cells in LNs could not only reflects the metastatic ability of the primary tumor, but also leave and colonize in distant organs.^113^ Multiple pre-clinical reports have highlighted the ability of metastatic cells from LNs to migrate to distant sites.^114–116^ Naxerova et al.,^117^ for example, conducted an analysis of 213 archived biopsy samples from 17 CRC patients, and ultimately found that the lymphatic and distant metastases developed from separate primary tumor subclones in 65% of cases, while they exhibited a shared subclonal origin in the remaining 35% of cases. This suggests that, at least in certain cancer types, metastatic tumor cells within LNs may subsequently disseminate to other organs.^3^ Lymphatic staging thus plays a key role in the evaluation of cancer patients, as when it is accurately performed, this can ensure that patients receive the most appropriate therapies in order to maximize their odds of positive clinical outcomes.^118^ If understaging occurs, patients may be subjected to unnecessary local surgery/radiotherapy or the omission of appropriate systemic therapy, whereas the opposite may occur in cases of overstaging as a result of inadequate LN staging.^119^ The most widely used cancer staging system in the world at present is the 8^th^ edition of the tumor-node-metastasis (TNM) system established by the American Joint Committee on Cancer (AJCC), which serves as a benchmark for tumor patient classification, treatment selection, and prognostic evaluation.^120–122^ The TNM system takes several factors into consideration, including the morphology and location of the primary tumor, the number and location of involved regional LNs, and the absence or presence of distant metastases. In the following section, we provide a detailed overview of the clinical significance of LNM in different anatomical sites (Fig. 3).

### Lymph nodes of the head and neck

**Fig. 4:**
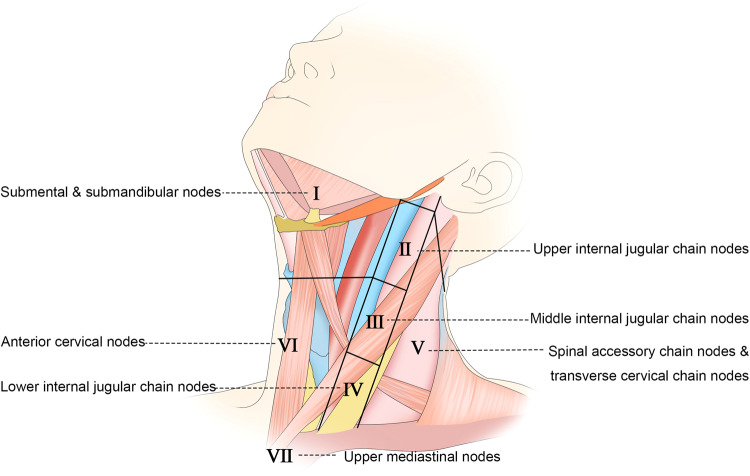
Schematic diagram of the neck showing the AJCC classification of the cervical nodes^124^

The cervical region of the head and neck harbors an estimated 150–300 LNs, the majority of which are located at the border between the head and neck. As such, LNM evaluation is particularly important in cases of thyroid or head and neck cancer.^123^ The AJCC classification system separates the cervical LNs into seven levels, with levels I, II, III, IV, V, VI, and VII, respectively, including the submental and submandibular LNs, upper internal jugular chain LNs, middle internal jugular chain LNs, lower internal jugular chain LNs, spinal accessory and transverse cervical chain LNs, anterior cervical nodes, and upper mediastinal LNs (Fig. 4).^124^ Nodes not included in these levels are instead referred to by their nodal groups, and include the periparotid, postauricular, suboccipital, retropharyngeal, and buccinator LNs.^125^

In thyroid carcinoma patients, patient nodal status is assessed based on the regions of metastatic LNs and whether they exhibit ipsilateral, bilateral, or contralateral locations.^126^ In head and neck cancer, however, factors including LN size, LN staging, location (bilateral/contralateral/ipsilateral), and extranodal extension (ENE) are taken into consideration, with ENE being characteristic of aggressive tumors such as cancer of the oral cavity.^127^ ENE status is associated with marked differences in head and neck cancer patient survival, and it has been best studied in this cancer type as a result.^128^ The left supraclavicular node, also known as Virchow’s node, is a terminal thoracic duct LN and a common site of distal metastasis in patients with abdominal and pelvic cancers that can influence treatment planning. Supraclavicular nodes are also relevant when diagnosing metastatic thoracic malignancies, although thoracic tumors do not exhibit any specific preference for the left or right supraclavicular nodes.^129^

When treating head and neck cancer patients, multidisciplinary assessment is vital, given that the most appropriate treatment options vary as a function of disease stage, anatomical region, and accessibility for surgical treatment.^130^ Cervical LN management comprises an important aspect of the surgical treatment of affected patients, with the choice of selective or comprehensive neck dissection being performed in accordance with preoperative clinical staging results.^131^ When selective neck dissection is performed, the target region is chosen based on the primary tumor location and the risk of occult metastasis within the corresponding nodal basin. The ipsilateral side of the neck generally exhibits the greatest risk of LNM. Bilateral neck dissection is generally necessary for tumors situated in areas that are often subject to bilateral lymphatic drainage, such as the base of the tongue, palate, supraglottic larynx, hypopharynx, nasopharynx, and deep pre-epiglottic space. In patients exhibiting advanced lesions involving the floor of the anterior tongue, the floor of the mouth, or alveolus that approximate or cross the midline, contralateral selective/modified neck dissection is required.^127^ In patients with advanced disease and regional LNM, chemoradiotherapy or radiotherapy can offer benefits both in the form of adjuvant treatment after surgical resection and neck dissection, or as the primary treatment in cases of unresectable disease.^130^

### Lymph nodes of the upper extremity

The axillary LNs are closely associated with breast cancer, and include the apical axillary (infraclavicular), interpectoral (Rotter’s), central axillary, lateral axillary (humeral), posterior axillary (subscapular), and anterior axillary (pectoral) nodes. The ipsilateral axilla is the predominant site of mammary lymphatic drainage, while ~3% of the mammary lymph drains to the internal mammary chain LNs, and even less drains to other LNs that can include the intercostal, interpectoral, periclavicular, paramammary, contralateral breast, or abdominal nodes.^132^ LN status is among the most important prognostic factors in breast cancer patients, with LNM being evident in approximately one in three patients and associated with a worse prognosis as compared to node-negative status.^133^ The LN staging for breast cancer in the AJCC 8^th^ edition is determined by the status of axillary, internal mammary, and supraclavicular LNs.^134^ The axillary LNs are separated into levels I, II, and III. Level I LNs are located lateral to the lateral border of the pectoralis minor muscle, while level II LNs, which include the Rotter nodes, are positioned beneath the pectoralis minor muscle between its lateral and medial borders, and level III LNs, which are associated with a poorer prognosis, are infraclavicular LNs positioned medial to the medial margin of the pectoral minor muscle and beneath the clavicle.^135^

SLN biopsy (SLNB) is routined performed when staging breast cancer patients and selecting appropriate treatments.^136^ In two different randomized clinical trials (American College of Surgeons Oncology Group-Z0011 and International Breast Cancer Study Group 23-01),^137,138^ no clinical improvements were noted for breast cancer patients that underwent additional axillary surgery beyond the sentinel TDLN, with axillary LN dissection (LND) instead often resulting in severe complications such as shoulder dysfunction, dysaesthesia, and lymphoedema.^139,140^ The predictive utility and accuracy of SLNB have since been demonstrated in multiple reports such that SLNB has replaced LND as the standard approach to evaluating the axillary LN status of clinical LN-negative breast cancer patients.^139,141^

In the randomized “After Mapping of the Axilla: Radiotherapy or Surgery” clinical trial, excellent axillary control was successfully achieved through both axillary LND and axillary radiotherapy,^142^ with comparable overall and disease-free survival rates in these two treatment groups.^143^ This suggests that axillary radiotherapy in primary breast cancer patients with positive anterior LN biopsy results is as efficacious as axillary LND. Prospective randomized trials conducted in Denmark and Canada have further demonstrated that post-mastectomy adjuvant chemotherapy treatment can improve patient survival while reducing the risk of local recurrence, reflecting the value of adjuvant chemotherapy and radiotherapy as a means of preventing tumor recurrence and fatal metastasis.^144,145^ Neoadjuvant chemotherapy is also increasingly being employed as a treatment option in breast cancer patients,^146–149^ and it has been shown to reduce the likelihood of axillary LND in both patients undergoing mastectomy and patients with biopsy-confirmed LNM.^150^

The epitrochlear LNs positioned in the subcutaneous connective tissue on the medial elbow 4–5 cm above the humeral epitrochlea are also superficial nodes present in the upper extremities.^151^ The epitrochlear and axillary LNs are often regarded as “in transit” targets for tumor cells derived from primary tumors situated on the hand, wrist, or forearm in cases of rhabdomyosarcoma or melanoma, and the association between epitrochlear LN status and prognostic outcomes should be taken into consideration.^152,153^

### Lymph nodes of the chest

LNs situated on the chest wall can serve as sites for metastatic tumor progression. Much like axillary LNs, the internal mammary nodes, also referred to as the parasternal nodes, are regarded as first-tier sites for breast cancer drainage. The chain of internal mammary LNs spans the first to the sixth intercostal spaces.^135^ An estimated 4–9% and 16–65% of axillary node-negative and axillary node-positive patients, respectively, exhibit internal mammary LN metastasis. As a result, the treatment of breast cancer often entails both surgical axillary clearance and the elective irradiation of non-dissected internal mammary and medial supraclavicular LNs in patients exhibiting axillary node positivity or tumors that are medially or centrally located.^151^ These intercostal nodes can also rarely serve as sites of extra-axillary breast tumor metastasis, although they rarely have any impact on treatment selection or patient prognosis.^154^ These intercostal nodes can also be sites of metastatic involvement in patients diagnosed with malignant pleural mesothelioma, which is among the deadliest forms of cancer.^155,156^

**Fig. 5:**
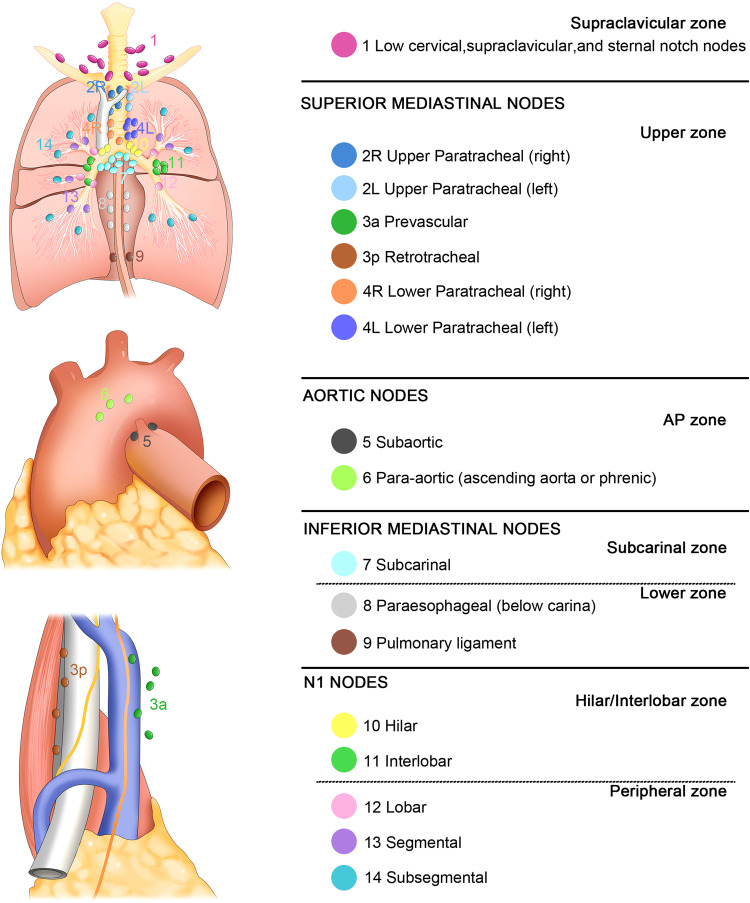
The IASLC LN map for lung cancer^160^

A growing number of studies have explored the associations between thoracic visceral tumors, such as esophageal and lung cancers, and thoracic LNs. The pulmonary lymph drains from the lungs to the LNs proximal to the lobar bronchi, with subsequent drainage to extrapulmonary tracheobronchial LNs. The efferent lymphatics of these nodes, in turn, extend to the left and right mediastinal lymph trunks, potentially draining into the thoracic duct or directly into the ipsilateral brachiocephalic vein.^157^ Nodal status is among the most reliable prognostic indicators in lung cancer patients, making it vital to the selection of optimal therapeutic approaches.^158,159^ The International Association for the Study of Lung Cancer (IASLC) established an LN map in 2009 that provides detailed anatomic definitions for all LN stations (numbered 1–14), which are grouped into the supraclavicular, upper, aortopulmonary (AP), subcarinal, lower, hilar/interlobar, and peripheral zones, enabling more reliable analyses of the association between these nodes and survival outcomes (Fig. 5).^160^ According to the TNM staging system, lung cancer nodal status of lung cancer is based upon the anatomical locations of metastatic nodes rather than on the number thereof, in which N1 refers to metastasis in ipsilateral peribronchial and/or ipsilateral hilar LNs and intrapulmonary nodes (including involvement by direct extension), N2 refers to metastasis in ipsilateral mediastinal and/or subcarinal LN(s), and N3 refers to metastasis in contralateral mediastinal, contralateral hilar, ipsilateral or contralateral scalene, or supraclavicular LN(s); whereas in esophageal cancer, which shares many of the same lymphatic pathways within the thoracic cavity, nodal status is only based on the number of metastatic nodes.^158^ In a multivariate analysis of 3,971 patients with NSCLC who underwent complete resection and systematic LND, the nodal status of different LN zones and stations were identified as independent predictors of recurrence and overall survival.^161^ Both the number and locations of metastatic nodes offer prognostic significance in patients with NSCLC, with more proximal N1 station involvement correlating with a worse prognosis.^162^ N1 and N2 staging based on the involvement of nodes from one or more stations has yet to be adopted, as results derived from pathologically staged tumors could not be validated at clinical staging, and the degree of examination thoroughness can readily impact the results of staging performed according to the number of involved stations.^163^

In stage I or II NSCLC patients, surgery offers the greatest chance of curative outcomes.^164^ The current NCCN guidelines for NSCLC patients indicate that N1 and N2 node resection and mapping should be standard in patients undergoing lung tumor resection, with a minimum of three N2 stations undergoing sampling or complete LND being performed. For patients undergoing respective treatment of stage IIIA (N2) NSCLC, formal ipsilateral mediastinal LND is recommended.^165^ In cases of pathologic N1 disease, current recommendations suggest the administration of a platinum-based dual adjuvant chemotherapy regimen after surgery.^166^ Neoadjuvant chemotherapy and resection can provide benefits to stage IIIA (N2) patients exhibiting preoperative mediastinal LN negativity and one positive node <3 cm in size, with definitive chemoradiotherapy otherwise being recommended.^167^ Surgery is not recommended for individuals diagnosed with N3 disease, who should instead be administered systematic regimens consisting of some combination of radiotherapy, chemotherapy, immunotherapy, and targeted therapy.^168^

### Lymph nodes of the abdomen

**Fig. 6:**
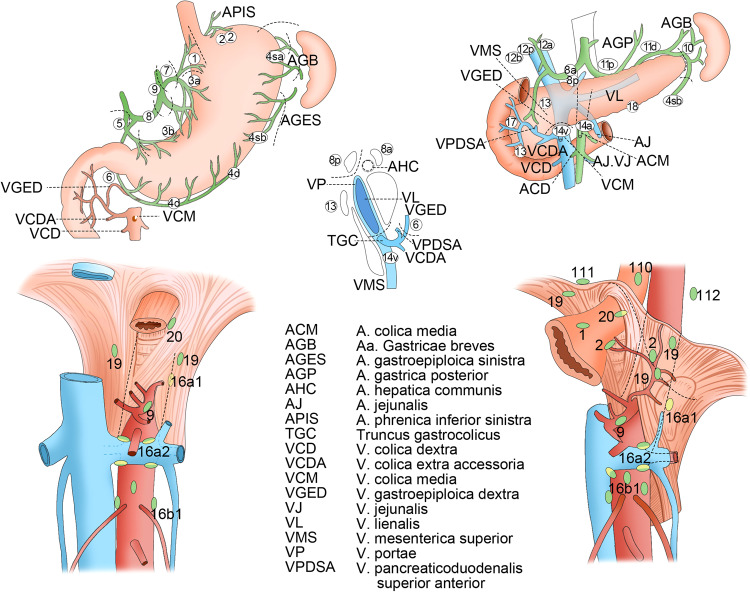
Location of LN stations for nodal status evaluation of gastric cancer^175^

Abdominal lymphatic drainage pathways parallel the vessels that supply blood to or drain blood from organs. Many abdominal LNs are located in the mesentery, mesocolon, and peritoneal ligaments, providing sites for the potential metastasis of gastric, hepatic, renal, pancreatic, intestinal, or gallbladder tumors. Here, gastric cancer was selected as a representative tumor type. There are four primary zones of gastric lymph drainage. The superior gastric nodes that surround the left gastric artery are responsible for the lymphatic drainage of the proximal portion of the stomach, whereas the suprapyloric nodes drain the lesser curvature, the subpyloric nodes drain the right gastroepiploic vessels, and the pancreaticosplenic nodes drain the stomach body and fundus along a course that parallels the left gastroepiploic and short gastric arteries. All lymph draining from the stomach ultimately flows to the celiac nodes situated at the base of the celiac artery.^169^ As the stomach has an extensive lymphatic network,^170,171^ LNM is a common finding in gastric cancer patients.^172^ Even in patients with early gastric cancer, the incidence of LNM is approximately 10%,^173^ while the incidence of bloodstream metastases is just 0.2% of these same patients.^174^ Per the Japanese Gastric Cancer Association (JGCA) anatomical definitions of LN stations (Table 1 and Fig. 6), LN stations 1–12 and 14 v are defined as regional gastric LNs, with metastases to any other nodes resulting in M1 classification.^175^ In the AJCC 8th edition TNM staging of gastric cancer, the number of metastatic nodes is used to determine nodal status, including N1 (1–2 regional metastatic LNs), N2 (3–6 regional metastatic LNs), and N3 (7+ regional metastatic LNs). N3 cases can also be subdivided into N3a (7–15 metastatic regional LNs) and N3b (16+ metastatic regional LNs).^176^

**Table 1:** The JGCA anatomical definitions of LN stations for nodal status evaluation of gastric cancer^175^

No.	Definition
1	Right paracardial LNs, including those along the first branch of the ascending limb of the left gastric artery
2	Right paracardial LNs, including those along the first branch of the ascending limb of the left gastric artery
3a	Lesser curvature LNs along the branches of the left gastric artery
3b	Lesser curvature LNs along the 2nd branch and distal part of the right gastric artery
4sa	Left greater curvature LNs along the short gastric arteries (perigastric area)
4sb	Left greater curvature LNs along the left gastroepiploic artery (perigastric area)
4d	Rt. greater curvature LNs along the 2nd branch and distal part of the right gastroepiploic artery
5	Suprapyloric LNs along the 1st branch and proximal part of the right gastric artery
6	Infrapyloric LNs along the first branch and proximal part of the right gastroepiploic artery down to the confluence of the right gastroepiploic vein and the anterior superior pancreatoduodenal vein
7	LNs along the trunk of left gastric artery between its root and the origin of its ascending branch
8a	Anterosuperior LNs along the common hepatic artery
8p	Posterior LNs along the common hepatic artery
9	Celiac artery LNs
10	Splenic hilar LNs, including those adjacent to the splenic artery distal to the pancreatic tail, and those on the roots of the short gastric arteries and those along the left gastroepiploic artery proximal to its 1st gastric branch
11p	Splenic hilar LNs, including those adjacent to the splenic artery distal to the pancreatic tail, and those on the roots of the short gastric arteries and those along the left gastroepiploic artery proximal to its 1st gastric branch
11d	Distal splenic artery LNs from halfway between its origin and the pancreatic tail end to the end of the pancreatic tail
12a	Hepatoduodenal ligament LNs along the proper hepatic artery, in the caudal half between the confluence of the right and left hepatic ducts and the upper border of the pancreas
12b	Hepatoduodenal ligament LNs along the bile duct, in the caudal half between the confluence of the right and left hepatic ducts and the upper border of the pancreas
12p	Hepatoduodenal ligament LNs along the portal vein in the caudal half between the confluence of the right and left hepatic ducts and the upper border of the pancreas
13	LNs on the posterior surface of the pancreatic head cranial to the duodenal papilla
14v	LNs along the superior mesenteric vein
15	LNs along the middle colic vessels
16a1	Paraaortic LNs in the diaphragmatic aortic hiatus
16a2	Paraaortic LNs between the upper margin of the origin of the celiac artery and the lower border of the left renal vein
16b1	Paraaortic LNs between the lower border of the left renal vein and the upper border of the origin of the inferior mesenteric artery
16b2	Paraaortic LNs between the upper border of the origin of the inferior mesenteric artery and the aortic bifurcation
17	LNs on the anterior surface of the pancreatic head beneath the pancreatic sheath
18	LNs along the inferior border of the pancreatic body
19	Infradiaphragmatic LNs predominantly along the subphrenic artery
20	Paraesophageal LNs in the diaphragmatic esophageal hiatus
110	Paraesophageal LNs in the lower thorax
111	Supradiaphragmatic LNs separate from the esophagus
112	Posterior mediastinal LNs separate from the esophagus and the esophageal hiatus

Copyright 2011, The International Gastric Cancer Association and The Japanese Gastric Cancer Association

**Fig. 7:**
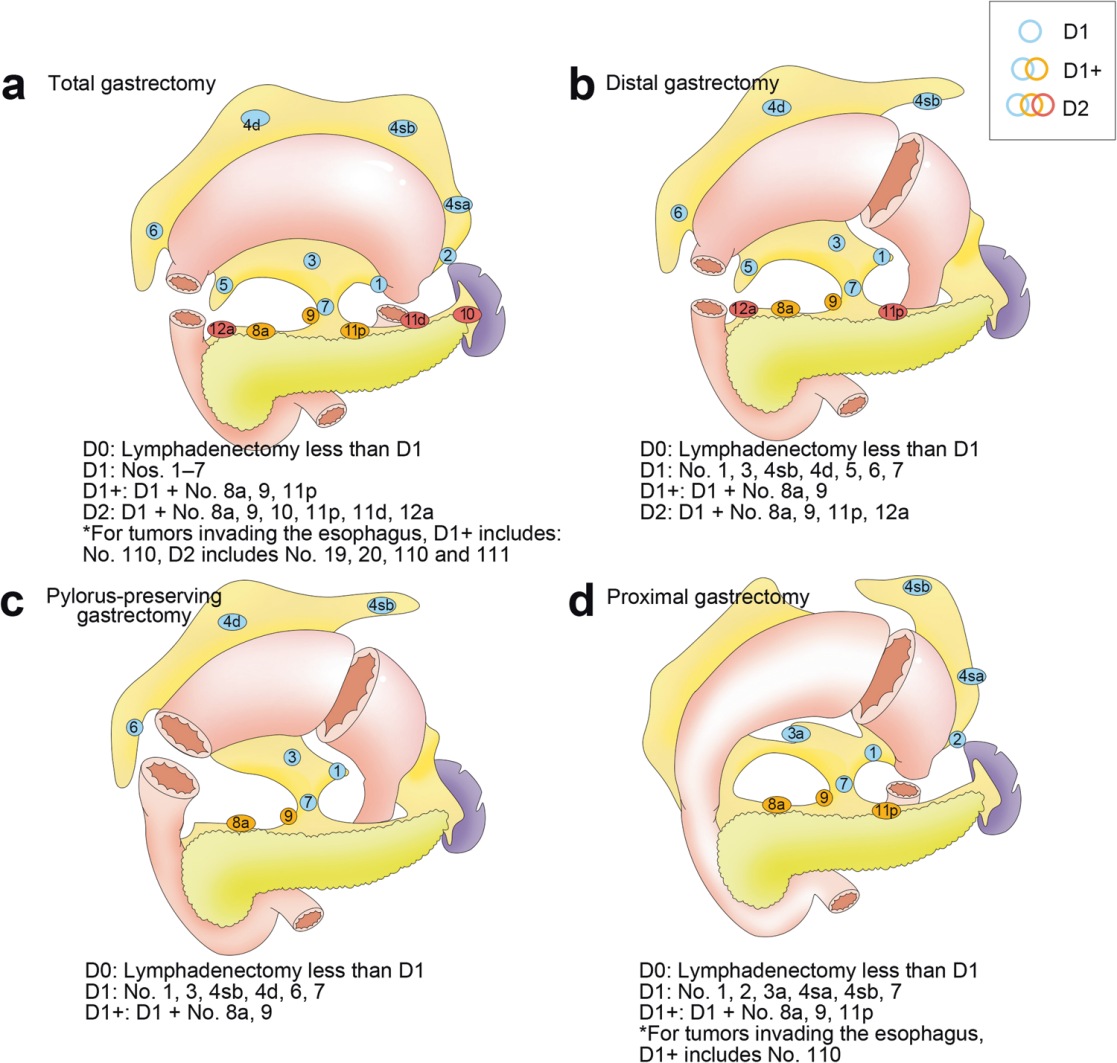
A schematic overview of lymphadenectomy for standard gastric cancer surgery.^425^ **a** The extent of lymphadenectomy after total gastrectomy. **b** The extent of lymphadenectomy after distal gastrectomy. **c** The extent of lymphadenectomy after pylorus-preserving gastrectomy. **d** The extent of lymphadenectomy after proximal gastrectomy

When the LNM of gastric cancer is limited to the perigastric LNs, it can generally be cured by lymph node dissection (LND).^177^ However, the more appropriate extent of LND in gastric cancer patients remains somewhat controversial. While incomplete LND has the potential to contribute to tumor recurrence as a result of inadequate tumor clearance, broader LND procedures are associated with a greater risk of postoperative complications.^178^ At present, the “D” numbering system is used when discussing the extent of LND in gastric cancer patients, which is classified as D1, D1+, D2, or D3. Under this system, D1 and D2 respectively correspond to the complete dissection of group 1 and 2 LNs. The locations of these nodes, however, are defined by the surgery type (distal or total gastrectomy), rather than by the location of the primary tumor (Fig. 7). D3 entails the resection of all D2 LNs, together with the removal of well-defined abdominal paraaortic and hepatoduodenal nodes. In patients with T1N0 disease, D1 or D1+ are recommended, whereas D2 is the approach of choice for individuals with T2 to T4 disease, and D3 LNM is not a recommended approach.^179^ Prophylactic LND has been validated as a treatment option in gastric cancer patients, and those early gastric cancer patients that undergo gastrectomy and prophylactic LND can exhibit 5-year survival rates upwards of 98%.^172^ D2 LND is a standard surgical approach in individuals diagnosed with resectable advanced gastric cancer.^177^

Patients with extensive lymph node metastases (ELM) from gastric cancer generally have a poor prognosis; however, preoperative neoadjuvant chemotherapy may improve surgical outcomes. The phase II JCOG 0001 study focused on gastric cancer patients with ELM employed a neoadjuvant chemotherapy regimen composed of irinotecan and cisplatin followed by gastrectomy and expanded LND (including PAND).^180^ The study was terminated because three treatment-related deaths were reported. Subsequent analyses of the trial data revealed a median survival time of 14.6 months and a 27% 3-year survival rate, which was higher than expected, although the 15% pathological remission rate fell below expectations. In the phase II JCOG 0405 study of surgery following neoadjuvant chemotherapy,^181^ following a neoadjuvant S-1 + cisplatin regimen and laparoscopy to exclude peritoneal metastases, gastrectomy with expanded LND was associated with respective 3- and 5-year survival rates of 59 and 53%, well above expected levels. Based on JCOG 0405, the JCOG1002 trial added docetaxel to the S-1 plus cisplatin with the goal of attaining better response and survival rates,^182^ although this regimen was ultimately associated with a response rate below that from the JCOG 0405 study, albeit with a 62% 3-year survival rate. In light of these results, D2-plus PAN dissection following treatment with a regimen consisting of S-1 plus cisplatin offers promise as a tentative treatment standard for individuals diagnosed with potentially curable gastric cancer with ELM. Future research efforts focused on exploring less toxic and more efficacious treatment regimens are warranted, as is an examination of whether these patients need to undergo expanded LND, particularly after exhibiting favorable clinical responses to neoadjuvant chemotherapy.

### Lymph nodes of the pelvis

The pelvic LNs primarily include the obturator, sacral, common iliac, external iliac, and internal iliac nodes, all of which have the potential for involvement in patients with pelvic urogenital or gastrointestinal tumors. In rectal cancer patients, for example, LN staging is important in the context of disease evaluation and treatment selection, given that a high LN ratio (PNR) is associated with worse disease-free and overall survival outcomes.^183^ Rectal lymphatic drainage follows the rectal vasculature and is separated to include the superior, lateral, and inferior drainage tracts. Of these, the superior pathway is responsible for the drainage of the upper and middle rectum into the inferior mesenteric LNs, while the lateral pathway terminates at the subaortic LNs, and the inferior pathway drains the anal canal into the superficial inguinal LNs, in addition to draining the lower rectum in some cases.^184^ Proper staging generally requires the evaluation of at least 12 nodes.^185^ In the AJCC 8^th^ edition TNM staging for LNM in rectal cancer, N1 indicates 1–3 positive regional LNs with intranodal tumors ≥0.2 mm in size or the presence of any number of tumor deposits with all identifiable LNs being negative, whereas N2 indicates the presence of 4+ positive regional LNs.^186^ Total rectal mesenteric excision (TME) or tumor-specific rectal mesenteric resection with lateral pelvic LND are the standard surgical approaches to treating advanced low-grade rectal cancer. While preoperative radiotherapy can reduce the local recurrence risk for these patients, it is not associated with any significant survival benefit.^187,188^ Retrospective analyses have suggested that for low-grade rectal cancer patients, preoperative radiotherapy can achieve efficacy comparable to that of lateral LND.^189^ In line with the above, a Swedish trial performed in the 1980s found that local recurrence rates were lower for patients that underwent preoperative radiotherapy before surgery as compared to patients that underwent surgery alone.^190^ Similarly, a Dutch trial noted significant reductions in local recurrence in response to preoperative radiotherapy, and found that the combination of this approach with TME yielded even better outcomes.^187^ At present, long-term irradiation is a commonly employed therapeutic strategy that can reduce tumor burden more readily than short-term irradiation, and the combination of this approach and fluorouracil-based chemotherapy is expected to emerge as a new standard of care that can improve anal preservation rates in the near future.

In addition to TNM staging, a specific staging system for gynecologic malignancies has been established by the Federation of International of Gynecologists and Obstetricians (FIGO). This FIGO staging system strongly emphasizes the significance of LN evaluation. In cervical cancer patients, for example, the prior FIGO staging systems failed to assess LNM, resulting in the understaging of 20–40% of patients with stage IB-IIB diseases and the overstaging of 64% of stage IIIB cancers.^191^ Adequately evaluation of the abdominopelvic retroperitoneal LNs was incorporated into the 2018 revision of these FIGO staging criteria (Table 2), underscoring the importance of pretreatment CT, MRI, and PET-CT imaging evaluations, together with the pathological assessment of LNs.^192^

**Table 2:** The FIGO staging of carcinoma of the cervix uteri (2018)^192^

Stage	Description
Stage I	The carcinoma is strictly confined to the cervix (extension to the corpus should be disregarded).
IA	Invasive carcinoma that can be diagnosed only by microscopy with maximum depth of invasion ≤5 mm^a^
IA1	Measured stromal invasion ≤3 mm in depth
IA2	Measured stromal invasion >3 mm and ≤5 mm in depth
IB	Invasive carcinoma with measured deepest invasion >5 mm (greater than stage IA); lesion limited to the cervix uteri with size measured by maximum tumor diameter^b^
IB1	Invasive carcinoma >5 mm depth of stromal invasion and ≤2 cm in greatest dimension
IB2	Invasive carcinoma >2 cm and ≤4 cm in greatest dimension
IB3	Invasive carcinoma >4 cm in greatest dimension
Stage II	The cervical carcinoma invades beyond the uterus, but has not extended onto the lower third of the vagina or to the pelvic wall
IIA	Involvement limited to the upper two-thirds of the vagina without parametrial invasion
IIA1	Invasive carcinoma ≤4 cm in greatest dimension
IIA2	Invasive carcinoma >4 cm in greatest dimension
IIB	With parametrial invasion but not up to the pelvic wall
Stage III	The carcinoma involves the lower third of the vagina and/or extends to the pelvic wall and/or causes hydronephrosis or non-functioning kidney and/or involves pelvic and/or paraaortic LNs
IIIA	Carcinoma involves lower third of the vagina, with no extension to the pelvic wall
IIIB	Extension to the pelvic wall and/or hydronephrosis or non-functioning kidney (unless known to be due to another cause)
IIIC	Involvement of pelvic and/or paraaortic LNs (including micrometastases)^c^, irrespective of tumor size and extent (with r and p notations).^d^
IIIC1	Pelvic LNM only
IIIC2	Paraaortic LNM
Stage IV	The carcinoma has extended beyond the true pelvis or has involved (biopsy proven) the mucosa of the bladder or rectum. A bullous edema, as such, does not permit a case to be allotted to stage IV
IVA	Spread of the growth to adjacent organs
IVB	Spread to distant organs

^a^Imaging and pathology can be used, when available, to supplement clinical findings with respect to tumor size and extent, in all stages. Pathological findings supercede imaging and clinical findings

^b^The involvement of vascular/lymphatic spaces should not change the staging. The lateral extent of the lesion is no longer considered

^c^Isolated tumor cells do not change the stage but their presence should be recorded

^d^Adding notation of r (imaging) and p (pathology), to indicate the findings that are used to allocate the case to stage IIIC. For example, if imaging indicates pelvic lymph node metastasis, the stage allocation would be Stage IIIC1r; if confirmed by pathological findings, it would be Stage IIIC1p. The type of imaging modality or pathology technique used should always be documented. When in doubt, the lower staging should be assigned

Copyright 2019, International Federation of Gynecology and Obstetrics

### Lymph nodes of the lower extremity

Lower limb LNs are primarily distributed in the popliteal area and inguinal canal. The superficial inguinal LNs are classified into a central group and four quadrants separated by the great saphenous vein and a horizontal line at the saphenofemoral junction. Deep inguinal nodes are situated proximal to the femoral artery and vein. These inguinal LNs are a common site of metastatic progression for tumors of the external genitalia, including vulvar and penile cancers. The superior medial LNs are the most common site of lymphatic drainage for the genital area, with the superior lateral, central, and inferior medial LNs also frequently draining this area, although the same is rarely true for the inferior lateral LNs.^193^ Penile and vulvar cancer patients exhibiting inguinal LNM are diagnosed with stage III or higher disease. In penile cancer, specifically, the 5-year survival of patients with inguinal LNM but no pelvic LNM can be as high as 80%, whereas, for patients with both pelvic LNM and distant metastases, this rate falls to 0–33%. The early surgical management of non-bulky (<4 cm) LNM has been linked to significant improvements in patient survival.^194^ In penile cancer patients with high-risk disease (≥pT1G2) and clinically negative inguinal LNs, modified inguinal LND and dynamic SLNB are recommended.^195^ The comprehensive evaluation of patients with palpable inguinal LNs at diagnosis via MRI, PET-CT, and fine-needle aspiration is also warranted, given that metastatic disease will not arise in upwards of 70% of these patients.^196^ In patients exhibiting bulky or fixed inguinal LNs, it is recommended that neoadjuvant chemotherapy and subsequent consolidative surgery be provided, given that primary surgery is unlikely to be curative.^194^ Moreover, inguinal LN involvement can also occur in anal canal carcinoma and lower rectal carcinoma patients, with LNM affecting 5.9–15.1% and 2.0–4.5% of patients, respectively.^197^

The popliteal LNs are divided into superficial and deep popliteal LNs by the deep fascia.^193^ While these popliteal LNs are generally regarded as minor players in the context of lower limb LNM, they should be taken into consideration during tumor staging. Popliteal LN involvement can be observed for lower limb tumors of the distal extremities. In some patients with primary melanoma tumors situated below the knee, popliteal LN drainage may occur such that assessing the popliteal nodes can predict recurrence and overall survival, although popliteal LND does not confer any survival benefits to these patients.^152,198^ An estimated 67% of N1 patients diagnosed with rhabdomyosarcoma of lower extremities exhibit popliteal LN positivity, underscoring the importance of performing popliteal LN biopsy procedures for tumors of the lower extremities, particularly for tumors of the distal extremities.^153^

### Summary

Tumor LNM is a highly clinically significant event with direct implications for tumor staging, treatment selection, and patient prognosis. Ongoing research efforts with contribute to the more accurate and consistent classification of LNM, helping to clarify the most appropriate interventions and their associated patient outcomes. In general, patients affected by LNM tend to exhibit worse outcomes than node-negative patients. Surgery is generally used to remove local nodes harboring metastatic lesions, but the precise association between the extent of LN clearance and patient therapeutic responses warrants further evaluation. When the extent of LND is overly extensive, this may contribute to harmful complications and a reduction in overall survival. Conversely, if LND is incomplete, local recurrence may result from the remaining tumor cells, potentially contributing to even higher rates of mortality than those associated with excessive dissection. Integrated approaches that employ combinations of chemotherapy, radiotherapy, and immunotherapy are increasingly emerging as strategies for the management of primary tumor-derived LNM. Pre- or post-surgical chemotherapeutic, radiotherapeutic, and immunotherapeutic inventions can lower the risk of local LN recurrence, thereby prolonging patient survival and improving associated prognostic outcomes. Rapid biomedical advances are expected to provide an increasingly detailed understanding of the most appropriate LNM treatment strategies in the coming years.

## Diagnosis and therapeutic interventions of metastatic lymph nodes

### Methods of recognition and diagnosis

Preoperatively detecting metastases is a persistent challenge, with a wide array of imaging modalities having been tested for their utility in the context of LNM tracing, including magnetic resonance imaging (MRI), ultrasonography, computed tomography (CT), single photon emission computed tomography (SPECT)-CT, and positron emission tomography (PET)-CT.^199–206^ While PET-CT remains the gold standard imaging approach for most tumor types, all of these modalities exhibit unsatisfactory sensitivity and specificity, with CT and other traditional approaches primarily relying on metastatic LN detection based on the identification of specific morphological characteristics.^207–209^ At present, preoperative approaches that can reliably assess the extent of LND remain lacking.

**Fig. 8:**
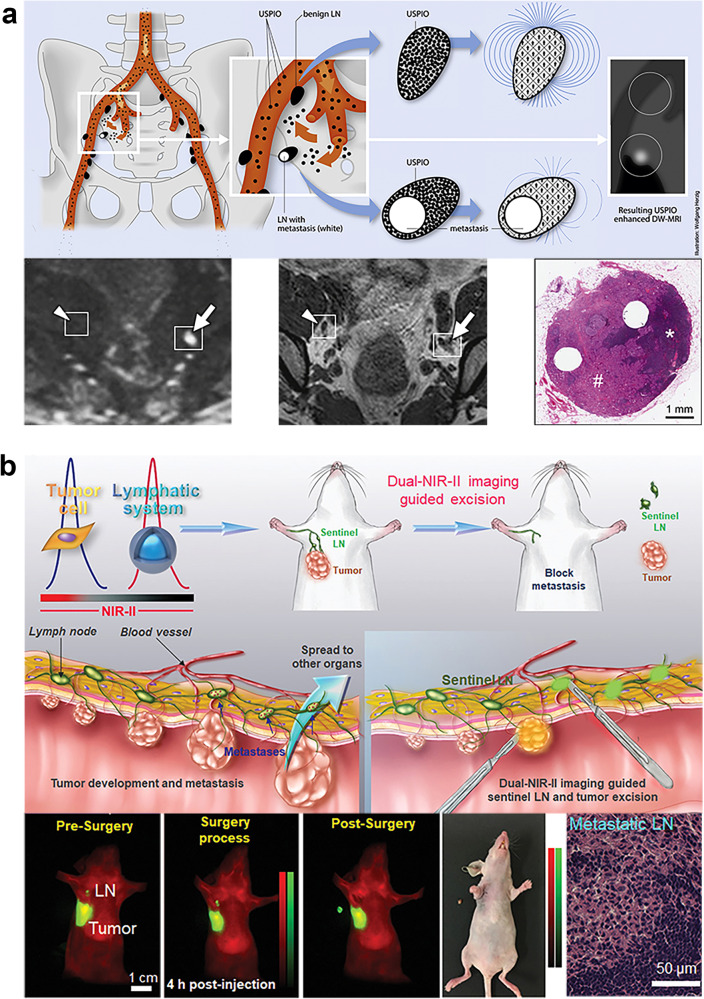
**a** Application of USPIO nanoparticles in the detection of metastases in normal-sized pelvic LNs of patients with bladder and prostate cancer. USPIO nanoparticles taken up by macrophages lead to a signal decrease on T2- or T2^*^- weighted MRI, which is lacking in the malignant LN (arrow) due to few macrophages and little USPIO nanoparticle uptake compared to the benign LN (arrowhead).^213^ Copyright 2013, European Association of Urology. **b** Application of a NIR probe in the detection of LNM in mice. The pictures present NIR imaging-guided SLN surgery in an orthotopic 4T1 breast cancer model.^217^ Copyright 2020, Wiley-VCH. USPIO, ultrasmall superparamagnetic iron oxide; LN lymph node, MRI magnetic resonance imaging, NIR near-infrared, LNM lymph node metastasis, SLN sentinel lymph node

A wide array of targeted antibody-, peptide-, nanoparticle-, and small molecule-based imaging probes have been employed in the context of LNM evaluation (Table 3). Nanoparticles, in particular, have been the focus of marked research progress in recent years.^210,211^ Nanoparticles could be promising agents for the detection of metastatic LNs as they can be readily modified and offer unique properties conducive to tumor-specific targeting and imaging enhancement.^212^ For instance, ultrasmall superparamagnetic iron oxide (USPIO) nanoparticles have commonly been tested in MRI-based studies of LNM detection^213–215^ (Fig. 8a). Metastatic LNs tend to exhibit fewer macrophages, and those macrophages that are present generally exhibit impaired phagocytic activity such that USPIO nanoparticles uptake is impaired and metastatic nodes appear brighter on T2-weighted images.^216^ Nanoparticles can also facilitate imaging using more recently developed imaging techniques, including near-infrared (NIR) fluorescence imaging (Fig. 8b), Raman mapping, photoacoustic (PA) imaging, and multimodal imaging.^217–222^

**Table 3:** Studies of targeted imaging probes for LNM detection

Author & Year	Imaging probe	Imaging modality	Targeting strategy	Tumor model
			Passive targeting	
Huang,^413^	Mesoporous silica nanoparticles labeled with ZW800, Gd^3+^, and ^64^Cu	NIR & MR & PET	Passive targeting	Breast cancer
Oh,^411^	Pluronic nanoparticles with Flamma^tm^	NIR	Passive targeting	Squamous cell carcinoma
Tseng,^414^	Lipid/calcium/phosphate nanoparticles labeled with ^111^In	SPECT	Passive targeting	Breast cancer
Partridge,^415^	Gd-lipid nanoparticles	MR	Passive targeting	Melanoma
Spaliviero,^416^	Gold-silica surface-enhanced resonance Raman spectroscopy nanoparticles	Raman	Passive targeting	Prostate cancer
Nie,^215^	Polyacrylic acid-coated USPIO	MR	Passive targeting	Squamous carcinoma
Dong,^219^	Cuttlefish melanin nanoparticle labeled with NIR dye or Gd^3+^	NIR & MR	Passive targeting	Breast cancer
			Targeting cell receptors	
Sampath,^223^	(^64^Cu-DOTA)_n_-trastuzumab-(IRDye800)_m_	NIR & PET	Trastuzumab: target HER2	Breast cancer
Tang,^417^	Aptamer–functionalized silica nanoconjugates labeled with NIR dye and ^64^Cu	NIR & PET	Aptamer: target nucleolin	Breast cancer
Hall,^221^	(^64^Cu-DOTA)_n_-Anti-EpCAM-(IRDye800)_m_	NIR & PET	Anti-EpCAM: target EpCAM	Prostate cancer
Qiao,^418^	NaGdF4:Yb,Er@NaGdF4 upconversion nanoparticles labeled with MGb2	NIR & MR	MGb_2_: target TRAK1	Gastric cancer
Atallah,^220^	AngioStam^TM^ 800	NIR	RGD: target αvβ3 integrin	Head and neck squamous cell carcinoma
Yang,^419^	Cetuximab/trastuzumab labeled with IRDye800 & Cy5.5-HA	NIR	Cetuximab: target EGFR of tumor Trastuzumab: target HER2 of tumor HA: target Lyve-1 of lymphatic endothelium	Head and neck squamous cell carcinoma & ovarian cancer
Qiu,^224^	NaGdF4:Yb,Tm,Ca@NaLuF4 core@shell upconversion nanoparticles labeled with anti-HER2 antibodies	NIR	Anti-HER2 antibodies: target HER2	Breast cancer
Shi,^227^	RGD-CuS-Cy5.5	NIR	RGD: target αvβ3 integrin	Gastric cancer
Chen,^228^	αMSH-PEG-Cy5.5-core-shell silica nanoparticles & cRGDY-PEG-CW800-core-shell silica nanoparticles labeled with ^124^I	NIR & PET	αMSH: target melanocortin-1 receptor cRGDY: target α_v_ integrins	Melanoma
Xu,^420^	Phospholipid nanoparticles core-loaded with lipiodol and a NIR dye	CT & NIR	Phospholipid: target SR-B1	Breast cancer
Liu,^421^	USPIO-PEG-sLe^X^	MR	sLe^X^: target E-selectin	Nasopharyngeal carcinoma
Tian,^217^	IR-FD & PbS/CdS core/shell quantum dots	NIR	Anti-CD3 antibody: target T cells in LNs	Breast cancer
Dai,^422^	HA- HPPS nanoparticles	NIR & PA	HA: target CD44 of tumor and Lyve-1 lymphatic endothelium HPPS: target SR-B1 of tumor	Breast cancer
Bao,^218^	FA functionalized targeted and nontargeted gap-enhanced Raman tags	Raman	FA: target folate receptor	Cervical carcinoma
			Targeting tumor microenvironment	
Cho,^238^	F127-Cy7 & Cy5.5-MMP-Q	NIR	MMP-Q: respond to MMP-2,9 of tumor microenvironment	Squamous carcinoma
Yin,^229^	^125^I-QSY21-KC(cRGD)PLGVRGY-Cy5	NIR & SPECT	KCPLGVRGY: respond to MMP-2 of tumor microenvironment cRGD: target αvβ3 integrin	Breast cancer
Bennett,^236^	UPS nanoparticles labeled with ICG	NIR	UPS micelles: respond to threshold proton concentrations	Breast cancer
Liu,^222^	Carbon nanoparticles labeled with anti-HIF-1α antibody	US & PA	Anti-HIF-1α antibody: target HIF-1α of tumor microenvironment	Breast cancer
Feng,^423^	Cy7-1/PG5-Cy5@LWHA	NIR	Cy7-1: respond to nitroreductase of tumor microenvironment LWHA: target CD44	Breast cancer
			Other strategies	
Han,^424^	Silicon nanoparticles-based exosome probes	Fluorescence	Cancer cell-derived exosomes: tumor-homing effect	Breast cancer

*NIR* near infrared, *MR* magnetic resonance, *US* ultrasound, *PA* photoacoustic, *CT* computed tomography, *PET* positron emission tomography, *SPECT* single photon emission computed tomography, *LN* lymph node, *USPIO* ultrasmall superparamagnetic iron oxide, *HA* hyaluronic acid, *MSH* melanocyte-stimulating hormone, *MMP* matrix metalloproteinase, *sLeX* sialyl Lewis X, *HPPS* high-density lipoprotein-mimicking peptide–phospholipid scaffold, *UPS* ultra-pH-sensitive, *FA* folic acid, *LWHA* low molecular weight hyaluronic acid

Several tumor-specific antigen-based targeting strategies have been developed for LNM detection to date. For example, the surface modification of certain probes with HER2-specific antibodies can endow them with a high degree of tumor-binding specificity.^223,224^ Prostate-specific membrane antigen (PSMA)-based PET-CT has been shown to offer the greatest degree of diagnostic utility for LNM imaging in patients with prostate cancer, given the expression of markedly high PSMA levels by a majority of prostate cancer cells.^225^ The RGD (Arg-Gly-Asp) peptide, which specifically engages in high-affinity interactions with the αvβ3 integrin receptor commonly overexpressed by cancer cells, can also enable efficient tumor cell targeting.^226^ RGD-decorated NPs have shown promise as tools for LNM detection.^220,227–229^ Folate receptor (FR)-α, which is heavily upregulated in many cancers derived from epithelial cells, has also been advanced as an attractive target for cancer-specific targeting that has been applied to the modification of NPs in tumor and LNM detection-focused research efforts.^218,230–232^ The efficacy of folic acid (FA)-modified nanomedicines, however, appears to be limited by FA-associated increases in IgM absorption to the surface of the prepared liposomes such that they are rapidly removed from systemic circulation and internalized by macrophages within the liver, spleen, and tumor.^233^ Macrophage-containing LNs also exhibit detectable FR-β expression, potentially resulting in a false-positive nodal signal when utilizing FR-targeting NPs in a clinical setting.^230,234^ Other targets with less cross-reactivity or the combination of multiple imaging agents and targets of interest may thus represent promising approaches to enabling more reliable imaging-based evaluation of LNM in the future.^232^

Metastatic LNs also harbor a unique tumor-associated microenvironment with changes in pH levels, proteinase activity, redox potential, and reactive nitrogen and oxygen species production that can be leveraged for tumor-targeted delivery efforts.^235^ Bennet et al.,^236^ for example, generated indocyanine green-conjugated ultra-pH sensitive (UPS) NPs capable of amplifying NIR signals in response to pH changes within the local tumor microenvironment (TME). These UPS NPs can successfully discriminate between metastatic and benign LNs. Liu et al.^222^ successfully achieved the specific delivery of drugs to metastatic LNs via the targeting of the hypoxic TME. Matrix metalloproteinases 2/9 (MMP-2/9) are proteases that are commonly active within the TME, wherein they facilitate angiogenesis and metastatic progression.^237^ MMP-2/9-responsive nanoprobes have similarly shown great promise when employed in studies of LNM.^229,238^

### Surgical treatment: balance of the extent of lymph node dissection

**Fig. 9:**
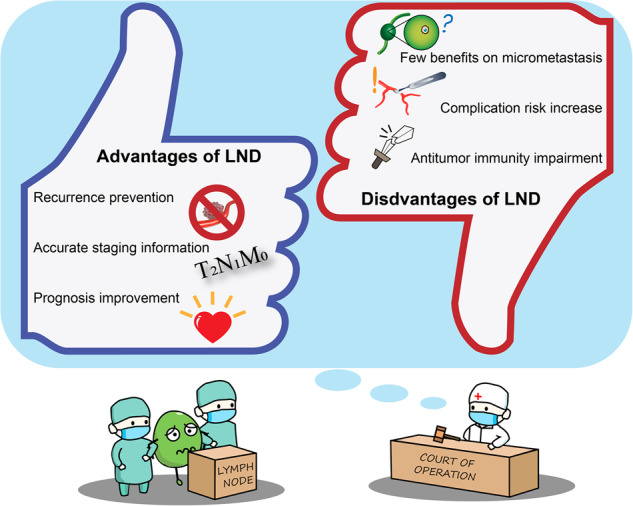
Overview diagram of the advantages and disadvantages of LND. LND lymph node dissection

LND has been firmly established as a core component of the surgical treatment of many cancer types.^239^ LND can not only eliminate tumor lesions in TDLNs to prevent recurrence, but also provide accurate tumor staging information for further treatments.^240–243^ For these reasons, LND is generally believed to improve prognostic outcomes and patient survival.^244^ Nevertheless, with research development and technical advancement in diagnosis and surgery, the guidelines for LN management have been constantly discussed and modified. Researchers have claimed to restrict the extent of LND and prevent unnecessary LND because removal of unnecessary LNs did not refine the prognosis; instead, it increased the incidence of complications that are detrimental to patients’ quality of life.^245^ In particular, the impact of micrometastasis in LNs on survival and the benefits of LND for prognosis improvement when micrometastasis is found by biopsy remain controversial.^246,247^ In addition, LND may cause damage to regional immune function, leading to impaired antitumor immune responses and reduced efficacy of immunotherapy.^239,248^ These LND-related advantages and disadvantages are presented in Fig. 9. In this section, we discuss arguments regarding LND strategies in various tumors and the role of LNs in antitumor immunity.

#### Antitumor immunity and LND

**Fig. 10:**
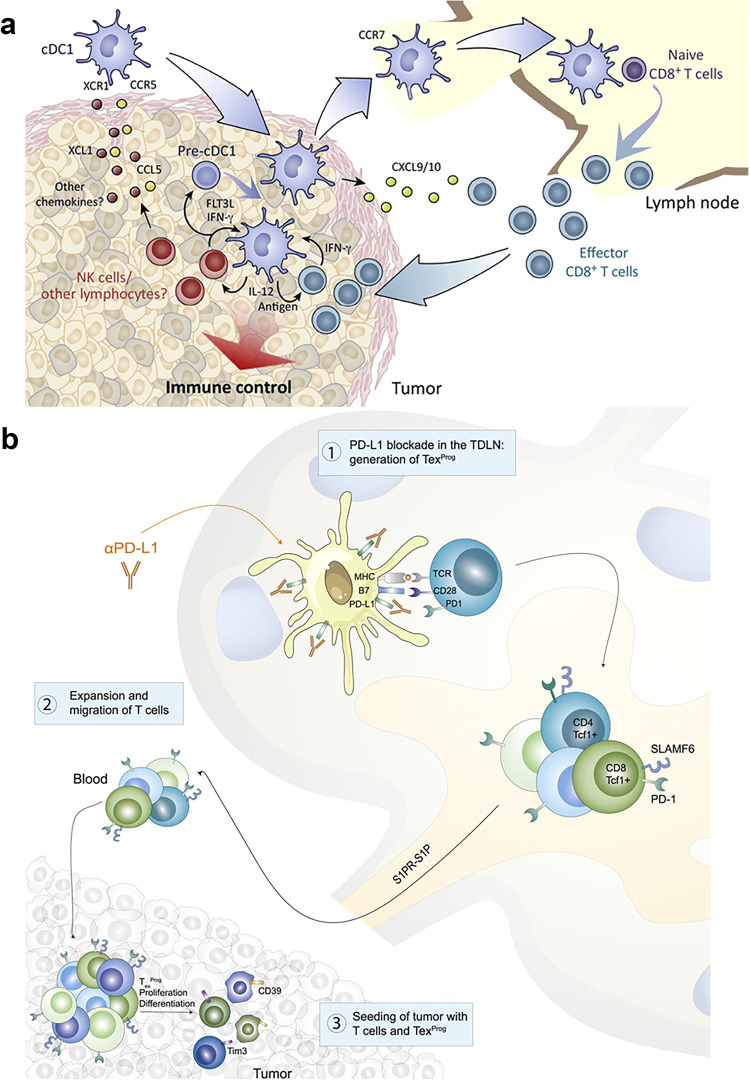
**a** cDC1s take up and transport tumor antigens to TDLNs for presentation to naïve CD8^+^ T cells, priming cytotoxic effector CD8^+^ T cells.^255^ Copyright 2018, Francis Crick Institute. **b** TDLNs are enriched for tumor-specific PD-1^+^ T cells and blocking PD-L1 in TDLNs generates progenitor-exhausted T cells that seed the tumor, which enhances antitumor immunity.^257^ Copyright 2020, Elsevier Inc. cDC1 conventional type 1 dendritic cells, TDLN tumor-draining lymph node

Given that LNs serve as secondary lymphoid organs important for the coordination of immune responses, many experts are concerned that surgical overdissection of LNs may have negative effects on antitumor immune responses. Many researchers have proposed that SLN or TDLNs are immune-suppressed and the immunosuppressive state may be present even without tumor cells, which could be enhanced by tumor invasion; further, a growing number of studies have proved their potential in antitumor immunity in support of the view that LNs must be treated cautiously.^110,249,250^ Tumor-free SLNs reportedly exhibit higher DC and T cell concentrations than tumor-bearing SLNs, suggesting that these nodes can serve as hubs for the induction of tumor-specific immune responses in the absence of direct tumor invasion.^251,252^ In a mouse model designed to simulate LND, surgical damage to the lymphatic system resulted in the progression of the established tumor as a consequence of impaired adaptive immunity.^248^ Molodtsov et al.^253^ found that tumor-specific resident memory T (Trm) cells that persist in regional LNs are key players in the prevention of metastatic disease progression. Moreover, Inamori et al.^254^ detected significant T cell repertoire overlap and no improvement in long-term prognostic outcomes following excessive LND. These results support the important role that regional LNs play in the induction of antitumor immune responses. Conventional type 1 DCs (cDC1s) can also migrate to TDLNs and prime the activation of antitumor lymphocytes present therein^255^ (Fig. 10a). Given the ability of these cDC1s to support the maintenance of a reservoir of TCF-1^+^ CD8^+^ T cells with antitumor activity, complete TDLN removal has the potential to interfere with CD8 + T cell priming and subsequent effort responses.^107,256^

Rapid advances in immunotherapeutic techniques in recent years have led to the reconsideration of the importance of regional LNs. Immune checkpoint blockade (ICB) treatment can provide benefits both locally within the tumor microenvironment as well as systemically, indicating that peripheral T cell activation and expansion may be critical for robust ICB responses. This further highlights the potential importance of TDLNs as a site where antitumor immune responses may arise in the context of ICB treatment.^239^ Indeed, in mice, LNs have been shown to be enriched for PD-1^+^ tumor-specific progenitor T cells that can play a central role in antitumor immune responses following PD-1 blockade^257^ (Fig. 10b). Fransen et al.^258^ additionally observed higher levels of PD-1 blockade-induced immune activity in the TDLNs as compared to non-TDLNs, with TDLN resection eliminating treatment-related tumor regression as a consequence of impaired immune cell infiltration of the tumor microenvironment. Recently, Rahim et al.^259^ confirmed the central role that LNs play in shaping cancer patient response to immunotherapeutic treatment by studying CD8^+^ T cells from the primary tumors, blood, and regional LNs of head and neck squamous cell carcinoma patients. These analyses revealed that uninvolved LNs contained abundant levels of progenitor-exhausted CD8^+^ T cells (Tpex), which are vital for endogenous and ICB-mediated CD8^+^ T cell responses, and these cells were clonally related to terminally exhausted cells identified within tumors. Following PD-L1 blockade treatment, Tpex and intermediate-exhausted CD8^+^ T cells (Tex-int), both of which are relevant ICB targets, were found to be localized proximal to DCs in uiLNs, with responses coinciding with an increase in circulating Tex-int. While anti-PD-L1 therapy-associated Tpex and Tex-int responses in metastatic LNs were abnormal, even metastatic LN patients exhibited limited increases in circulating post-treatment CD8^+^ T cell responses. While these above studies suggest the importance of limiting the extent of LND, additional clinical trial-derived evidence will be essential to support the evidence-based revision of guidelines for LN management.

#### Controversy regarding LND in various tumors

As a crucial part of surgery in oncology, the strategies of LND have been developing with the improvement of tumor evaluation and the progression of surgical techniques during the past few decades.^260,261^ However, the guidelines for LND are still controversial and need further refinement. Although LND plays an important role in lesion elimination and recurrence control, improper LND can do harm to the survival of patients due to impaired antitumor immunity, which has been discussed above, as well as postoperative complications caused by excessive surgical procedures.^262,263^ In clinical practice, many factors may have an influence on decisions of LND, including preoperative tumor staging and the ability of patients to tolerate the operation.^240,264,265^ Disagreements also exist among regions regarding optimal LN management owing to differences in tumor incidence, medical conditions, routine medical approaches, and histories of oncology-related surgical practices.^266–268^ In this part, we illustrate the advantages and disadvantages of LND with examples of arguments in various types of tumors.

##### Thyroid cancer: is LND necessary for prophylactic central compartment LN dissection (pCND)?

Thyroid cancer rates have steadily risen over the past 30 years,^269^ with papillary thyroid carcinoma (PTC) accounting for approximately 90% of new thyroid cancer diagnoses.^270,271^ As this tumor type is prone to lymphotropic metastasis, PTC patients commonly present with LNM.^272^ An estimated 35% of PTC patients present with cN1 disease, and of those with cN0 disease, microscopically positive nodes are estimated to be present in as many as 80% of cases.^273^ The central compartment is the most common site of PTC-associated LNM, and central compartment LND is thus recommended in cN1 PTC patients.^246^ Nevertheless, the benefit of pCND for patients with cN0 disease remains controversial.

Researchers that support pCND for cN0 patients assert that it can lower rates of locoregional recurrence as it offers accurate staging-related information, in addition to guiding radioactive ^131^I ablation therapy.^274,275^ It can also reduce the odds of reoperation-related morbidity, which can entail damage to the parathyroid glands, recurrent laryngeal nerves (RLNs), parathyroid glands, and major great vessels.^276^

Despite these assertions, data from several clinical trials have also provided support for researchers that oppose the pCND treatment of cN0 patients. Relative to patients that undergo total thyroidectomy alone, those that undergo pCND face higher rates of complications.^277^ Of these complications, the most common and relevant in patients undergoing central neck dissection procedures is hypocalcemia arising as a consequence of parathyroid gland dysfunction,^273,278^ which can occur following mechanical or thermal injury, parathyroid blood supply disruption, or the unintended or intended removal of this gland.^279^ As they are small and exhibit coloration similar to that of LNs, fat, and thyroid tissue, surgeons can also face difficulty accurately identifying the parathyroid glands.^280^ The risk of RLN or superior laryngeal nerve injury should also be taken into account, particularly for surgeons that do not routinely perform these procedures.^281^

Some clinical trials have found that there is no clear evidence in support of pCND-associated reductions in recurrence or improvements in survival.^277,282–285^ In a retrospective trial focused on 695 PTC patients, Dismukes et al.^282^ observed no differences in recurrence, distant metastasis, or persistent disease outcomes over a 38-month follow-up period when comparing cN0 patients that underwent thyroidectomy and pCND to those who underwent thyroidectomy alone.

Subclinical central LNM is also of relatively minor prognostic significance. While pCND in cN0 patients can enable more accurate TNM staging, such staging does not take differences between micro- and macrometastases in LNs into consideration.^277^ Ahn et al.^286^ conducted a prospective randomized controlled trial in which they found that LNM was confirmed to be evident in 27.5% of patients that underwent pCND, with this rate being significantly higher than that for patients in the non-pCND group. Despite this difference, no structural recurrence occurred over a 46.6 ± 9.1 month follow-up period among those 14 patients with LNMs in the pCND group, 8 of whom exhibited micro-LNMs (0.02–0.2 cm) and 6 of whom exhibited small LNMs (0.2–1.0 cm). These metastatic nodes are thus regarded as low-risk (<5% risk of recurrence) in pN1 patients, suggesting that pCND is not clinically important with respect to its value as a tool for LN risk stratification. The AJCC TNM staging system has been updated accordingly, including N0 classifications for cytologically or histologically confirmed (N0a) disease or disease without supporting radiologic or clinical evidence (N0b).^287^

##### Lung cancer: the debate regarding lobe-specific systematic lymph node dissection (L-SLND)

Lung cancer is the leading cause of cancer-related mortality, with approximately 1.8 million deaths globally each year. Of these lung cancer cases, 85% are of the NSCLC subtype.^288,289^ In early-stage NSCLC patients, surgery is the standard of care approach for staging, and the NCCN guidelines recommend systematic LND (SLND), which includes the complete dissection of the hilar and mediastinal LNs, as a routine component of lung resection procedures. The most appropriate extent of mediastinal LND (MLND), however, remains a topic of controversy. L-SLND has recently emerged as an alternative to SLND, allowing clinicians to use information on the location of the primary tumor to tailor the extent of MLND based on the corresponding lymphatic pathway.^290^

Several clinical trials have affirmed the benefits of L-SLND to the perioperative recovery, recurrence risk, and survival outcomes of patients, particularly among individuals with early-stage NSCLC.^264,291–294^ Deng et al.^264^ explored lobe-specific LNM patterns in clinical stage IA peripheral NSCLC (cT1N0M0) patients with tumors ≤3 cm and presented their recommendations for L-SLND. In this study, rates of upper lobe tumor metastasis to the subcarinal (0.3%) and lower LN zones (0.3%) were very low, and no such lower mediastinal LN metastases were evident for right middle lobe tumors. No lower lobe tumors ≤2 cm metastasized to the upper LN zone. Based on these results, the authors recommended L-SLND for upper lobe tumors only in cases where upper LN zone dissection is required, while for right middle lobe tumors, upper and subcarinal LN zone dissection is necessary for L-SLND. In patients with lower lobe tumors ≤2 cm, L-SLND was only recommended in cases where subcarinal and lower LN zone dissection was required. In other cases, systematic LN sampling or SLND should be performed instead of L-SLND.

Moreover, Chen et al.^295^ developed six preoperative imaging- and intraoperative frozen pathology-based criteria for the prediction of negative nodal station status for use when planning selective LND for peripheral clinical T1N0 invasive NSCLC patients. First, MLND was deemed unwarranted in cases with a tumor consolidation ratio ≤0.5. Second, MLND was also considered unnecessary for patients an intraoperative diagnosis of lepidic-predominant adenocarcinoma. Third, inferior MLND was not considered necessary for patients with apical segment tumors. Fourth, inferior MLND was not indicated for patients with negative hilar nodes and an absence of visceral pleural invasion. Fifth, left superior segment tumor patients did not require 4 L LND if their hilar nodes were negative. Lastly, superior MLND was not required for any patients with left basal segment tumors exhibiting hilar node negativity. The authors tested these criteria in a prospective multicenter trial enrolling 720 patients, with systematic MLND being conducted in all cases to confirm the accuracy of this approach to predicting LN involvement. Strikingly, negative node status in particular mediastinal zones was accurately predicted using this approach in all cases, providing strong support for the clinical implementation of selective LND for early-stage NSCLC patients.

Despite the above evidence, some researchers posit that following metastasis, all LN zones are at risk and should be dissected irrespective of the fact that the odds of mediastinal LNM differ as a function of primary tumor location.^290^ Handa et al.^240^ reviewed 375 patients that had undergone lobectomy with lymphadenectomy for clinical T2–3 N0–1 M0 hypermetabolic NSCLC, and determined that SLND procedures harvested more metastatic nodes than L-SLND, potentially contributing to better oncological outcomes. Notably, an estimated 6% of patients in the L-SLND group may have harbored metastatic LNs not present in lobe-specific stations that would have been missed by this procedure, potentially denying ~6% of patients in clinical practice from accessing adjuvant systemic treatment. The number of examined LNs may also be related to improved survival rates as a result of a reduction in the risk of misstaging, supporting the need for SLND.^296,297^ In a clinical trial focused on early-stage NSCLC, SLND was found to be associated with better disease-free survival than L-SLND.^298^ Further large-scale systematic clinical trials are thus warranted to clarify the advantages and limitations associated with SLND and L-SLND. The ongoing large-scale prospective randomized controlled trials currently underway in China (ChiCTR2100048415) and Japan (JCOG 1413) have the potential to further guide such LND-related decision-making for NSCLC patients.^299,300^

##### Melanoma and breast cancer: decision making after positive sentinel lymph node biopsy (SLNB)

The consensus criteria for LN management have undergone many changes in recent decades owing to the advent of SLNB procedures that use dyes or radiotracers to facilitate the identification, excision, and evaluation of SLN metastases.^301^ SLNB can enable clinicians to accurately stage metastatic spread with minimal risk of complications.^302–304^ Owing to the superficial nature of these tumors, melanoma, and breast cancer patients are particularly likely to benefit from SLNB given the great amenability of these neoplasms to preoperative tracer injection.^301^

It remains a matter of controversy as to whether complete LND (CLND) should be performed in melanoma patients with positive SLNB results. This is in part because melanoma is an extremely aggressive subtype of skin cancer with a high propensity for LNM.^305,306^ Most centers routinely perform CLND in melanoma patients with at least one positive LN, despite the fact that ~80% of patients that undergo CLND do not exhibit any additional non-sentinel node (NSN) metastases.^307^ The landmark DeCOG-SLT and MSLT-II trials provided particularly important evidence for this clinical context. The DeCOG-SLT trial^247^ screened 5547 patients, of whom 1269 (23%) were included based on the identification of a positive SLN. Of these patients, 483 were randomly assigned to undergo CND or nodal observation with nodal basin ultrasonography every 3 months. No differences in recurrence rates or 3-year overall, relapse-free, or disease-free survival were observed between these groups. Given that 66% of the included patients exhibited a low SLN tumor burden (diameter ≤1 mm), the trial researchers concluded that CLND is not appropriate for melanoma patients with SLN metastases ≤1 mm. Researchers of the MSLT-II trial^308^ evaluated 1934 and 1755 patients in intention-to-treat and per-protocol analyses, respectively, and further concluded that immediate CLND did not improve melanoma-specific survival. While CLND can contribute to greater regional nodal control and provide additional prognostic insight, it does so at the cost of potential lymphedema and other forms of morbidity.

The purported actual benefits of CLND after positive SLNB vary among studies. In an analysis of 471 SLNB-positive patients, 5-year microsatellite stability (MSS) and nodal recurrence rates were improved by CLND.^309^ Another study was conducted in the Bay of Plenty District Health Board (BOPDHB) of New Zealand.^267^ A larger mean SLN metastatic deposit size was observed in 157 SLNBs as compared to the MSLT-II trial (3.53 vs 1.07/1.11 mm), highlighting a pronounced difference between these two studies. Metastatic deposits >1 mm were also more common in the BOPDHB study (54.8 vs. 33.2/34.5%), and the rate of NSN involvement on CLND was higher (23.8% vs. 11.5%). This suggests that failing to complete CLND may expose patients to a higher degree of risk.

The above results suggest that positive SLN tumor burden in melanoma patients may have an important bearing on decision-making pertaining to CLND. A few trials to date have sought to evaluate the utility of CLND for melanoma patients in whom micrometastases were detected on SLNB.^310,311^ Susok et al.^311^ studied 258 patients with micrometastases in SLNB and performed a 20-year survival analysis, and observed no significant increase in the risk of relapse or impaired MSS when comparing patients that did undergo CLND (HR: 1.3, 95% CI: 0.8–2.3) and did not undergo CLND (HR: 1.2, 95% CI: 0.8–1.9).

CLND offers independent prognostic insights not available from other sources.^312^ In the MSLT-2 and De-COG trials, serial ultrasonographic nodal exams were performed for participating patients, yet such scans remain far from routine in many areas.^267,313^ As such, practical limitations pertaining to medical resource availability confer continued prognostic and therapeutic value to CLND in many cases. Overall, additional research is warranted to more fully explore the necessity of CLND in SLNB-positive melanoma patients and the relationship between positive SLN tumor burden and CLND-related approaches and outcomes.

Regarding breast cancer, strong evidence has been provided for forgoing axillary lymph node dissection (ALND) when SLNB is positive with micrometastases.^314,315^ The DFS rates of breast cancer patients with 1 or more micrometastatic SLNs ≤2 mm in the IBCSG 23-01 trial on 10-year follow-up were 74.9% (95% CI: 70.5–79.3) and 76.8% (95% CI: 72.5–81.0) for patients that did and did not undergo ALND, respectively (HR: 0.85, 95% CI: 0.65–1.11; log-rank *p* = 0.24 and p = 0.0024, respectively, for non-inferiority).^315^ These results indicated that the omission of ALND was not inferior to ALND. As a result, ALND is not currently recommended by the NCCN in patients with micrometastasis-positive SLNs.

##### Gastric cancer and bladder cancer: the extent of LND for advanced cancer

In 2020 alone, approximately 1,000,000 patients were diagnosed with gastric cancer, while 769,000 succumbed to this disease, ranking it as the fourth deadliest cancer type globally in large part owing to the fact that the disease is often relatively advanced when first diagnosed.^288^ In patients with advanced gastric cancer, gastrectomy remains the primary treatment approach, and the optimal extent of LND in these patients is a topic of ongoing debate.^177^ Divergent opinions on this topic have emerged in Eastern and Western nations, with D1 and D2 LND procedures being the two strategies most commonly discussed in this context. D1 LND entails the dissection of all perigastric and left gastric artery LNs, as they exhibit the highest degree of metastatic risk. In contrast, D2 dissection entails the removal of all D1 LNs, nodes along the celiac axis, and nodes along the common hepatic, proper hepatic, and splenic artery other than the splenic hilar nodes.^316^

In Eastern nations, D2 LND has been the standard approach for patients undergoing gastrectomy for several decades, reportedly offering significant advantages over D1 LND with respect to long-term survival benefits.^317^ Under the established Japanese guidelines for the treatment of gastric cancer, D2 LND is indicated, whereas potential nodal involvement cannot be excluded.^316^ In contrast, guidelines in Western nations recommend D2 LND but do not mandate this procedure.^176^ This is partially attributable to the results of the phase III Medical Research Council randomized surgical trial and the Dutch Gastric Cancer Trial. In both of these trials, D2 LND was not associated with any initial survival benefits, potentially owing to very high postoperative mortality rates following D2 dissection.^318,319^ The differences in the conclusions of clinical trials conducted in Eastern and Western nations may be attributable to the greater proportion of younger patients with less abdominal fat and fewer comorbidities in the East, as these factors may simplify the D2 procedure.^320^ Additional analyses of subgroups and long-term follow-up data revealed that pancreatectomy and splenectomy were major risk factors associated with elevated D2-related morbidity rates. Among non-pancreatectomy/splenectomy patients, the OS of individuals that underwent D2 LND was significantly longer than that of those that underwent D1 LND.^319^ The 15-year follow-up results from the Italian Gastric Cancer Study Group randomized controlled trial observed no differences in procedural outcomes between D1 and D2 dissection in the overall population, yet D2 LND was associated with significant improvements in gastric cancer-related and disease-specific survival when focusing specifically on individuals with advanced resectable disease (pT >1 N+) and LNMs.^321^ These results suggested that pancreatectomy and splenectomy had adverse effects on D2 patient outcomes. Long-term clinical trial follow-up led to the suggestion by surgeons in Japan that spleen- and pancreas-preserving modified D2 LND approaches be implemented, leading to the rapid global adoption of this approach throughout the Western world.^266^ Meta-analyses have demonstrated that pancreas- and spleen-preserving therapies can contribute to improved survival outcomes and lower rates of gastric cancer-associated death among patients that had undergone D2 LND.^322,323^ Given that specialized centers are equipped to perform pancreas- and spleen-preserving D2 resection procedures, there is international consensus regarding the inclusion of D2 LND in gastrectomy procedures for medically fit advanced gastric cancer patients, with all such procedures being conducted in specialized, high-volume centers.^177^

Ongoing clinical efforts have sought to refine D1 and D2 LND protocols for patients undergoing gastrectomy. Kang et al.,^324^ for example, observed comparable long-term survival outcomes when comparing patients with ≥ pT2 or pN + gastric cancer that underwent D2 LND or D1 + LND, the latter of which omits the resection of LNs at the proximal splenic (No. 11p) and proper hepatic artery (No. 12a), highlighting the potential adequacy of D1 + LND as a treatment for advanced gastric cancer. Besides, Yu et al.^325^ also confirmed that D2 + LND was safe and effective in patients with advanced distal gastric cancer through the additional dissection of the hepatoduodenal ligament LNs along the common bile duct (No. 12b), posterior LNs along the common hepatic artery (No. 8p), LNs behind the head of the pancreas (No. 13), and LNs along the superior mesenteric vein (No. 14 v). When focusing on patients exhibiting duodenal involvement, significant improvements in 3-year DFS were observed for patients that underwent D2 + LND as compared to D2 LND. Further large-scale clinical trials are thus warranted to provide surgeons with sufficient evidence to select the most appropriate LND extent when addressing gastric cases affecting different sites or exhibiting differing degrees of invasion.

The optimal extent of LND for advanced bladder cancer patients also remains an area of active controversy. In patients with muscle-invasive bladder cancer, radical cystectomy with pelvic lymph node dissection (PLND) is the standard-of-care treatment. In these cases, LND procedures fall into three major categories: (i) standard PLND, which entails the removal of the internal iliac, presacral, obturator fossa, and external iliac LNs up to the bifurcation of the common iliac arteries; (ii) extended PLND, which entails to the removal of LNs between the aortic bifurcation and common iliac vessels proximally, the genitofemoral nerve laterally, the circumflex iliac vein distally, and the internal iliac vessels posteriorly; and (iii) super-extended PLND, which includes continued proximal dissection to the root of the inferior mesenteric artery.^326^ A meta-analysis of six comparative studies that incorporated 2824 bladder cancer patients undergoing radical cystectomy found extended PLND to improve the recurrence-free survival of patients relative to standard PLND, although no additional survival benefits were conferred by super-extended PLND. In contrast, a recently conducted randomized multicenter phase III trial found that extended LND did not offer significant advantages over standard LND with respect to patient overall, cancer-specific, or recurrence-free survival.^245^ The negative result may be related to the fact that 14% of the cohort was comprised of T1G3 patients, given that they tend to exhibit low rates of nodal positivity. However, the mean LN yield for extended LND was almost 30% higher, increasing the odds of positive LN detection. One retrospective study focused on bladder cancer patients undergoing radical cystectomy and lymphadenectomy found super-extended PLND to be associated with elevated LN yields and increased N2/N3 rates relative to standard PLND and extended PLND, but without any corresponding increases in complication rates.^327^ The identification of additional positive LNs was conducive to more precise nodal staging such that more patients could be appropriately evaluated for adjuvant systemic treatment, translating the diagnostic benefits of extended or super-extended PLND into improved therapeutic options that support the application of this approach.^328,329^

#### Prevention and treatment of complications in LND

As noted above, LND procedures can result in complications that adversely impact patient quality of life, including lymphedema (interstitial edema caused by lymphatic insufficiency), lymphocele (lymphatic-filled cystic lesion), lymphatic or chylous fistula, hematoma, and neuroparalysis. The management of the complications is also an important part of surgical therapy for patients undergoing LND.

To prevent complications after LND, the operator should be familiar with the local anatomy of regions of dissection, and avoid damage to blood vessels, lymphatics, and nerves. Also, surgeons have made great efforts to improve the surgical procedures. For example, novel LND techniques have been proposed to avoid postoperative RLN paralysis, which is the most worrying complication for thoracic surgeons when performing LND. Chen et al.^330^ presented an advanced lymphadenectomy approach in which the two-dimensional pedicled nerve flap, which includes the left RLN, LNs along the left RLN, and tracheoesophageal vessels, was exfoliated on both sides via the dorsal suspension of the esophagus and the pushing of the trachea to the ventral side, after which isolating forceps were used to separate LNs from the left RLN. This strategy enabled surgeons to reliably identify the local anatomical structures such that they were able to avoid any RLN injury. Saeki et al.^331^ reported an alternative means of preventing RLN paralysis that consisted of using scissors to cut the vessels surrounding the RLN, rather than ultrasonic coagulating devices or similar equipment, followed by the hemostatic application of mini-clips before the vessels were cut. Otsuka et al.^332^ similarly developed what they termed a “native tissue preservation” technique aimed at lowering the odds of RLN paralysis by preserving the native tissue layer surrounding this nerve during LND and thereby avoiding the traction and bending of the left RLN.

Accurately visualizing regional lymphatic structures can also lower the risk of LND-related complications. In breast cancer, axillary reverse mapping (ARM) can benefit patients undergoing SLNB or ALND by injecting a blue dye, radioisotope, or fluorescent agent that allows for differentiation between the lymphatic channels of the breast and those of the upper arm. By allowing for the preservation of upper extremity lymphatic drainage, ARM can lower the incidence of arm lymphedema.^333^ ARM has also been confirmed to be safe in cN0 patients with positive SLNs.^334,335^

Many different medical materials have been explored as tools to help mitigate the procedural complications of LND. Fibrin glues are commonly used in surgical settings to promote tissue adherence and hemostasis, allowing for reductions in seroma magnitude, duration, and necessary evacuative punctures following SLNB or ALND.^336,337^ Applying fibrin glue in this setting, however, remains a matter of some controversy. Conversano et al.^338^ noted no reduction in postoperative seroma formation in breast cancer patients undergoing ALND following the application of a low-thrombin fibrin sealant glue. Even so, this glue was able to support ALND without wound drainage and to reduce the duration of postoperative hospitalization. Researchers have also tested the use of a gelatin-thrombin matrix in gynecologic cancer patients, revealing its ability to decrease pelvic lymphocele incidence.^339^

When they do arise, the complications resulting from LND must be treated in a timely fashion. For patients suffering from lymphedema, combined decongestive therapy (CDT) is the accepted standard of care supportive therapy, consisting of manual lymphatic drainage, gradient compression bandaging, therapeutic exercises, and skin care that allows for the conservative and surgical management of this condition as appropriate.^340^ Microsurgical lymphatic-venous anastomoses (LVA) also provide an opportunity to treat lymphedema cases that respond poorly to CDT via the reconstruction of the lymphatic vasculature.^341^ Lymphocele and lymphatic or chylous fistulae are primarily treated through percutaneous drainage and the injection of povidone-iodine, alcohol, or bleomycin as sclerosing agents.^342^ Surgical approaches for affected patients include marsupialization, which can be conducted in instances of clinically symptomatic lymphocele or cases that fail to respond to percutaneous drainage and sclerosis.^343^ Conservative means are usually sufficient to manage lymphatic or chylous fistulae, but surgery is required if leakage persists.^344,345^ Nonresolving neuroparalysis can benefit from surgical interventions aimed at promoting functional recovery, including arytenoid adduction with Type I thyroplasty in individuals suffering from RLN paralysis.^346^

#### Future perspectives of LND

In summary, even if LND has been established as a standard surgical treatment that can improve prognostic outcomes for patients, a range of issues related to this procedure warrant further research and discussion. These include:

1. The need for further discussion regarding the indications for and extent of LND. Besides, as the resection extent of primary tumor has become more and more diverse in different tumor conditions for all types of tumors, the guidelines for the extent of LND seem to be not specific enough to adapt to these different conditions.
2. The requirement for additional evidence regarding the relationship between excessive LND and immune function, with a further focus on the associated impact on immunotherapeutic efficacy.
3. The need for improved surgical techniques. While there have been marked improvements in surgical approaches in recent years, LND remains a highly precise procedure that requires the excision of LNs located in close proximity to particular nerves, blood vessels, and lymphatic structures, often requiring a prolonged operative duration and causing substantial surgical trauma.
4. The suboptimal sensitivity of current preoperative LNM detection techniques and the lack of a reliable approach to determining the extent of LND.

Researchers can seek to address the abovementioned issues through several approaches, including the following:

1. Conducting additional systematic large-scale clinical trials focused on the extent of LND, which have the potential to improve guidelines pertaining to LND and to ensure that they are better tailored to the degree of tumor invasion.
2. Performing further basic research and clinical trials exploring the impact of LND on antitumor immunity and immunotherapy efficacy, highlighting trade-offs between LN preservation and resection while guiding the design of combined surgical and immunotherapeutic interventional strategies.
3. Developing more efficient and less traumatic LND surgical procedures. For particularly difficult procedures, the extent of LND can be tailored to minimize procedure-related damage to the health of the patient, particularly for low-volume centers.
4. Further studying approaches to LNM diagnosis, as through the design of specific PET-CT probes and the clinical application of nanoparticles that can provide superior preoperative staging information such that an optimal LND strategy can be selected.

### Medical treatment: exploration of therapeutic targets

**Fig. 11:**
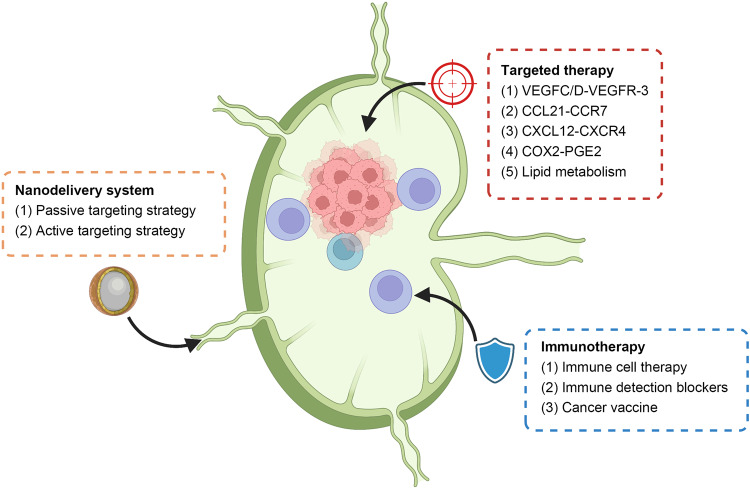
Exploration of medical treatment strategies for lymph node metastasis. Created with BioRender.com

While a combination of radiotherapy and chemotherapy has traditionally been employed to treat lymphatic metastases, recent advances in immunotherapies, targeted treatment regimens, and nanodelivery systems have increasingly provided patients with the opportunity for precision medicine-based treatment (Fig. 11). New antitumor drugs can suppress both tumor growth and metastatic progression in many cases.^27,347^

#### Targeted therapy

As lymphangiogenesis and LNM are orchestrated by many molecules and pathways, there are many hypothetical targets for clinical efforts to prevent or abrogate LNM. These include the VEGF-C/D-VEGFR-3, CCL21-CCR7, CXCL12-CXCR4, and COX-2-PGE2 signaling pathways, as well as the lipid metabolism pathway.

##### VEGF-C/D-VEGFR-3

The lymphatic vessels form a channel through which tumor cells can spread, while also enabling the active recruitment of malignant cells to LNs and regulating immune activity. Growth factors released from tumors, including VEGF-C, can promote lymphangiogenesis and anterior LN drainage in the primary tumor, thus inducing LNM.^348^ Many different VEGF-C and VEGF-D/VEGFR-3 pathway-targeting drugs have been developed and demonstrated to offer efficacy as inhibitors of solid tumor LNM.^37^ Multikinase inhibitors such as Sunitinib, Sorafenib, and Pazopanib, have received approval for the treatment of various cancers including HCC, renal cell carcinoma, and gastrointestinal mesenchymal tumors.^348,349^

##### CCL21-CCR7 and CXCL12-CXCR4

The chemokine CCL21 is primarily secreted by LECs. The resultant protein includes a long C-terminal domain that can combine with glycosaminoglycans (GAGs) to affix to the cell surface or ECM,^350^ providing a signal that can be leveraged by lymphocytes for homing to secondary lymphoid organs and the subsequent regulation of metastatic tumor development.^351^ The G protein-coupled receptor CCR7 is the only receptor for CCL21, and it is expressed on the surface of immune cells, including B cells, T cells, and DCs. CCL21-induced CCD7 signaling regulates the lymphoid migration and LN homing of T cells, DCs, and other immune cell types.^352^

Efforts to target this CCL21-CCD7 signaling axis have included CCR7-neutralizing antibodies, CCR7 agonists, specific siRNA constructs, CCR7 traps, and CCL21 mutations aimed at suppressing the lymphatic migration and invasion of tumor cells. Using a retroviral vector to overexpress CCR7 in B16 cells that were subsequently injected into murine footpads, Wiley et al.^353^ observed enhanced CCR7-B16 cell migration to regional LNs at early and late time points (1 and 3 weeks) relative to vector control, while the use of a CCL21-neutralizing antibody was sufficient to interfere with CCR7-mediated metastatic progression. This suggests that the expression of CCD7 alone can enhance B16 cell metastasis to TDLNs such that tumor cells are capable of coopting standard LN homing strategies to facilitate lymphatic metastasis, with the upregulation of a specific chemokine receptor being sufficient to enable metastatic progression. One phase IIa study found that combining intravenous pembrolizumab with the CXCR4 antagonist motixafortide was associated with improved chemotherapeutic outcomes in metastatic pancreatic ductal adenocarcinoma patients.^37,354^

##### COX-2-PGE2

DCs form an integral part of the tumor microenvironment owing to their ability to prime and regulate T cells.^355^ DC-regulating compounds such as PGE2, which strongly influences DC maturation and function, can have a pronounced impact on the local niche.^3,356^ Using a lung parenchymal tumor model generated through the direct injection of GFPV-transfected LLC cells in the soft tissue of the left lung, Ogawa et al.^92^ determined that at 1 to 3 days post-tumor implantation, regional LNs harbored COX-2-positive cells in the subcapsular region. Moreover, they found that COX-2 inhibitor therapy was sufficient to disrupt regional LNM in these animals. COX-2 may thus be expressed at early time points in premetastatic LNs, with the COX-2-mediated PGE2-EP3 signaling pathway ultimately contributing to a more robust LNM.

##### Lipid metabolism

Tumors are characterized by pronounced metabolic changes conducive to enhanced proliferation, survival, and immune evasion.^357^ Most notably, the oncogenic shift toward aerobic glycolysis, known as the Warburg effect, is a canonical hallmark of cancer.^121^ Through a comparative analysis of primary and LN metastatic tumors in mice, Lee et al.^97^ found that LNM is associated with a shift in tumor metabolic activity in favor of FAO. They subsequently found that the selective stimulation of FAO highlighted a potential role for accumulated bile acid-drive YAP activation, thereby driving the development of LNM. YAP or FAO inhibition thus provides a means of depriving tumor cells of access to bile acids and FAs within LNs as a source of energy, highlighting the promise of these approaches as a means of preventing or treating LNM.

#### Immunotherapy

Rather than targeting tumors directly, immunotherapeutic regimens rely on targeting the immune system in order to activate or restore appropriate antitumor defense mechanisms as a means of indirectly killing malignant cells.^358^ The development of increasingly robust immunotherapies has spurred growing interest in TDLNs as the key secondary lymphoid organs to which immune cells are recruited for the induction of antitumor immunty.^359^ Extant immunotherapy strategies include cell-based therapies, immune checkpoint inhibitors (ICIs), cancer vaccines, and oncolytic viruses. The first three of these modalities are discussed in greater length below.^360^

##### Immune cell therapy

Immune cell therapy relies on leveraging the properties of particular cells of interest through in vitro expansion under defined culture conditions and/or bioengineering. The resultant cells can directly kill target tumor cells or pathogens, enhance immune function, and promote tissue regeneration as a means of treating disease.^361^ Chimeric antigen receptor (CAR)-T cells are currently the most common form of cell-based antitumor immunotherapy. The FDA and other regulatory bodies have approved multiple CAR-T regimens for specific indications in light of the results of appropriate clinical trials. These engineered cells can engage a range of target proteins, including CD19, CD20, CD22, GPC3, and B-cell maturation antigen (BCMA).^362^ Trials using CAR-T cells targeting the B cell antigen CD19 have exhibited a high degree of efficacy against acute lymphocytic leukemia,^363–367^ chronic lymphocytic leukemia,^347,368,369^ and non-Hodgkin lymphoma.^370–375^ Meanwhile, CAR-T cells targeting BCMA have demonstrated activity in multiple myeloma.^376–379^

##### Immune detection blocker

ICIs such as those targeting the CTLA-4 and PD-1 pathways provide a means of overcoming the ability of tumor cells to suppress T cell activity, thereby restoring effective T cell-mediated tumor recognition and killing.^358^ The binding of PD-L1 to PD-1 triggers co-inhibitor signaling that inhibits the activation and function of effector T cells, instead favoring regulatory T cell differentiation and activity in a manner that suppresses adaptive immunity. Elevated cell surface PD-L1 and PD-L2 expression by tumor cells is a common strategy conducive to immune escape.^380,381^ A growing number of antibodies targeting PD-1 and PD-L1 have been developed and approved for clinical use to date. The US FDA has improved anti-PD-1 monoclonal antibodies, including Nivolumab, Pembrolizumab, Cemiplimab, Toripalimab, Cindilimab, and Camrelizumab, as well as anti-PD-L1 monoclonal antibodies including Atezolizumab, Avelumab, and Durvalumab.^382^

##### Cancer vaccine

Cancer vaccines are a form of active immunotherapy that rely on the use of tumor-specific antigens to induce a directed and robust antitumor immune response in immunized patients. These vaccines seek to engage both T and B cells to produce humoral and cellular immunity directed against target tumors, preventing oncogenic progression and tumor clearance. Despite intensive research in both academic and pharmaceutical settings, however, efforts to design cancer vaccines have been largely unsuccessful. Efforts to optimize therapeutic cancer vaccines center around both structural design and the selection of appropriate antigens.^383^ Ideal antigens are those that can direct immune cells to generate a robust adaptive response sufficient to target cancer stem cells and prevent recurrence while avoiding any off-target damage to healthy cells.^384^ Tailored vaccine design efforts seek to optimize professional antigen-presenting cell-mediated T cell activation and to engage a range of complementary mechanisms to overcome tumor-associated immunosuppression.^384–386^

#### Nanodelivery system

As drug uptake by the lymphatic system is relatively limited, drug delivery to this compartment tends to be suboptimal.^387^ Indeed, most small molecules drain primarily from interstitial spaces through blood capillaries, given that blood flow rates are 100 to 500 times faster than lymphatic flow rates, constraining drug delivery. Macromolecular constructs, however, can facilitate more targeted lymphatic drug delivery owing to the exclusion of these constructs from the blood due to their larger size, which is not a barrier to lymphatic entry.^15^ In light of this, researchers have employed a range of approaches to chemically modify drugs with nanocarrier materials aimed at enhancing drug enrichment within the lymphatic system, through strategies such as the covalent coupling of drugs with lipids, including FAs, diglycerides, or phosphoglycerides.^3,388^ Various delivery methods have also been employed for this purpose as well, such as the mucosal administration of particulate materials, parenteral or interstitial delivery of macromolecular materials, and intestinal or oral delivery of lipophilic drugs.^15^ Below, we provide a brief overview of nanomaterial-based approaches that seek to enhance drug delivery and retention in the LNs. These strategies include insoluble drug encapsulation,^389,390^ the protection of therapeutic molecules,^391^ and the modulation of nanomaterial biodistribution and circulation dynamics.^392,393^ Broadly speaking, these targeting strategies can be classified as being active or passive.^394^

##### Passive targeting strategy

Passive targeting approaches rely on the manipulation of nanomaterial properties such as size, shape, surface charge, and chemical composition in a manner aimed at ensuring the lymphatic enrichment of these modified nanostructures.^395^

##### Size

Unlike the endothelial layer that encloses blood vessels, the lymphatic endothelium consists of loosely connected LECs with an incomplete basement membrane layer. As a result, certain drugs can pass freely into the lymphatic system such that they can be captured by macrophages within LNs, providing an avenue for the targeted delivery of therapeutic agents to this compartment.^112^ Relative to larger NPs 100–200 nm in diameter, lipid diameters closer to 30 nm in size are better optimized for uptake by DCs, suggesting that they may be better able to target LNs.^396^

##### Shape

Efforts to adjust the shape of NPs have the potential to address certain limitations associated with the extent of therapeutic strategies, as the geometry of these particles can heavily impact their organ/tumor-targeting, cellular uptake, pharmacokinetic properties, and biodistribution.^397^ Flexible or non-spherical parties tend to exhibit a longer half-life in the systemic circulation. However, the optimal geometric properties necessary to engineer tumor-targeted NPs remain to be established, highlighting key avenues for future research.^398^

##### Surface charge

The interstitium has a net negative charge due to the glycosaminoglycans present therein.^399^ Small particles and neutral or negatively charged particles can be absorbed into the lymphatic vessels whereupon they can accumulate within LNs, while NPs with a positive charge are primarily restricted to uptake by DCs at the site of injection and direct transport through the lymphatic vessels.^396^

##### Chemical composition

Modifying the functional groups of drug molecules can enable them to more readily aggregate at lytic sites.^400^ PEGylation, for example, can decrease the immunogenicity and toxicity of certain drugs while enhancing their bioavailability, thus improving lymphatic exposure. When evaluating a subcutaneously administered dendrimer, Ryan et al.^401^ observed an increase in systemic bioavailability from 26% to nearly 100% following complete PEGylation. Hanson et al.^398^ employed PEG lipid NPs to encapsulate cyclic dinucleotides, enabling the redirection of the adjuvant to appropriate draining LNs and thus enhancing adjuvant efficacy, resulting in stronger polypeptide vaccine-induced CD8^+^ T cell responses and more robust antitumor immunotherapy. Cabral et al.^402^ recently demonstrated that polyethylene glycol (PEG)-based micelles containing platinum anticancer agents (DACHPt/m) can accumulate and inhibit melanoma LN metastases following intravenous delivery.

##### Active targeting strategy

Active targeting approaches rely on approaches that factor nanoparticle transport and internalization through the modification of nanoparticle surfaces using ligands capable of binding receptor proteins overexpressed by tumor cells. The primary ligands that have been employed in this context to date include LyP-1, TMTP1, and RGD.^403^

##### LyP-1

The nine amino acid cyclic LyP-1 homing peptide can bind specifically to the P32 cell surface receptor, which is overexpressed by tumor cells and tumor-associated LECs, whereas LyP-1 cannot bind normal LECs.^404^ Song et al.^405^ produced LyP-1 peptide-modified ^131^I-labeled dendrimers that exhibited good cytocompatibility. Stable 131I labeling was effectively achieved at a high degree of radiochemical purity in their study, allowing for the use of these dendrimers as a diagnostic tool in the context of SPECT imaging and as a radionuclide therapy agent capable of counteracting metastatic tumor progression in vitro and in vivo in a subcutaneous tumor model system.

##### TMTP1

The tumor-targeting peptide TMTP1 (NVVRQ) enables the specific targeting of metastatic tumors, even when they are early-stage occult metastatic foci. Through the fusion of TMPT1 with proteins or peptides with therapeutic efficacy, it can exert robust in vitro and in vivo antitumor activity.^406^ Wei et al.^407^ successfully combined this TMTP1 peptide with ICG-loaded PEG-PLGA micelles. They then established a model of SLN metastasis by BALB/c nude mice injected in the right hock using HeLa cells expressing firefly luciferase. These analyses revealed that the ICG-loaded TMTP1-PEG-PLGA micelles were able to rapidly diffuse from the injection site along lymphatic capillaries, reaching SLNs and then remaining present therein for 12 h.

##### RGD

Tumor cells primarily overexpress isoforms of αvβ3 integrin capable of interacting with the RGD motif with a cryptic CendR CendR motif, and this interaction is central to LNM progression. Researchers have taken advantage of this process to produce RGD-modified complexes that can facilitate targeted anticancer drug delivery following receptor-mediated internalization. Murphy et al.^408^ generated RGD-modified nanoparticles containing Dox (1 mg/kg) that were able to suppress pancreatic carcinoma growth and hilar LNM more readily than control preparations not conjugated to RGD.

### Theranostics: novel direction of development

Theranostic applications, which integrate both diagnostic and therapeutic tools, have emerged as a focus of growing research interest in cancer and other diseases in recent years. Theranostic advances have the potential to aid precision oncology efforts by facilitating patient selection, treatment planning, and subsequent monitoring. Progress in the theranostic spaces has been driven by the combined interdisciplinary research contributions from fields including chemistry, pharmacology, biomedicine, tissue engineering, nanotechnology, and material sciences.^409,410^

**Fig. 12:**
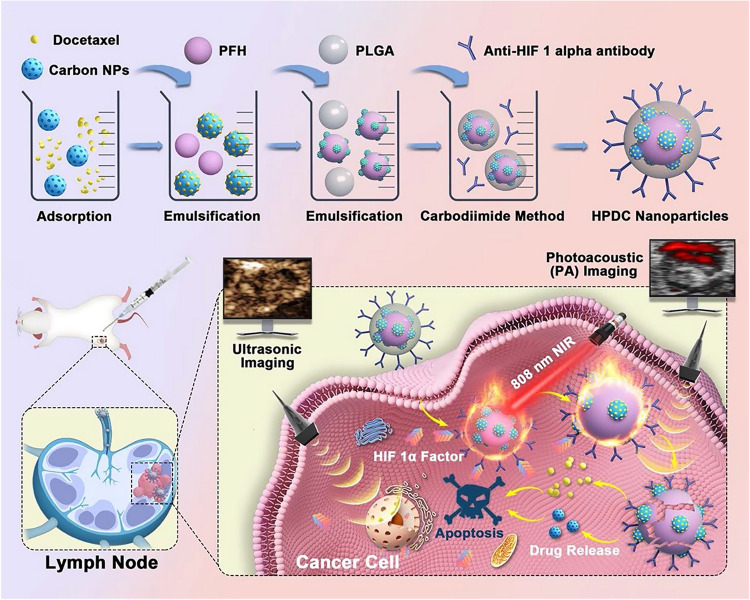
A visualized precision medicine nanoplatform of metastatic LNs for US/PA dual-modal imaging-guided in situ targeted hyperthermia-combined chemotherapy.^222^ Copyright 2021, Springer Nature. NP nanoparticle, PFH perfluorohexane, PLGA poly (lactatco-glycolic acid), LN lymph node, US ultrasonic, PA photoacoustic

Some studies have sought to apply theranostic approaches to target LNM. Oh et al.^411^ employed a combination of docetaxel-loaded Pluronic nanoparticles and the molecular imaging dye FPR-675 such that the delivery of both metastatic LNs and primary tumors would enable effective imaging and treatment. Cai et al.^412^ also prepared hybrid nanocomposite materials that were used for the multimodal imaging-guided photothermal treatment of LNM. Specifically, they utilized Fe_3_O_4_ to enable MRI imaging, ^99m^Tc as a radiotracer for SPECT imaging, and IR-1061 to facilitate photoacoustic imaging, NIR fluorescent imaging, and photothermal treatment. Moreover, Liu et al.^222^ additionally utilized carbon nanoparticles, which exhibit excellent NIR absorption and utility in the context of photoacoustic imaging-guided photothermal therapy. By loading these particles with perfluorohexane and docetaxel and mixing them with PLGA nanoparticles modified with anti-HIF-1α, they were able to achieve the US/PA dual imaging-guided and laser-triggered release of docetaxel in situ through a passive intracellular LNM targeting approach (Fig. 12).

## Conclusion

Over the last several decades, hundreds of systematic studies have explored the mechanisms that underlie the metastasis of primary tumor cells to lymph nodes, highlighting a complex array of regulatory interactions between primary tumors, disseminated tumor cells, the immune system, and the TDLN microenvironment in this context. Clinical trials focused on patients with metastatic LNs have emphasized the relevance of LNM to tumor staging, treatment planning, and prognostic outcomes. As metastatic LNs are far harder to detect than primary tumors, reliably identifying and diagnosing these metastatic nodes remains a persistent clinical problem. However, the advent of nanoparticles and other novel techniques has the potential to improve the reliability of LNM diagnosis. The number of available therapeutic targets and associated treatments undergoing testing in clinical trials also continued to expand, providing new opportunities for clinical advancement. In conclusion, the ongoing exploration of the mechanisms that govern LNM is likely to enable the identification of novel diagnostic and therapeutic strategies, ultimately contributing to the establishment of more effective LN management strategies that will improve lives and prolong the survival of countless cancer patients.

## References

[1] Gasteiger G, Ataide M, Kastenmüller W (2016). Lymph node - an organ for T-cell activation and pathogen defense. Immunol. Rev..

[2] Rezzola S, Sigmund EC, Halin C, Ronca R (2022). The lymphatic vasculature: an active and dynamic player in cancer progression. Med. Res. Rev..

[3] Zhou, H., Lei, P. J. & Padera, T. P. Progression of metastasis through lymphatic system. *Cells* **10**, 627 (2021). 10.3390/cells10030627 PMC7999434 33808959

[4] Obinu A (2018). Lymph node metastases: importance of detection and treatment strategies. Expert Opin. Drug Deliv..

[5] Takeda A, Salmi M, Jalkanen S (2023). Lymph node lymphatic endothelial cells as multifaceted gatekeepers in the immune system. Trends Immunol..

[6] Sainte-Marie G (2010). The lymph node revisited: development, morphology, functioning, and role in triggering primary immune responses. Anat. Rec..

[7] Jalkanen S, Salmi M (2020). Lymphatic endothelial cells of the lymph node. Nat. Rev. Immunol..

[8] Gillot L, Baudin L, Rouaud L, Kridelka F, Noel A (2021). The pre-metastatic niche in lymph nodes: formation and characteristics. Cell Mol. Life Sci..

[9] Li YL, Hung WC (2022). Reprogramming of sentinel lymph node microenvironment during tumor metastasis. J. Biomed. Sci..

[10] Krishnamurty AT, Turley SJ (2020). Lymph node stromal cells: cartographers of the immune system. Nat. Immunol..

[11] Girard JP, Moussion C, Förster R (2012). HEVs, lymphatics and homeostatic immune cell trafficking in lymph nodes. Nat. Rev. Immunol..

[12] Novkovic M, Onder L, Bocharov G, Ludewig B (2020). Topological structure and robustness of the lymph node conduit system. Cell Rep..

[13] Acton SE, Onder L, Novkovic M, Martinez VG, Ludewig B (2021). Communication, construction, and fluid control: lymphoid organ fibroblastic reticular cell and conduit networks. Trends Immunol..

[14] Li L, Wu J, Abdi R, Jewell CM, Bromberg JS (2021). Lymph node fibroblastic reticular cells steer immune responses. Trends Immunol..

[15] Trevaskis NL, Kaminskas LM, Porter CJ (2015). From sewer to saviour - targeting the lymphatic system to promote drug exposure and activity. Nat. Rev. Drug Discov..

[16] Follain G (2020). Fluids and their mechanics in tumour transit: shaping metastasis. Nat. Rev. Cancer.

[17] Swartz MA, Lund AW (2012). Lymphatic and interstitial flow in the tumour microenvironment: linking mechanobiology with immunity. Nat. Rev. Cancer.

[18] Jain RK, Tong RT, Munn LL (2007). Effect of vascular normalization by antiangiogenic therapy on interstitial hypertension, peritumor edema, and lymphatic metastasis: insights from a mathematical model. Cancer Res..

[19] Hompland T, Ellingsen C, Øvrebø KM, Rofstad EK (2012). Interstitial fluid pressure and associated lymph node metastasis revealed in tumors by dynamic contrast-enhanced MRI. Cancer Res..

[20] Cornelison RC, Brennan CE, Kingsmore KM, Munson JM (2018). Convective forces increase CXCR4-dependent glioblastoma cell invasion in GL261 murine model. Sci. Rep..

[21] Huang YL, Tung CK, Zheng A, Kim BJ, Wu M (2015). Interstitial flows promote amoeboid over mesenchymal motility of breast cancer cells revealed by a three dimensional microfluidic model. Integr. Biol..

[22] Shields JD (2007). Autologous chemotaxis as a mechanism of tumor cell homing to lymphatics via interstitial flow and autocrine CCR7 signaling. Cancer Cell.

[23] Li R (2018). Interstitial flow promotes macrophage polarization toward an M2 phenotype. Mol. Biol. Cell.

[24] Issa A, Le TX, Shoushtari AN, Shields JD, Swartz MA (2009). Vascular endothelial growth factor-C and C-C chemokine receptor 7 in tumor cell-lymphatic cross-talk promote invasive phenotype. Cancer Res..

[25] Das S (2013). Tumor cell entry into the lymph node is controlled by CCL1 chemokine expressed by lymph node lymphatic sinuses. J. Exp. Med..

[26] Farnsworth RH, Karnezis T, Maciburko SJ, Mueller SN, Stacker SA (2019). The interplay between lymphatic vessels and chemokines. Front. Immunol..

[27] Guan X (2015). Cancer metastases: challenges and opportunities. Acta Pharm. Sin. B.

[28] Woo HY (2022). Lung and lymph node metastases from hepatocellular carcinoma: comparison of pathological aspects. Liver Int..

[29] Huang M (2022). HSF1 facilitates the multistep process of lymphatic metastasis in bladder cancer via a novel PRMT5-WDR5-dependent transcriptional program. Cancer Commun..

[30] Wang N (2020). PRMT5/Wnt4 axis promotes lymph-node metastasis and proliferation of laryngeal carcinoma. Cell Death Dis..

[31] Yang Y (2019). The NQO1/PKLR axis promotes lymph node metastasis and breast cancer progression by modulating glycolytic reprogramming. Cancer Lett..

[32] Zhao J (2013). Mitochondrial dynamics regulates migration and invasion of breast cancer cells. Oncogene.

[33] Abe N (2016). Clinicopathological significance of lymphangiogenesis detected by immunohistochemistry using D2-40 monoclonal antibody in breast cancer. Fukushima J. Med. Sci..

[34] Mäkinen T (2001). Isolated lymphatic endothelial cells transduce growth, survival and migratory signals via the VEGF-C/D receptor VEGFR-3. EMBO J..

[35] Klein S (2016). DeepCAGE transcriptomics identify HOXD10 as a transcription factor regulating lymphatic endothelial responses to VEGF-C. J. Cell Sci..

[36] Sammarco, G. et al. Mast cells, angiogenesis and lymphangiogenesis in human gastric cancer. *Int. J. Mol. Sci.* **20**, 2106 (2019). 10.3390/ijms20092106 PMC6540185 31035644

[37] Liu S, Chen X, Lin T (2021). Lymphatic metastasis of bladder cancer: molecular mechanisms, diagnosis and targeted therapy. Cancer Lett..

[38] Zhang YQ (2019). Over-expression of both VEGF-C and Twist predicts poor prognosis in human breast cancer. Clin. Transl. Oncol..

[39] Qin T (2020). Clinical importance of VEGFC and PD-L1 co-expression in lung adenocarcinoma patients. Thorac. Cancer.

[40] Lala PK, Nandi P, Majumder M (2018). Roles of prostaglandins in tumor-associated lymphangiogenesis with special reference to breast cancer. Cancer Metastasis Rev..

[41] Sha M (2018). Expression of VEGFR-3 in intrahepatic cholangiocarcinoma correlates with unfavorable prognosis through lymphangiogenesis. Int. J. Biol. Sci..

[42] Shin JW (2006). Prox1 promotes lineage-specific expression of fibroblast growth factor (FGF) receptor-3 in lymphatic endothelium: a role for FGF signaling in lymphangiogenesis. Mol. Biol. cell.

[43] Cao R (2004). PDGF-BB induces intratumoral lymphangiogenesis and promotes lymphatic metastasis. Cancer Cell.

[44] Cadamuro M (2019). Platelet-derived growth factor-D enables liver myofibroblasts to promote tumor lymphangiogenesis in cholangiocarcinoma. J. Hepatol..

[45] Korhonen, E. A. et al. Lymphangiogenesis requires Ang2/Tie/PI3K signaling for VEGFR3 cell-surface expression. *J. Clin. Invest*. **132**, e155478 (2022). 10.1172/JCI155478 PMC9337826 35763346

[46] Cao R (2012). Collaborative interplay between FGF-2 and VEGF-C promotes lymphangiogenesis and metastasis. Proc. Natl Acad. Sci. USA.

[47] Li ZJ (2013). Insulin-like growth factor-1 induces lymphangiogenesis and facilitates lymphatic metastasis in colorectal cancer. World J. Gastroenterol..

[48] Bracher A (2013). Epidermal growth factor facilitates melanoma lymph node metastasis by influencing tumor lymphangiogenesis. J. Invest Dermatol..

[49] Kajiya K, Hirakawa S, Ma B, Drinnenberg I, Detmar M (2005). Hepatocyte growth factor promotes lymphatic vessel formation and function. EMBO J..

[50] Pak KH, Park KC, Cheong JH (2019). VEGF-C induced by TGF- beta1 signaling in gastric cancer enhances tumor-induced lymphangiogenesis. BMC Cancer.

[51] Song J (2020). CCBE1 promotes tumor lymphangiogenesis and is negatively regulated by TGFbeta signaling in colorectal cancer. Theranostics.

[52] Hong H (2016). TNF-alpha promotes lymphangiogenesis and lymphatic metastasis of gallbladder cancer through the ERK1/2/AP-1/VEGF-D pathway. BMC Cancer.

[53] Zhao G (2016). IL-6 mediates the signal pathway of JAK-STAT3-VEGF-C promoting growth, invasion and lymphangiogenesis in gastric cancer. Oncol. Rep..

[54] Al-Rawi MA, Watkins G, Mansel RE, Jiang WG (2005). Interleukin 7 upregulates vascular endothelial growth factor D in breast cancer cells and induces lymphangiogenesis in vivo. Br. J. Surg..

[55] Ming J, Zhang Q, Qiu X, Wang E (2009). Interleukin 7/interleukin 7 receptor induce c-Fos/c-Jun-dependent vascular endothelial growth factor-D up-regulation: a mechanism of lymphangiogenesis in lung cancer. Eur. J. Cancer.

[56] Chen X (2010). Role of interleukin-17 in lymphangiogenesis in non-small-cell lung cancer: Enhanced production of vascular endothelial growth factor C in non-small-cell lung carcinoma cells. Cancer Sci..

[57] Flavin R, Peluso S, Nguyen PL, Loda M (2010). Fatty acid synthase as a potential therapeutic target in cancer. Future Oncol..

[58] Bastos DC (2017). Effects of fatty acid synthase inhibitors on lymphatic vessels: an in vitro and in vivo study in a melanoma model. Lab. Invest..

[59] Du Q (2022). FASN promotes lymph node metastasis in cervical cancer via cholesterol reprogramming and lymphangiogenesis. Cell Death Dis..

[60] Kubo H (2010). Host prostaglandin EP3 receptor signaling relevant to tumor-associated lymphangiogenesis. Biomed. Pharmacother..

[61] Nagahashi M (2012). Sphingosine-1-phosphate produced by sphingosine kinase 1 promotes breast cancer progression by stimulating angiogenesis and lymphangiogenesis. Cancer Res..

[62] Weichand B (2017). S1PR1 on tumor-associated macrophages promotes lymphangiogenesis and metastasis via NLRP3/IL-1β. J. Exp. Med..

[63] Lin YC (2018). LPA(1/3) signaling mediates tumor lymphangiogenesis through promoting CRT expression in prostate cancer. Biochim. Biophys. Acta Mol. Cell Biol. Lipids.

[64] Garmy-Susini B (2010). Integrin alpha4beta1 signaling is required for lymphangiogenesis and tumor metastasis. Cancer Res..

[65] Tutunea-Fatan E, Majumder M, Xin X, Lala PK (2015). The role of CCL21/CCR7 chemokine axis in breast cancer-induced lymphangiogenesis. Mol. Cancer.

[66] Bieniasz-Krzywiec P (2019). Podoplanin-expressing macrophages promote lymphangiogenesis and lymphoinvasion in breast cancer. Cell Metab..

[67] Yan H (2017). CD146 is required for VEGF-C-induced lymphatic sprouting during lymphangiogenesis. Sci. Rep..

[68] He W (2018). Long noncoding RNA BLACAT2 promotes bladder cancer-associated lymphangiogenesis and lymphatic metastasis. J. Clin. Invest.

[69] Chen C (2020). Exosomal long noncoding RNA LNMAT2 promotes lymphatic metastasis in bladder cancer. J. Clin. Invest..

[70] Zheng S (2020). Long non-coding RNA HUMT hypomethylation promotes lymphangiogenesis and metastasis via activating FOXK1 transcription in triple-negative breast cancer. J. Hematol. Oncol..

[71] Zhu J (2021). circEHBP1 promotes lymphangiogenesis and lymphatic metastasis of bladder cancer via miR-130a-3p/TGFbetaR1/VEGF-D signaling. Mol. Ther..

[72] Kong Y (2020). circNFIB1 inhibits lymphangiogenesis and lymphatic metastasis via the miR-486-5p/PIK3R1/VEGF-C axis in pancreatic cancer. Mol. Cancer.

[73] Hood JL, San RS, Wickline SA (2011). Exosomes released by melanoma cells prepare sentinel lymph nodes for tumor metastasis. Cancer Res..

[74] Chung MK (2012). Lymphatic vessels and high endothelial venules are increased in the sentinel lymph nodes of patients with oral squamous cell carcinoma before the arrival of tumor cells. Ann. Surg. Oncol..

[75] Qian CN (2006). Preparing the "soil": the primary tumor induces vasculature reorganization in the sentinel lymph node before the arrival of metastatic cancer cells. Cancer Res..

[76] Farnsworth RH (2011). A role for bone morphogenetic protein-4 in lymph node vascular remodeling and primary tumor growth. Cancer Res..

[77] Bekkhus, T. et al. Remodeling of the lymph node high endothelial venules reflects tumor invasiveness in breast cancer and is associated with dysregulation of perivascular stromal cells. *Cancers* **13**, 211 (2021). 10.3390/cancers13020211 PMC7827313 33430113

[78] Garmy-Susini B (2013). PI3Kα activates integrin α4β1 to establish a metastatic niche in lymph nodes. Proc. Natl Acad. Sci. USA.

[79] Wei WF (2021). Periostin(+) cancer-associated fibroblasts promote lymph node metastasis by impairing the lymphatic endothelial barriers in cervical squamous cell carcinoma. Mol. Oncol..

[80] Commerford CD (2018). Mechanisms of tumor-induced lymphovascular niche formation in draining lymph nodes. Cell Rep..

[81] Paolillo, M. & Schinelli, S. Extracellular matrix alterations in metastatic processes. *Int. J. Mol. Sci.* **20**, 4947 (2019). 10.3390/ijms20194947 PMC6802000 31591367

[82] Martinez VG (2019). Fibroblastic reticular cells control conduit matrix deposition during lymph node expansion. Cell Rep..

[83] Li, L. et al. Lymph node fibroblastic reticular cells preserve a tolerogenic niche in allograft transplantation through laminin α4. *J. Clin. Invest*. **132**, e156994 (2022). 10.1172/JCI156994 PMC9246384 35775481

[84] Riedel A (2022). Tumor-derived lactic acid modulates activation and metabolic status of draining lymph node stroma. Cancer Immunol. Res..

[85] Rovera C (2022). Secretion of IL1 by dedifferentiated melanoma cells inhibits JAK1-STAT3-driven actomyosin contractility of lymph node fibroblastic reticular cells. Cancer Res..

[86] Riedel A, Shorthouse D, Haas L, Hall BA, Shields J (2016). Tumor-induced stromal reprogramming drives lymph node transformation. Nat. Immunol..

[87] Chen S (2021). Single-cell analysis reveals transcriptomic remodellings in distinct cell types that contribute to human prostate cancer progression. Nat. Cell Biol..

[88] Otto B (2014). Molecular changes in pre-metastatic lymph nodes of esophageal cancer patients. PLoS ONE.

[89] Matsuura K (2006). Maturation of dendritic cells and T-cell responses in sentinel lymph nodes from patients with breast carcinoma. Cancer.

[90] van Pul KM (2019). Selectively hampered activation of lymph node-resident dendritic cells precedes profound T cell suppression and metastatic spread in the breast cancer sentinel lymph node. J. Immunother. Cancer.

[91] Go Y (2016). Tumor-associated macrophages extend along lymphatic flow in the pre-metastatic lymph nodes of human gastric cancer. Ann. Surg. Oncol..

[92] Ogawa F (2014). Prostanoid induces premetastatic niche in regional lymph nodes. J. Clin. Invest..

[93] Kos K (2022). Tumor-educated T(regs) drive organ-specific metastasis in breast cancer by impairing NK cells in the lymph node niche. Cell Rep..

[94] Deng J (2012). S1PR1-STAT3 signaling is crucial for myeloid cell colonization at future metastatic sites. Cancer Cell.

[95] Gu Y (2019). Tumor-educated B cells selectively promote breast cancer lymph node metastasis by HSPA4-targeting IgG. Nat. Med..

[96] Coffelt SB (2015). IL-17-producing γδ T cells and neutrophils conspire to promote breast cancer metastasis. Nature.

[97] Lee CK (2019). Tumor metastasis to lymph nodes requires YAP-dependent metabolic adaptation. Science.

[98] Zhang C (2020). FABP5 promotes lymph node metastasis in cervical cancer by reprogramming fatty acid metabolism. Theranostics.

[99] Shang C (2018). LNMICC promotes nodal metastasis of cervical cancer by reprogramming fatty acid metabolism. Cancer Res..

[100] Pascual G (2017). Targeting metastasis-initiating cells through the fatty acid receptor CD36. Nature.

[101] Jia Y (2022). Long non-coding RNA NEAT1 mediated RPRD1B stability facilitates fatty acid metabolism and lymph node metastasis via c-Jun/c-Fos/SREBP1 axis in gastric cancer. J. Exp. Clin. Cancer Res..

[102] Dhatchinamoorthy K, Colbert JD, Rock KL (2021). Cancer immune evasion through loss of MHC class I antigen presentation. Front. Immunol..

[103] Axelrod ML, Cook RS, Johnson DB, Balko JM (2019). Biological consequences of MHC-II expression by tumor cells in cancer. Clin. Cancer Res..

[104] Yoshii M (2013). Association of MHC class I expression and lymph node metastasis of gastric carcinoma. Hepatogastroenterology.

[105] Messaoudene M (2019). T-cell bispecific antibodies in node-positive breast cancer: novel therapeutic avenue for MHC class I loss variants. Ann. Oncol..

[106] Park IA (2017). Expression of the MHC class II in triple-negative breast cancer is associated with tumor-infiltrating lymphocytes and interferon signaling. PLoS ONE.

[107] Reticker-Flynn NE (2022). Lymph node colonization induces tumor-immune tolerance to promote distant metastasis. Cell.

[108] Erdogdu IH (2019). MHC class 1 and PDL-1 status of primary tumor and lymph node metastatic tumor tissue in gastric cancers. Gastroenterol. Res. Pract..

[109] Kessler DJ, Mickel RA, Lichtenstein A (1988). Depressed natural killer cell activity in cervical lymph nodes containing focal metastatic squamous cell carcinoma. Arch. Otolaryngol. Head Neck Surg..

[110] Nunez NG (2020). Tumor invasion in draining lymph nodes is associated with Treg accumulation in breast cancer patients. Nat. Commun..

[111] Huang SC (2017). TGF-β1 secreted by Tregs in lymph nodes promotes breast cancer malignancy via up-regulation of IL-17RB. EMBO Mol. Med..

[112] Padera TP, Meijer EF, Munn LL (2016). The lymphatic system in disease processes and cancer progression. Annu. Rev. Biomed. Eng..

[113] Nathanson SD, Kwon D, Kapke A, Alford SH, Chitale D (2009). The role of lymph node metastasis in the systemic dissemination of breast cancer. Ann. Surg. Oncol..

[114] Quinn, J. J. et al. Single-cell lineages reveal the rates, routes, and drivers of metastasis in cancer xenografts. *Science* **371**, eabc1944 (2021). 10.1126/science.abc1944 PMC7983364 33479121

[115] Brown M (2018). Lymph node blood vessels provide exit routes for metastatic tumor cell dissemination in mice. Science.

[116] Pereira ER (2018). Lymph node metastases can invade local blood vessels, exit the node, and colonize distant organs in mice. Science.

[117] Naxerova K (2017). Origins of lymphatic and distant metastases in human colorectal cancer. Science.

[118] Leong SP (2022). The lymphatic system and sentinel lymph nodes: conduit for cancer metastasis. Clin. Exp. Metastasis.

[119] Farjah F, Tanner NT (2021). Mediastinal staging for lung cancer. Chest.

[120] De Marco C, Biondi A, Ricci R (2017). N staging: the role of the pathologist. Transl. Gastroenterol. Hepatol..

[121] Hanahan D, Weinberg RA (2011). Hallmarks of cancer: the next generation. Cell.

[122] Amin MB (2017). The eighth edition AJCC cancer staging manual: continuing to build a bridge from a population-based to a more "personalized" approach to cancer staging. CA Cancer J. Clin..

[123] Iwanaga J, Lofton C, He P, Dumont AS, Tubbs RS (2021). Lymphatic system of the head and neck. J. Craniofac. Surg..

[124] Ying M, Ahuja A (2003). Sonography of neck lymph nodes. Part I: normal lymph nodes. Clin. Radiol..

[125] Robbins KT (2002). Neck dissection classification update: revisions proposed by the American Head and Neck Society and the American Academy of Otolaryngology–Head and Neck Surgery. Arch. Otolaryngol. Head Neck Surg..

[126] Zanoni DK, Patel SG, Shah JP (2019). Changes in the 8th edition of the American Joint Committee on Cancer (AJCC) Staging of Head and Neck Cancer: rationale and implications. Curr. Oncol. Rep..

[127] Caudell JJ (2022). NCCN Guidelines® Insights: head and neck cancers, version 1.2022. J. Natl Compr. Cancer Netw..

[128] Bhattacharya P, Mukherjee R (2021). Lymph node extracapsular extension as a marker of aggressive phenotype: classification, prognosis and associated molecular biomarkers. Eur. J. Surg. Oncol..

[129] Adler C, Lubner MG, Menias CO, Lubner SJ, Dahiya N (2022). What’s in a node? The clinical and radiologic significance of Virchow’s node. Abdom. Radiol..

[130] Chow LQM (2020). Head and neck cancer. N. Engl. J. Med..

[131] Robbins KT (2008). Consensus statement on the classification and terminology of neck dissection. Arch. Otolaryngol. Head. Neck Surg..

[132] Giammarile F (2022). Sentinel lymph node methods in breast cancer. Semin. Nucl. Med..

[133] Jana S, Muscarella RA, Jones D (2021). The multifaceted effects of breast cancer on tumor-draining lymph nodes. Am. J. Pathol..

[134] Teichgraeber DC, Guirguis MS, Whitman GJ (2021). Breast cancer staging: updates in the AJCC Cancer Staging Manual, 8th Edition, and current challenges for radiologists, from the AJR special series on cancer staging. Am. J. Roentgenol..

[135] Sun SX, Moseley TW, Kuerer HM, Yang WT (2020). Imaging-based approach to axillary lymph node staging and sentinel lymph node biopsy in patients with breast cancer. Am. J. Roentgenol..

[136] Qiu SQ (2018). Evolution in sentinel lymph node biopsy in breast cancer. Crit. Rev. Oncol. Hematol..

[137] Olson JA (2008). Impact of immediate versus delayed axillary node dissection on surgical outcomes in breast cancer patients with positive sentinel nodes: results from American College of Surgeons Oncology Group Trials Z0010 and Z0011. J. Clin. Oncol..

[138] Galimberti V (2013). Axillary dissection versus no axillary dissection in patients with sentinel-node micrometastases (IBCSG 23-01): a phase 3 randomised controlled trial. Lancet Oncol..

[139] Krag DN (2010). Sentinel-lymph-node resection compared with conventional axillary-lymph-node dissection in clinically node-negative patients with breast cancer: overall survival findings from the NSABP B-32 randomised phase 3 trial. Lancet Oncol..

[140] Dominick SA, Natarajan L, Pierce JP, Madanat H, Madlensky L (2014). The psychosocial impact of lymphedema-related distress among breast cancer survivors in the WHEL Study. Psychooncology.

[141] Gentilini O, Veronesi U (2012). Abandoning sentinel lymph node biopsy in early breast cancer? A new trial in progress at the European Institute of Oncology of Milan (SOUND: Sentinel node vs Observation after axillary UltraSouND). Breast.

[142] Donker M (2014). Radiotherapy or surgery of the axilla after a positive sentinel node in breast cancer (EORTC 10981-22023 AMAROS): a randomised, multicentre, open-label, phase 3 non-inferiority trial. Lancet Oncol..

[143] Donker M (2013). Comparison of the sentinel node procedure between patients with multifocal and unifocal breast cancer in the EORTC 10981-22023 AMAROS Trial: identification rate and nodal outcome. Eur. J. Cancer.

[144] Overgaard M (1997). Postoperative radiotherapy in high-risk premenopausal women with breast cancer who receive adjuvant chemotherapy. Danish Breast Cancer Cooperative Group 82b Trial. N. Engl. J. Med..

[145] Ragaz J (1997). Adjuvant radiotherapy and chemotherapy in node-positive premenopausal women with breast cancer. N. Engl. J. Med..

[146] Fisher B (1997). Effect of preoperative chemotherapy on local-regional disease in women with operable breast cancer: findings from National Surgical Adjuvant Breast and Bowel Project B-18. J. Clin. Oncol..

[147] van der Hage JA (2001). Preoperative chemotherapy in primary operable breast cancer: results from the European Organization for Research and Treatment of Cancer trial 10902. J. Clin. Oncol..

[148] Bear HD (2003). The effect on tumor response of adding sequential preoperative docetaxel to preoperative doxorubicin and cyclophosphamide: preliminary results from National Surgical Adjuvant Breast and Bowel Project Protocol B-27. J. Clin. Oncol..

[149] Golshan M (2015). Impact of neoadjuvant chemotherapy in stage II-III triple negative breast cancer on eligibility for breast-conserving surgery and breast conservation rates: surgical results from CALGB 40603 (Alliance). Ann. Surg..

[150] Hunt KK (2009). Sentinel lymph node surgery after neoadjuvant chemotherapy is accurate and reduces the need for axillary dissection in breast cancer patients. Ann. Surg..

[151] Poortmans PM (2020). Internal mammary and medial supraclavicular lymph node chain irradiation in stage I-III breast cancer (EORTC 22922/10925): 15-year results of a randomised, phase 3 trial. Lancet Oncol..

[152] Jinnai S, Namikawa K, Takahashi A, Ogata D, Yamazaki N (2022). Incidence and patterns of lymphatic drainage to the epitrochlear and popliteal sentinel lymph nodes in malignant melanoma of the distal extremities: a single-institution retrospective study. Int. J. Dermatol..

[153] Terwisscha van Scheltinga CEJ (2022). In transit metastases in children, adolescents and young adults with localized rhabdomyosarcoma of the distal extremities: analysis of the EpSSG RMS 2005 study. Eur. J. Surg. Oncol..

[154] Zhou Y (2021). Case report: intercostal lymph node metastasis: a case report and review of the literature. Front. Oncol..

[155] Friedberg JS (2020). Posterior intercostal lymph nodes double recurrence and death risk in malignant pleural mesothelioma. Ann. Thorac. Surg..

[156] Berger I (2021). CT for detection of malignant posterior intercostal lymph nodes in patients undergoing pre-operative staging for malignant pleural mesothelioma. Lung Cancer.

[157] Shamji FM, Beauchamp G, Sekhon HJS (2021). The lymphatic spread of lung cancer: an investigation of the anatomy of the lymphatic drainage of the lungs and preoperative mediastinal staging. Thorac. Surg. Clin..

[158] Asamura H (2015). The International Association for the Study of Lung Cancer Lung Cancer Staging Project: proposals for the revision of the N descriptors in the forthcoming 8th edition of the TNM classification for lung cancer. J. Thorac. Oncol..

[159] Fu Z (2023). Excellent survival of pathological N0 small cell lung cancer patients following surgery. Eur. J. Med. Res..

[160] Rusch VW (2009). The IASLC lung cancer staging project: a proposal for a new international lymph node map in the forthcoming seventh edition of the TNM classification for lung cancer. J. Thorac. Oncol..

[161] Yun JK (2019). Comparison between lymph node station- and zone-based classification for the future revision of node descriptors proposed by the International Association for the Study of Lung Cancer in surgically resected patients with non-small-cell lung cancer. Eur. J. Cardiothorac. Surg..

[162] Rena O (2014). Metastasis to subsegmental and segmental lymph nodes in patients resected for non-small cell lung cancer: prognostic impact. Ann. Thorac. Surg..

[163] Osarogiagbon, R. U. et al. The International Association for the Study of Lung Cancer Lung Cancer Staging Project: overview of challenges and opportunities in revising the nodal classification of lung cancer. *J. Thorac. Oncol.* **18**, 410–418 (2022). 10.1016/j.jtho.2022.12.009 PMC10065917 36572339

[164] Howington JA, Blum MG, Chang AC, Balekian AA, Murthy SC (2013). Treatment of stage I and II non-small cell lung cancer: diagnosis and management of lung cancer, 3rd ed: American College of Chest Physicians evidence-based clinical practice guidelines. Chest.

[165] Ettinger DS (2021). NCCN guidelines insights: non-small cell lung cancer, version 2.2021. J. Natl Compr. Cancer Netw..

[166] Toubat O (2020). Disparities in guideline-concordant treatment for pathologic N1 non-small cell lung cancer. Ann. Thorac. Surg..

[167] Cerfolio, R. J. & Bryant, A. S. Survival of patients with unsuspected N2 (stage IIIA) nonsmall-cell lung cancer. *Ann. Thorac. Surg*. 86, 362–366 (2008). 10.1016/j.athoracsur.2008.04.042 18640297

[168] Miller M, Hanna N (2021). Advances in systemic therapy for non-small cell lung cancer. BMJ.

[169] Cho HS, Ahn JH (2022). Nomenclature and lymphatic drainage patterns of abdominal lymph nodes. J. Korean Soc. Radiol..

[170] Lehnert T, Erlandson RA, Decosse JJ (1985). Lymph and blood capillaries of the human gastric mucosa. A morphologic basis for metastasis in early gastric carcinoma. Gastroenterology.

[171] Alexander JS, Ganta VC, Jordan PA, Witte MH (2010). Gastrointestinal lymphatics in health and disease. Pathophysiology.

[172] Katai H (2018). Five-year survival analysis of surgically resected gastric cancer cases in Japan: a retrospective analysis of more than 100,000 patients from the nationwide registry of the Japanese Gastric Cancer Association (2001–2007). Gastric Cancer.

[173] Kinami S (2019). Precision surgical approach with lymph-node dissection in early gastric cancer. World J. Gastroenterol..

[174] Koufuji K, Takeda J, Hashimoto K, Tanaka T, Kakegawa T (1991). Early gastric cancer associated with synchronous liver metastasis. Kurum. Med. J..

[175] Japanese Gastric Cancer Association. Japanese classification of gastric carcinoma: 3rd English edition. *Gastric Cancer* **14**, 101–112 (2011). 10.1007/s101209800016 11957040

[176] Ajani JA (2022). Gastric cancer, version 2.2022, NCCN clinical practice guidelines in oncology. J. Natl Compr. Cancer Netw..

[177] Smyth EC, Nilsson M, Grabsch HI, van Grieken NC, Lordick F (2020). Gastric cancer. Lancet.

[178] Li GZ, Doherty GM, Wang J (2022). Surgical management of gastric cancer: a review. JAMA Surg..

[179] Sasako M (2008). D2 lymphadenectomy alone or with para-aortic nodal dissection for gastric cancer. N. Engl. J. Med..

[180] Yoshikawa T (2009). Phase II study of neoadjuvant chemotherapy and extended surgery for locally advanced gastric cancer. Br. J. Surg..

[181] Tsuburaya A (2014). Neoadjuvant chemotherapy with S-1 and cisplatin followed by D2 gastrectomy with para-aortic lymph node dissection for gastric cancer with extensive lymph node metastasis. Br. J. Surg..

[182] Takahari D (2020). Long-term outcomes of preoperative docetaxel with cisplatin plus S-1 therapy for gastric cancer with extensive nodal metastasis (JCOG1002). Gastric Cancer.

[183] Jin C (2018). Lymph node ratio is an independent prognostic factor for rectal cancer after neoadjuvant therapy: a meta-analysis. J. Evid. Based Med..

[184] Bates DDB (2022). MRI for rectal cancer: staging, mrCRM, EMVI, lymph node staging and post-treatment response. Clin. Colorectal Cancer.

[185] Kim HJ, Choi GS (2019). Clinical implications of lymph node metastasis in colorectal cancer: current status and future perspectives. Ann. Coloproctol..

[186] Benson AB (2022). Rectal cancer, version 2.2022, NCCN clinical practice guidelines in oncology. J. Natl Compr. Cancer Netw..

[187] Kapiteijn E (2001). Preoperative radiotherapy combined with total mesorectal excision for resectable rectal cancer. N. Engl. J. Med..

[188] Sebag-Montefiore D (2009). Preoperative radiotherapy versus selective postoperative chemoradiotherapy in patients with rectal cancer (MRC CR07 and NCIC-CTG C016): a multicentre, randomised trial. Lancet.

[189] Watanabe T (2002). Extended lymphadenectomy and preoperative radiotherapy for lower rectal cancers. Surgery.

[190] Swedish Rectal Cancer T (1997). Improved survival with preoperative radiotherapy in resectable rectal cancer. N. Engl. J. Med..

[191] Lee SI, Atri M (2019). 2018 FIGO staging system for uterine cervical cancer: enter cross-sectional imaging. Radiology.

[192] Bhatla N (2019). Revised FIGO staging for carcinoma of the cervix uteri. Int. J. Gynaecol. Obstet..

[193] Shinaoka A (2020). Lower-limb lymphatic drainage pathways and lymph nodes: a CT lymphangiography cadaver study. Radiology.

[194] Leone A, Diorio GJ, Pettaway C, Master V, Spiess PE (2017). Contemporary management of patients with penile cancer and lymph node metastasis. Nat. Rev. Urol..

[195] Clark PE (2013). Penile cancer: clinical practice guidelines in oncology. J. Natl Compr. Cancer Netw..

[196] Spiess PE (2013). Current concepts in penile cancer. J. Natl Compr. Cancer Netw..

[197] Sato H (2022). Management of inguinal lymph node metastases from rectal and anal canal adenocarcinoma. Colorectal Dis..

[198] Nijhuis AAG, de AOSFID, Uren RF, Thompson JF, Nieweg OE (2019). Clinical importance and surgical management of sentinel lymph nodes in the popliteal fossa of melanoma patients. Eur. J. Surg. Oncol..

[199] Schmid-Bindert G (2012). Predicting malignancy in mediastinal lymph nodes by endobronchial ultrasound: a new ultrasound scoring system. Respirology.

[200] Li F (2020). Using ultrasound features and radiomics analysis to predict lymph node metastasis in patients with thyroid cancer. BMC Surg..

[201] Shin SY, Hong IK, Jo YS (2019). Quantitative computed tomography texture analysis: can it improve diagnostic accuracy to differentiate malignant lymph nodes?. Cancer Imaging.

[202] Ohno Y (2004). Metastases in mediastinal and hilar lymph nodes in patients with non-small cell lung cancer: quantitative and qualitative assessment with STIR turbo spin-echo MR imaging. Radiology.

[203] Usuda K (2013). Advantages of diffusion-weighted imaging over positron emission tomography-computed tomography in assessment of hilar and mediastinal lymph node in lung cancer. Ann. Surg. Oncol..

[204] Apostolova I, Wedel F, Brenner W (2016). Imaging of tumor metabolism using positron emission tomography (PET). Recent Results Cancer Res..

[205] Hofman MS (2020). Prostate-specific membrane antigen PET-CT in patients with high-risk prostate cancer before curative-intent surgery or radiotherapy (proPSMA): a prospective, randomised, multicentre study. Lancet.

[206] Paydary K (2019). The evolving role of FDG-PET/CT in the diagnosis, staging, and treatment of breast cancer. Mol. Imaging Biol..

[207] Marino MA, Avendano D, Zapata P, Riedl CC, Pinker K (2020). Lymph node imaging in patients with primary breast cancer: concurrent diagnostic tools. Oncologist.

[208] Kikano EG (2021). PET/CT variants and pitfalls in breast cancers. Semin. Nucl. Med..

[209] Wilkinson, M. J. et al. CT diagnosis of ilioinguinal lymph node metastases in melanoma using radiological characteristics beyond size and asymmetry. *BJS Open* **5**, zraa005 (2021). 10.1093/bjsopen/zraa005 PMC7893466 33609385

[210] Hsu JC (2020). Nanoparticle contrast agents for X-ray imaging applications. Wiley Interdiscip. Rev. Nanomed. Nanobiotechnol..

[211] Ji H (2022). Lanthanide-based metal-organic frameworks solidified by gelatin-methacryloyl hydrogels for improving the accuracy of localization and excision of small pulmonary nodules. J. Nanobiotechnol..

[212] Han X, Xu K, Taratula O, Farsad K (2019). Applications of nanoparticles in biomedical imaging. Nanoscale.

[213] Birkhauser FD (2013). Combined ultrasmall superparamagnetic particles of iron oxide-enhanced and diffusion-weighted magnetic resonance imaging facilitates detection of metastases in normal-sized pelvic lymph nodes of patients with bladder and prostate cancer. Eur. Urol..

[214] Vallabani NVS, Singh S, Karakoti AS (2019). Magnetic nanoparticles: current trends and future aspects in diagnostics and nanomedicine. Curr. Drug Metab..

[215] Nie Y (2020). A stable USPIO capable for MR lymphography with ultra-low effective dosage. Nanomedicine.

[216] Dadfar SM (2019). Iron oxide nanoparticles: diagnostic, therapeutic and theranostic applications. Adv. Drug Deliv. Rev..

[217] Tian R (2020). Multiplexed NIR-II probes for lymph node-invaded cancer detection and imaging-guided surgery. Adv. Mater..

[218] Bao Z (2021). Ratiometric Raman nanotags enable intraoperative detection of metastatic sentinel lymph node. Biomaterials.

[219] Dong J (2022). A natural cuttlefish melanin nanoprobe for preoperative and intraoperative mapping of lymph nodes. Nanomedicine.

[220] Atallah I (2015). Role of near-infrared fluorescence imaging in the resection of metastatic lymph nodes in an optimized orthotopic animal model of HNSCC. Eur. Ann. Otorhinolaryngol. Head. Neck Dis..

[221] Hall MA (2012). Imaging prostate cancer lymph node metastases with a multimodality contrast agent. Prostate.

[222] Liu W (2021). A novel targeted multifunctional nanoplatform for visual chemo-hyperthermia synergy therapy on metastatic lymph nodes via lymphatic delivery. J. Nanobiotechnol..

[223] Sampath L, Kwon S, Hall MA, Price RE, Sevick-Muraca EM (2010). Detection of cancer metastases with a dual-labeled near-infrared/positron emission tomography imaging agent. Transl. Oncol..

[224] Qiu S (2018). Detection of lymph node metastasis with near-infrared upconversion luminescent nanoprobes. Nanoscale.

[225] Farolfi A (2021). Current and emerging clinical applications of PSMA PET diagnostic imaging for prostate cancer. J. Nucl. Med..

[226] Sheikh A, Md S, Kesharwani P (2021). RGD engineered dendrimer nanotherapeutic as an emerging targeted approach in cancer therapy. J. Control Release.

[227] Shi H (2018). Tumor-targeting CuS nanoparticles for multimodal imaging and guided photothermal therapy of lymph node metastasis. Acta Biomater..

[228] Chen F (2019). Molecular phenotyping and image-guided surgical treatment of melanoma using spectrally distinct ultrasmall core-shell silica nanoparticles. Sci. Adv..

[229] Yin L (2019). Rational design and synthesis of a metalloproteinase-activatable probe for dual-modality imaging of metastatic lymph nodes in vivo. J. Org. Chem..

[230] Hoogstins CE (2016). A novel tumor-specific agent for intraoperative near-infrared fluorescence imaging: a translational study in healthy volunteers and patients with ovarian cancer. Clin. Cancer Res..

[231] Bonvin D, Bastiaansen JAM, Stuber M, Hofmann H, Mionic Ebersold M (2017). Folic acid on iron oxide nanoparticles: platform with high potential for simultaneous targeting, MRI detection and hyperthermia treatment of lymph node metastases of prostate cancer. Dalton Trans..

[232] de Jong JM, Hoogendam JP, Braat A, Zweemer RP, Gerestein CG (2021). The feasibility of folate receptor alpha- and HER2-targeted intraoperative fluorescence-guided cytoreductive surgery in women with epithelial ovarian cancer: a systematic review. Gynecol. Oncol..

[233] Wang H (2020). Interrogation of folic acid-functionalized nanomedicines: the regulatory roles of plasma proteins reexamined. ACS Nano.

[234] Randall LM, Wenham RM, Low PS, Dowdy SC, Tanyi JL (2019). A phase II, multicenter, open-label trial of OTL38 injection for the intra-operative imaging of folate receptor-alpha positive ovarian cancer. Gynecol. Oncol..

[235] Kim J, Archer PA, Thomas SN (2021). Innovations in lymph node targeting nanocarriers. Semin. Immunol..

[236] Bennett ZT (2020). Detection of lymph node metastases by ultra-pH-sensitive polymeric nanoparticles. Theranostics.

[237] Gialeli C, Theocharis AD, Karamanos NK (2011). Roles of matrix metalloproteinases in cancer progression and their pharmacological targeting. FEBS J..

[238] Cho HJ (2019). Tumor microenvironment-responsive fluorogenic nanoprobe for ratiometric dual-channel imaging of lymph node metastasis. Colloids Surf. B Biointerfaces.

[239] du Bois H, Heim TA, Lund AW (2021). Tumor-draining lymph nodes: at the crossroads of metastasis and immunity. Sci. Immunol..

[240] Handa Y (2021). Systematic versus lobe-specific mediastinal lymphadenectomy for hypermetabolic lung cancer. Ann. Surg. Oncol..

[241] Perera M (2018). Pelvic lymph node dissection during radical cystectomy for muscle-invasive bladder cancer. Nat. Rev. Urol..

[242] John NT, Blum KA, Hakimi AA (2019). Role of lymph node dissection in renal cell cancer. Urol. Oncol..

[243] Karachun A (2020). Short-term outcomes of a multicentre randomized clinical trial comparing D2 versus D3 lymph node dissection for colonic cancer (COLD trial). Br. J. Surg..

[244] Brenkman HJF (2017). A high lymph node yield is associated with prolonged survival in elderly patients undergoing curative gastrectomy for cancer: a Dutch population-based cohort study. Ann. Surg. Oncol..

[245] Gschwend JE (2019). Extended versus limited lymph node dissection in bladder cancer patients undergoing radical cystectomy: survival results from a prospective, randomized trial. Eur. Urol..

[246] Patel KN (2020). The American Association of Endocrine Surgeons Guidelines for the definitive surgical management of thyroid disease in adults. Ann. Surg..

[247] Leiter U (2016). Complete lymph node dissection versus no dissection in patients with sentinel lymph node biopsy positive melanoma (DeCOG-SLT): a multicentre, randomised, phase 3 trial. Lancet Oncol..

[248] Nakamura Y (2018). Surgical damage to the lymphatic system promotes tumor growth via impaired adaptive immune response. J. Dermatol. Sci..

[249] Grotz TE, Mansfield AS, Jakub JW, Markovic SN (2012). Regional lymphatic immunity in melanoma. Melanoma Res..

[250] Li X (2021). Immune characters and plasticity of the sentinel lymph node in colorectal cancer patients. J. Immunol. Res..

[251] Poindexter NJ, Sahin A, Hunt KK, Grimm EA (2004). Analysis of dendritic cells in tumor-free and tumor-containing sentinel lymph nodes from patients with breast cancer. Breast Cancer Res..

[252] Kohrt HE (2005). Profile of immune cells in axillary lymph nodes predicts disease-free survival in breast cancer. PLoS Med..

[253] Molodtsov AK (2021). Resident memory CD8(+) T cells in regional lymph nodes mediate immunity to metastatic melanoma. Immunity.

[254] Inamori, K. et al. Importance of lymph node immune responses in MSI-H/dMMR colorectal cancer. *JCI Insight* **6**, e137365 (2021). 10.1172/jci.insight.137365 PMC8262295 33755600

[255] Bottcher JP, Reis e Sousa C (2018). The role of type 1 conventional dendritic cells in cancer immunity. Trends Cancer.

[256] Schenkel JM (2021). Conventional type I dendritic cells maintain a reservoir of proliferative tumor-antigen specific TCF-1(+) CD8(+) T cells in tumor-draining lymph nodes. Immunity.

[257] Dammeijer F (2020). The PD-1/PD-L1-checkpoint restrains T cell immunity in tumor-draining lymph nodes. Cancer Cell.

[258] Fransen, M. F. et al. Tumor-draining lymph nodes are pivotal in PD-1/PD-L1 checkpoint therapy. *JCI Insight* **3**, e124507 (2018). 10.1172/jci.insight.124507 PMC6328025 30518694

[259] Rahim MK (2023). Dynamic CD8(+) T cell responses to cancer immunotherapy in human regional lymph nodes are disrupted in metastatic lymph nodes. Cell.

[260] Fisher CS, Margenthaler JA, Hunt KK, Schwartz T (2020). The landmark series: axillary management in breast cancer. Ann. Surg. Oncol..

[261] Qiu ML (2019). Current state of esophageal cancer surgery in China: a national database analysis. BMC Cancer.

[262] Asai S (2019). The impact of cervical lymph node dissection on acid and duodenogastroesophageal reflux after intrathoracic esophagogastrostomy following transthoracic esophagectomy. Surg. Today.

[263] Mano R, Di Natale R, Sheinfeld J (2019). Current controversies on the role of retroperitoneal lymphadenectomy for testicular cancer. Urol. Oncol..

[264] Deng HY (2020). Lobe-specific lymph node dissection for clinical early-stage (cIA) peripheral non-small cell lung cancer patients: what and how?. Ann. Surg. Oncol..

[265] Harter P (2019). A randomized trial of lymphadenectomy in patients with advanced ovarian neoplasms. N. Engl. J. Med..

[266] Faiz Z, Hayashi T, Yoshikawa T (2021). Lymph node dissection for gastric cancer: establishment of D2 and the current position of splenectomy in Europe and Japan. Eur. J. Surg. Oncol..

[267] Williams TS, Tallon B, Adams BM (2022). Melanoma sentinel lymph node biopsy and completion lymph node dissection: a regional hospital experience. J. Plast. Reconstr. Aesthet. Surg..

[268] Elhusseini M, Aly EH (2020). Lateral pelvic lymph node dissection in the management of locally advanced low rectal cancer: Summary of the current evidence. Surg. Oncol..

[269] Filetti S (2019). Thyroid cancer: ESMO Clinical Practice Guidelines for diagnosis, treatment and follow-updagger. Ann. Oncol..

[270] McLeod DSA, Sawka AM, Cooper DS (2013). Controversies in primary treatment of low-risk papillary thyroid cancer. Lancet.

[271] Chen L (2018). Prophylactic central neck dissection for papillary thyroid carcinoma with clinically uninvolved central neck lymph nodes: a systematic review and meta-analysis. World J. Surg..

[272] Randolph GW (2012). The prognostic significance of nodal metastases from papillary thyroid carcinoma can be stratified based on the size and number of metastatic lymph nodes, as well as the presence of extranodal extension. Thyroid.

[273] Agrawal N (2017). Indications and extent of central neck dissection for papillary thyroid cancer: an American Head and Neck Society Consensus Statement. Head. Neck.

[274] Hughes DT (2018). Prophylactic central compartment neck dissection in papillary thyroid cancer and effect on locoregional recurrence. Ann. Surg. Oncol..

[275] Sieda B, Tawfik MM, Khatur H (2020). Is routine dissection of central lymph node and radio-active iodine therapy, necessary for papillary thyroid carcinoma, T1-2 N0? A randomized controlled trial. Int. J. Surg. Open.

[276] Mazzaferri EL, Doherty GM, Steward DL (2009). The pros and cons of prophylactic central compartment lymph node dissection for papillary thyroid carcinoma. Thyroid.

[277] Dobrinja C (2017). Rationality in prophylactic central neck dissection in clinically node-negative (cN0) papillary thyroid carcinoma: is there anything more to say? A decade experience in a single-center. Int. J. Surg..

[278] Salem FA, Bergenfelz A, Nordenström E, Almquist M (2021). Central lymph node dissection and permanent hypoparathyroidism after total thyroidectomy for papillary thyroid cancer: population-based study. Br. J. Surg..

[279] Anastasiou OE (2012). Secretory capacity of the parathyroid glands after total thyroidectomy in normocalcemic subjects. J. Clin. Endocrinol. Metab..

[280] Orloff LA (2018). American thyroid association statement on postoperative hypoparathyroidism: diagnosis, prevention, and management in adults. Thyroid.

[281] Kandil E, Noureldine SI, Abbas A, Tufano RP (2013). The impact of surgical volume on patient outcomes following thyroid surgery. Surgery.

[282] Dismukes J (2021). Prophylactic central neck dissection in papillary thyroid carcinoma: all risks, no reward. J. Surg. Res..

[283] Lee DY (2015). The benefits and risks of prophylactic central neck dissection for papillary thyroid carcinoma: prospective cohort study. Int. J. Endocrinol..

[284] Sippel RS (2020). A randomized controlled clinical trial: no clear benefit to prophylactic central neck dissection in patients with clinically node negative papillary thyroid cancer. Ann. Surg..

[285] Sanabria, A., Betancourt, C., Sanchez, J. G. & Garcia, C. Prophylactic central neck lymph node dissection in low-risk thyroid carcinoma patients does not decrease the incidence of locoregional recurrence: a meta-analysis of randomized trials. *Ann. Surg.* **276**, 66–73 (2022). 10.1097/SLA.0000000000005388 35129470

[286] Ahn JH (2022). A prospective randomized controlled trial to assess the efficacy and safety of prophylactic central compartment lymph node dissection in papillary thyroid carcinoma. Surgery.

[287] Tuttle RM, Haugen B, Perrier ND (2017). Updated American Joint Committee on cancer/tumor-node-metastasis staging system for differentiated and anaplastic thyroid cancer (Eighth Edition): what changed and why?. Thyroid.

[288] Sung H (2021). Global cancer statistics 2020: GLOBOCAN estimates of incidence and mortality worldwide for 36 cancers in 185 countries. CA Cancer J. Clin..

[289] Herbst RS, Morgensztern D, Boshoff C (2018). The biology and management of non-small cell lung cancer. Nature.

[290] Liang RB (2018). Incidence and distribution of lobe-specific mediastinal lymph node metastasis in non-small cell lung cancer: data from 4511 resected cases. Ann. Surg. Oncol..

[291] Adachi H (2017). Lobe-specific lymph node dissection as a standard procedure in surgery for non-small cell lung cancer: a propensity score matching study. J. Thorac. Oncol..

[292] Abughararah TZ (2021). Lobe-specific lymph node dissection in stage IA non-small-cell lung cancer: a retrospective cohort study. Eur. J. Cardiothorac. Surg..

[293] Wang Z (2021). Lobe-specific node dissection can be a suitable alternative to systematic lymph node dissection in highly selective early-stage non-small-cell lung cancer patients: a meta-analysis. Ann. Thorac. Cardiovasc. Surg..

[294] Zhao Y (2021). Lobe-specific lymph node dissection in clinical stage IA solid-dominant non-small-cell lung cancer: a propensity score matching study. Clin. Lung Cancer.

[295] Zhang, Y. et al. Selective mediastinal lymph node dissection strategy for clinical T1N0 invasive lung cancer: a prospective, multicenter, clinical trial. *J. Thorac. Oncol*. **18**, 931–939 (2023). 10.1016/j.jtho.2023.02.010 36841542

[296] Ray MA, Smeltzer MP, Faris NR, Osarogiagbon RU (2020). Survival after mediastinal node dissection, systematic sampling, or neither for early stage NSCLC. J. Thorac. Oncol..

[297] Yendamuri S (2018). Effect of the number of lymph nodes examined on the survival of patients with stage I non-small cell lung cancer who undergo sublobar resection. J. Thorac. Cardiovasc. Surg..

[298] Wang W (2019). Impact of different types of lymphadenectomy combined with different extents of tumor resection on survival outcomes of stage I non-small-cell lung cancer: a large-cohort real-world study. Front. Oncol..

[299] Huang W (2022). LobE-Specific lymph node diSsectiON for clinical early-stage non-small cell lung cancer: protocol for a randomised controlled trial (the LESSON trial). BMJ Open.

[300] Hishida T (2018). A randomized phase III trial of lobe-specific vs. systematic nodal dissection for clinical stage I-II non-small cell lung cancer (JCOG1413). Jpn. J. Clin. Oncol..

[301] Moncayo VM, Alazraki AL, Alazraki NP, Aarsvold JN (2017). Sentinel lymph node biopsy procedures. Semin. Nucl. Med..

[302] Magnoni F (2020). Axillary surgery in breast cancer: an updated historical perspective. Semin. Oncol..

[303] Noguchi M (2020). Axillary surgery for breast cancer: past, present, and future. Breast Cancer.

[304] Poulsen L (2021). Comparison of upper extremity lymphedema after sentinel lymph node biopsy and axillary lymph node dissection: patient-reported outcomes in 3044 patients. Breast Cancer Res. Treat..

[305] Schadendorf D (2018). Melanoma. Lancet.

[306] Banting S (2019). Negative sentinel lymph node biopsy in patients with melanoma: the patient’s perspective. Ann. Surg. Oncol..

[307] van der Ploeg AP (2012). Prognosis in patients with sentinel node-positive melanoma without immediate completion lymph node dissection. Br. J. Surg..

[308] Faries MB (2017). Completion dissection or observation for sentinel-node metastasis in melanoma. N. Engl. J. Med..

[309] Lee DY (2016). Impact of completion lymph node dissection on patients with positive sentinel lymph node biopsy in melanoma. J. Am. Coll. Surg..

[310] Satzger I (2014). Is there a therapeutic benefit of complete lymph node dissection in melanoma patients with low tumor burden in the sentinel node?. Melanoma Res..

[311] Susok, L. et al. Waiving subsequent complete lymph node dissection in melanoma patients with positive sentinel lymph node does not result in worse outcome on 20-year analysis. *Cancers* **13**, 5425 (2021). 10.3390/cancers13215425 PMC8582468 34771588

[312] Bello DM, Faries MB (2020). The landmark series: MSLT-1, MSLT-2 and DeCOG (management of lymph nodes). Ann. Surg. Oncol..

[313] Falk Delgado A, Zommorodi S, Falk Delgado A (2019). Sentinel lymph node biopsy and complete lymph node dissection for melanoma. Curr. Oncol. Rep..

[314] Garcia-Etienne CA (2020). Management of the axilla in patients with breast cancer and positive sentinel lymph node biopsy: An evidence-based update in a European breast center. Eur. J. Surg. Oncol..

[315] Galimberti V (2018). Axillary dissection versus no axillary dissection in patients with breast cancer and sentinel-node micrometastases (IBCSG 23-01): 10-year follow-up of a randomised, controlled phase 3 trial. Lancet Oncol..

[316] Japanese Gastric Cancer, A. (2021). Japanese gastric cancer treatment guidelines 2018 (5th edition). Gastric Cancer.

[317] Wu C-W (2006). Nodal dissection for patients with gastric cancer: a randomised controlled trial. Lancet Oncol..

[318] Cuschieri A (1999). Patient survival after D1 and D2 resections for gastric cancer: long-term results of the MRC randomized surgical trial. Surgical Co-operative Group. Br. J. Cancer.

[319] Songun I, Putter H, Kranenbarg EM-K, Sasako M, van de Velde CJH (2010). Surgical treatment of gastric cancer: 15-year follow-up results of the randomised nationwide Dutch D1D2 trial. Lancet Oncol..

[320] Symeonidis D, Diamantis A, Bompou E, Tepetes K (2019). Current role of lymphadenectomy in gastric cancer surgery. J. BUON.

[321] Degiuli M (2021). D2 dissection improves disease-specific survival in advanced gastric cancer patients: 15-year follow-up results of the Italian Gastric Cancer Study Group D1 versus D2 randomised controlled trial. Eur. J. Cancer.

[322] Jiang L (2014). Systematic review and meta-analysis of the effectiveness and safety of extended lymphadenectomy in patients with resectable gastric cancer. Br. J. Surg..

[323] El-Sedfy A (2015). Personalized surgery for gastric adenocarcinoma: a meta-analysis of D1 versus D2 lymphadenectomy. Ann. Surg. Oncol..

[324] Kang JH, Ryu SY, Jung MR, Jeong O (2020). Comparison of long term survival outcomes between D1+ and D2 lymph node dissection for >/= pT2 or pN+ gastric carcinoma: a large scale case-control study using propensity score matching. Eur. J. Surg. Oncol..

[325] Yu P, Du Y, Xu Z, Huang L, Cheng X (2019). Comparison of D2 and D2 plus radical surgery for advanced distal gastric cancer: a randomized controlled study. World J. Surg. Oncol..

[326] Lobo N (2017). Landmarks in the treatment of muscle-invasive bladder cancer. Nat. Rev. Urol..

[327] D’Andrea D (2020). Association of super-extended lymphadenectomy at radical cystectomy with perioperative complications and re-hospitalization. World J. Urol..

[328] Gakis G (2019). Re: extended versus limited lymph node dissection in bladder cancer patients undergoing radical cystectomy: survival results from a prospective, randomized trial. Eur. Urol..

[329] May M (2011). Association between the number of dissected lymph nodes during pelvic lymphadenectomy and cancer-specific survival in patients with lymph node-negative urothelial carcinoma of the bladder undergoing radical cystectomy. Ann. Surg. Oncol..

[330] Chen WS (2020). Novel technique for lymphadenectomy along left recurrent laryngeal nerve during thoracoscopic esophagectomy. World J. Gastroenterol..

[331] Saeki H (2018). "Energy-less technique" with mini-clips for recurrent laryngeal nerve lymph node dissection in prone thoracoscopic esophagectomy for esophageal cancer. Am. J. Surg..

[332] Otsuka K (2020). Minimally invasive esophagectomy and radical lymph node dissection without recurrent laryngeal nerve paralysis. Surg. Endosc..

[333] Wijaya WA, Peng J, He Y, Chen J, Cen Y (2020). Clinical application of axillary reverse mapping in patients with breast cancer: a systematic review and meta-analysis. Breast.

[334] Noguchi M, Inokuchi M, Yokoi-Noguchi M, Morioka E (2022). The involvement of axillary reverse mapping nodes in patients with clinically node-negative breast cancer. Breast Cancer.

[335] Abdelhamid MI, Bari AA, Farid MI, Nour H (2020). Evaluation of axillary reverse mapping (ARM) in clinically axillary node negative breast cancer patients - Randomised controlled trial. Int. J. Surg..

[336] Jain R, Wairkar S (2019). Recent developments and clinical applications of surgical glues: an overview. Int. J. Biol. Macromol..

[337] Ruggiero R (2014). Axillary lymphadenectomy for breast cancer and fibrin glue. Ann. Ital. Chir..

[338] Conversano A (2017). Use of low-thrombin fibrin sealant glue after axillary lymphadenectomy for breast cancer to reduce hospital length and seroma. Clin. Breast Cancer.

[339] Kim YH, Shin HJ, Ju W, Kim SC (2017). Prevention of lymphocele by using gelatin-thrombin matrix as a tissue sealant after pelvic lymphadenectomy in patients with gynecologic cancers: a prospective randomized controlled study. J. Gynecol. Oncol..

[340] McLaughlin SA (2017). Considerations for clinicians in the diagnosis, prevention, and treatment of breast cancer-related lymphedema, recommendations from an expert panel: part 2: preventive and therapeutic options. Ann. Surg. Oncol..

[341] Lawenda BD, Mondry TE, Johnstone PA (2009). Lymphedema: a primer on the identification and management of a chronic condition in oncologic treatment. CA Cancer J. Clin..

[342] Lee HJ, Kane CJ (2014). How to minimize lymphoceles and treat clinically symptomatic lymphoceles after radical prostatectomy. Curr. Urol. Rep..

[343] Liss MA (2013). Outcomes and complications of pelvic lymph node dissection during robotic-assisted radical prostatectomy. World J. Urol..

[344] Singh M, Deo SV, Shukla NK, Pandit A (2011). Chylous fistula after axillary lymph node dissection: incidence, management, and possible cause. Clin. Breast Cancer.

[345] Gerken ALH (2020). Definition and severity grading of postoperative lymphatic leakage following inguinal lymph node dissection. Langenbeck’s Arch. Surg..

[346] Sano Y (2019). Hoarseness after radical surgery with systematic lymph node dissection for primary lung cancer. Eur. J. Cardiothorac. Surg..

[347] Porter DL (2015). Chimeric antigen receptor T cells persist and induce sustained remissions in relapsed refractory chronic lymphocytic leukemia. Sci. Transl. Med..

[348] Leong SP, Naxerova K, Keller L, Pantel K, Witte M (2022). Molecular mechanisms of cancer metastasis via the lymphatic versus the blood vessels. Clin. Exp. Metastasis.

[349] Meadows, K. L. & Hurwitz, H. I. Anti-VEGF therapies in the clinic. *Cold Spring Harb. Perspect. Med.* **2**, a006577 (2012). 10.1101/cshperspect.a006577 PMC3475399 23028128

[350] Morla, S. Glycosaminoglycans and glycosaminoglycan mimetics in cancer and inflammation. *Int. J. Mol. Sci*. **20**, 1963 (2019). 10.3390/ijms20081963 PMC6514582 31013618

[351] Van Raemdonck K, Umar S, Shahrara S (2020). The pathogenic importance of CCL21 and CCR7 in rheumatoid arthritis. Cytokine Growth Factor Rev..

[352] Zhang L (2020). [Progress in targeting therapy of cancer metastasis by CCL21/CCR7 axis]. Sheng Wu Gong. Cheng Xue Bao.

[353] Wiley HE, Gonzalez EB, Maki W, Wu MT, Hwang ST (2001). Expression of CC chemokine receptor-7 and regional lymph node metastasis of B16 murine melanoma. J. Natl Cancer Inst..

[354] Bockorny B (2020). BL-8040, a CXCR4 antagonist, in combination with pembrolizumab and chemotherapy for pancreatic cancer: the COMBAT trial. Nat. Med..

[355] Jones D, Pereira ER, Padera TP (2018). Growth and immune evasion of lymph node metastasis. Front. Oncol..

[356] Baluk P (2007). Functionally specialized junctions between endothelial cells of lymphatic vessels. J. Exp. Med..

[357] Sun L, Zhang H, Gao P (2022). Metabolic reprogramming and epigenetic modifications on the path to cancer. Protein Cell.

[358] Yang Y (2015). Cancer immunotherapy: harnessing the immune system to battle cancer. J. Clin. Invest..

[359] Goode EF, Roussos Torres ET, Irshad S (2021). Lymph node immune profiles as predictive biomarkers for immune checkpoint inhibitor response. Front. Mol. Biosci..

[360] Zhang Y, Zhang Z (2020). The history and advances in cancer immunotherapy: understanding the characteristics of tumor-infiltrating immune cells and their therapeutic implications. Cell Mol. Immunol..

[361] Kennedy LB, Salama AKS (2020). A review of cancer immunotherapy toxicity. CA Cancer J. Clin..

[362] Boyiadzis MM (2018). Chimeric antigen receptor (CAR) T therapies for the treatment of hematologic malignancies: clinical perspective and significance. J. Immunother. Cancer.

[363] Maude SL (2014). Chimeric antigen receptor T cells for sustained remissions in leukemia. N. Engl. J. Med..

[364] Lee DW (2015). T cells expressing CD19 chimeric antigen receptors for acute lymphoblastic leukaemia in children and young adults: a phase 1 dose-escalation trial. Lancet.

[365] Turtle CJ (2016). CD19 CAR-T cells of defined CD4+:CD8+ composition in adult B cell ALL patients. J. Clin. Invest..

[366] Brentjens RJ (2013). CD19-targeted T cells rapidly induce molecular remissions in adults with chemotherapy-refractory acute lymphoblastic leukemia. Sci. Transl. Med..

[367] Davila ML (2014). Efficacy and toxicity management of 19-28z CAR T cell therapy in B cell acute lymphoblastic leukemia. Sci. Transl. Med..

[368] Turtle CJ (2017). Durable molecular remissions in chronic lymphocytic leukemia treated with CD19-specific chimeric antigen receptor-modified T cells after failure of ibrutinib. J. Clin. Oncol..

[369] Porter DL, Levine BL, Kalos M, Bagg A, June CH (2011). Chimeric antigen receptor-modified T cells in chronic lymphoid leukemia. N. Engl. J. Med..

[370] Kochenderfer JN (2012). B-cell depletion and remissions of malignancy along with cytokine-associated toxicity in a clinical trial of anti-CD19 chimeric-antigen-receptor-transduced T cells. Blood.

[371] Kochenderfer JN (2015). Chemotherapy-refractory diffuse large B-cell lymphoma and indolent B-cell malignancies can be effectively treated with autologous T cells expressing an anti-CD19 chimeric antigen receptor. J. Clin. Oncol..

[372] Turtle CJ (2016). Immunotherapy of non-Hodgkin’s lymphoma with a defined ratio of CD8+ and CD4+ CD19-specific chimeric antigen receptor-modified T cells. Sci. Transl. Med..

[373] Kochenderfer JN (2017). Lymphoma remissions caused by anti-CD19 chimeric antigen receptor T cells are associated with high serum interleukin-15 levels. J. Clin. Oncol..

[374] Locke FL (2017). Phase 1 results of ZUMA-1: a multicenter study of KTE-C19 anti-CD19 CAR T cell therapy in refractory aggressive lymphoma. Mol. Ther..

[375] Kochenderfer JN (2017). Long-duration complete remissions of diffuse large B cell lymphoma after anti-CD19 chimeric antigen receptor T cell therapy. Mol. Ther..

[376] Ali SA (2016). T cells expressing an anti-B-cell maturation antigen chimeric antigen receptor cause remissions of multiple myeloma. Blood.

[377] Brudno JN (2018). T cells genetically modified to express an anti-B-cell maturation antigen chimeric antigen receptor cause remissions of poor-prognosis relapsed multiple myeloma. J. Clin. Oncol..

[378] Mikkilineni L, Kochenderfer JN (2017). Chimeric antigen receptor T-cell therapies for multiple myeloma. Blood.

[379] Brudno JN, Kochenderfer JN (2019). Recent advances in CAR T-cell toxicity: mechanisms, manifestations and management. Blood Rev..

[380] Zou W, Chen L (2008). Inhibitory B7-family molecules in the tumour microenvironment. Nat. Rev. Immunol..

[381] Li, Y. et al. A mini-review for cancer immunotherapy: molecular understanding of PD-1/PD-L1 pathway & translational blockade of immune checkpoints. *Int. J. Mol. Sci.* **17**, 1151 (2016). 10.3390/ijms17071151 PMC4964524 27438833

[382] Yi M (2022). Combination strategies with PD-1/PD-L1 blockade: current advances and future directions. Mol. Cancer.

[383] Morse MA, Gwin WR, Mitchell DA (2021). Vaccine therapies for cancer: then and now. Target Oncol..

[384] Hollingsworth RE, Jansen K (2019). Turning the corner on therapeutic cancer vaccines. NPJ Vaccines.

[385] Saxena M, van der Burg SH, Melief CJM, Bhardwaj N (2021). Therapeutic cancer vaccines. Nat. Rev. Cancer.

[386] Ma M, Liu J, Jin S, Wang L (2020). Development of tumour peptide vaccines: from universalization to personalization. Scand. J. Immunol..

[387] Obinu A (2018). Nanoparticles in detection and treatment of lymph node metastases: an update from the point of view of administration routes. Expert Opin. Drug Deliv..

[388] Morisaki, T. et al. Lymph nodes as anti-tumor immunotherapeutic tools: intranodal-tumor-specific antigen-pulsed dendritic cell vaccine immunotherapy. *Cancers* **14**, 2438 (2022). 10.3390/cancers14102438 PMC9140043 35626042

[389] Kipp JE (2004). The role of solid nanoparticle technology in the parenteral delivery of poorly water-soluble drugs. Int. J. Pharm..

[390] Zhang L (2008). Self-assembled lipid-polymer hybrid nanoparticles: a robust drug delivery platform. ACS Nano.

[391] Whitehead KA, Langer R, Anderson DG (2009). Knocking down barriers: advances in siRNA delivery. Nat. Rev. Drug Discov..

[392] Alexis F, Pridgen E, Molnar LK, Farokhzad OC (2008). Factors affecting the clearance and biodistribution of polymeric nanoparticles. Mol. Pharm..

[393] Bertrand N, Leroux JC (2012). The journey of a drug-carrier in the body: an anatomo-physiological perspective. J. Control Release.

[394] Bertrand N, Wu J, Xu X, Kamaly N, Farokhzad OC (2014). Cancer nanotechnology: the impact of passive and active targeting in the era of modern cancer biology. Adv. Drug Deliv. Rev..

[395] Cote B, Rao D, Alany RG, Kwon GS, Alani AWG (2019). Lymphatic changes in cancer and drug delivery to the lymphatics in solid tumors. Adv. Drug Deliv. Rev..

[396] Nakamura T (2020). The effect of size and charge of lipid nanoparticles prepared by microfluidic mixing on their lymph node transitivity and distribution. Mol. Pharm..

[397] Shah S (2022). Nanotechnology based drug delivery systems: Does shape really matter?. Int. J. Pharm..

[398] Cheng, Z., Que, H., Chen, L., Sun, Q. & Wei, X. Nanomaterial-based drug delivery system targeting lymph nodes. *Pharmaceutics* **14**, 1372 (2022). 10.3390/pharmaceutics14071372 PMC9325242 35890268

[399] McLennan DN, Porter CJ, Charman SA (2005). Subcutaneous drug delivery and the role of the lymphatics. Drug Discov. Today Technol..

[400] Cho, K. J., Cho, Y. E. & Kim, J. Locoregional lymphatic delivery systems using nanoparticles and hydrogels for anticancer immunotherapy. *Pharmaceutics* **14**, 2752 (2022). 10.3390/pharmaceutics14122752 PMC9788085 36559246

[401] Ryan GM, Kaminskas LM, Porter CJ (2014). Nano-chemotherapeutics: maximising lymphatic drug exposure to improve the treatment of lymph-metastatic cancers. J. Control Release.

[402] Makino J (2015). cRGD-installed polymeric micelles loading platinum anticancer drugs enable cooperative treatment against lymph node metastasis. J. Control Release.

[403] Singh N, Handa M, Singh V, Kesharwani P, Shukla R (2022). Lymphatic targeting for therapeutic application using nanoparticulate systems. J. Drug Target..

[404] Laakkonen P, Porkka K, Hoffman JA, Ruoslahti E (2002). A tumor-homing peptide with a targeting specificity related to lymphatic vessels. Nat. Med..

[405] Song N (2020). LyP-1-modified multifunctional dendrimers for targeted antitumor and antimetastasis therapy. ACS Appl. Mater. Interfaces.

[406] Li F (2015). Evaluation of (99m)Tc-HYNIC-TMTP1 as a tumor-homing imaging agent targeting metastasis with SPECT. Nucl. Med. Biol..

[407] Wei R (2019). TMTP1-modified indocyanine green-loaded polymeric micelles for targeted imaging of cervical cancer and metastasis sentinel lymph node in vivo. Theranostics.

[408] Murphy EA (2008). Nanoparticle-mediated drug delivery to tumor vasculature suppresses metastasis. Proc. Natl Acad. Sci. USA.

[409] Lecocq Q (2019). Theranostics in immuno-oncology using nanobody derivatives. Theranostics.

[410] Caballero D (2022). Precision biomaterials in cancer theranostics and modelling. Biomaterials.

[411] Oh KS (2014). Accurate sequential detection of primary tumor and metastatic lymphatics using a temperature-induced phase transition nanoparticulate system. Int. J. Nanomed..

[412] Cai W (2020). Self-assembled hybrid nanocomposites for multimodal imaging-guided photothermal therapy of lymph node metastasis. ACS Appl Mater. Interfaces.

[413] Huang X (2012). Long-term multimodal imaging of tumor draining sentinel lymph nodes using mesoporous silica-based nanoprobes. Biomaterials.

[414] Tseng YC, Xu Z, Guley K, Yuan H, Huang L (2014). Lipid-calcium phosphate nanoparticles for delivery to the lymphatic system and SPECT/CT imaging of lymph node metastases. Biomaterials.

[415] Partridge SC, Kurland BF, Liu CL, Ho RJ, Ruddell A (2015). Tumor-induced lymph node alterations detected by MRI lymphography using gadolinium nanoparticles. Sci. Rep..

[416] Spaliviero M (2016). Detection of lymph node metastases with SERRS nanoparticles. Mol. Imaging Biol..

[417] Tang L (2012). Aptamer-functionalized, ultra-small, monodisperse silica nanoconjugates for targeted dual-modal imaging of lymph nodes with metastatic tumors. Angew. Chem..

[418] Qiao R (2015). Ultrasensitive in vivo detection of primary gastric tumor and lymphatic metastasis using upconversion nanoparticles. ACS Nano.

[419] Yang X (2017). Mapping sentinel lymph node metastasis by dual-probe optical imaging. Theranostics.

[420] Xu G (2019). Long-distance tracing of the lymphatic system with a computed tomography/fluorescence dual-modality nanoprobe for surveying tumor lymphatic metastasis. Bioconjug. Chem..

[421] Liu L (2019). Ultrasmall superparamagnetic nanoparticles targeting E-selectin: synthesis and effects in mice in vitro and in vivo. Int. J. Nanomed..

[422] Dai Y (2020). Metastatic status of sentinel lymph nodes in breast cancer determined with photoacoustic microscopy via dual-targeting nanoparticles. Light Sci. Appl..

[423] Feng X, Li Y, Zhang S, Li C, Tian J (2022). Quantitative hypoxia mapping using a self-calibrated activatable nanoprobe. J. Nanobiotechnol..

[424] Han J, Zhang L, Cui M, Su Y, He Y (2021). Rapid and accurate detection of lymph node metastases enabled through fluorescent silicon nanoparticles-based exosome probes. Anal. Chem..

[425] Japanese Gastric Cancer, A. (2017). Japanese gastric cancer treatment guidelines 2014 (ver. 4). Gastric Cancer.

